# A genetic association study of glutamine-encoding DNA sequence structures, somatic CAG expansion, and DNA repair gene variants, with Huntington disease clinical outcomes

**DOI:** 10.1016/j.ebiom.2019.09.020

**Published:** 2019-10-10

**Authors:** Marc Ciosi, Alastair Maxwell, Sarah A. Cumming, Davina J. Hensman Moss, Asma M. Alshammari, Michael D. Flower, Alexandra Durr, Blair R. Leavitt, Raymund A.C. Roos, the TRACK-HD team, the Enroll-HD team, Peter Holmans, Lesley Jones, Douglas R. Langbehn, Seung Kwak, Sarah J. Tabrizi, Darren G. Monckton

**Affiliations:** a Institute of Molecular, Cell and Systems Biology, College of Medical, Veterinary and Life Sciences, University of Glasgow, Glasgow G12 8QQ, UK; b Huntington's Disease Centre, Department of Neurodegenerative Disease, Institute of Neurology, University College London, London, UK; c APHP Department of Genetics, Pitié-Salpêtrière University Hospital, Paris, France; d ICM, Institut du Cerveau et de la Moelle, INSERM U1127, CNRS UMR7225, Sorbonne Université, Paris, France; e Centre for Molecular Medicine and Therapeutics, Department of Medical Genetics, Child and Family Research Institute, University of British Columbia, Vancouver, Canada; f Department of Neurology, Leiden University Medical Centre, Leiden, the Netherlands; g Medical Research Council Centre for Neuropsychiatric Genetics and Genomics, Department of Psychological Medicine and Neurology, School of Medicine, Cardiff University, Cardiff, UK; h Departments of Psychiatry and Biostatistics, University of Iowa, Iowa City, IA, USA; i CHDI Management/CHDI Foundation, Princeton, NJ, USA; j UK Dementia Research Institute at UCL, London, UK

**Keywords:** Genetic association study, Somatic expansion, DNA repair, Huntington disease

## Abstract

**Background:**

Huntington disease (HD) is caused by an unstable CAG/CAA repeat expansion encoding a toxic polyglutamine tract. Here, we tested the hypotheses that HD outcomes are impacted by somatic expansion of, and polymorphisms within, the *HTT* CAG/CAA glutamine-encoding repeat, and DNA repair genes.

**Methods:**

The sequence of the glutamine-encoding repeat and the proportion of somatic CAG expansions in blood DNA from participants inheriting 40 to 50 CAG repeats within the TRACK-HD and Enroll-HD cohorts were determined using high-throughput ultra-deep-sequencing. Candidate gene polymorphisms were genotyped using kompetitive allele-specific PCR (KASP). Genotypic associations were assessed using time-to-event and regression analyses.

**Findings:**

Using data from 203 TRACK-HD and 531 Enroll-HD participants, we show that individuals with higher blood DNA somatic CAG repeat expansion scores have worse HD outcomes: a one-unit increase in somatic expansion score was associated with a Cox hazard ratio for motor onset of 3·05 (95% CI = 1·94 to 4·80, *p* = 1·3 × 10^−6^). We also show that individual-specific somatic expansion scores are associated with variants in *FAN1* (*pFDR = *4·8 × 10^-6^), *MLH3* (*pFDR = *8·0 × 10^−4^), *MLH1* (*pFDR = *0·004) and *MSH3* (*pFDR = *0·009). We also show that HD outcomes are best predicted by the number of pure CAGs rather than total encoded-glutamines.

**Interpretation:**

These data establish pure CAG length, rather than encoded-glutamine, as the key inherited determinant of downstream pathophysiology. These findings have implications for HD diagnostics, and support somatic expansion as a mechanistic link for genetic modifiers of clinical outcomes, a driver of disease, and potential therapeutic target in HD and related repeat expansion disorders.

**Funding:**

CHDI Foundation.

## Research in context

### Evidence before this study

Huntington disease (HD), a devastating neurodegenerative condition with a prevalence of ∼1/10000, is caused by the expansion of a CAG/CAA repeat in exon one of the *HTT* gene. The expanded CAG/CAA tract encodes for glutamine in the HTT protein, and pathology is assumed to primarily result as a toxic gain of function of the mutant polyglutamine containing protein. More repeats cause an earlier age at onset, however, measured inherited CAG length only accounts for ∼60% of the variation in age at onset. We sought to identify genetic variants that modify residual variation in HD outcomes not accounted for by measured CAG length and determine their mode of action. All searches were conducted in PubMed for articles published from January 1st 1984 until April 30th 2019. Language was not restricted, however, PubMed is biased toward English language journals. Nonetheless, although rare, HD is a high-profile disorder and recent important genetic studies are likely to have been published in English. Initially, we used the search terms “Huntington* disease” AND “modifier gene” AND species “human” AND publication type NOT “review”. This identified 53 reports, most of which were candidate gene studies that were superseded by two genome-wide association studies (GWAS). These revealed genome-wide significant hits at the *RRM2B/UBR5, FAN1* and *MSH3* loci, and a highly suggestive hit at the *MLH1* locus that was confirmed in a follow-up study. As some of these genes are known modifiers of somatic expansion in animal models, these data suggested that their effects on disease severity might be mediated through somatic expansion of the CAG repeat. We thus also used the search terms “Huntington* disease” AND “somatic expansion” AND species “human” and publication type NOT “review”. This identified 55 reports, most of which were studies in other diseases or model organisms, or small-scale largely non-quantitative analyses of somatic expansion in HD patients. Several critical studies reported large expansions in HD patient brains, with one key study revealing an association between the degree of skewness of somatic expansions in the cortex of end-stage patients and extreme variation in age at onset. There were no large-scale systematic quantitative analyses of the frequency of somatic expansions in the peripheral tissues of HD patients. It is well known in other disorders that the presence of variant repeat interruptions within the repeat array can modulate the germline instability of repeat expansions. Using the search terms “Huntington* disease” AND “repeat interruption” AND species “human” and publication type NOT “review” revealed no studies. However, we were aware of two 1995 reports by Pêcheux et al., and Goldberg et al., describing atypical structures of the *HTT* exon one CAG/CAA repeat. These studies were subsequently cited in 146 publications, eight of which described rare sequence variants in the *HTT* exon one repeat. Articles citing these studies were investigated, revealing one additional study describing an atypical *HTT* exon one repeat allele. All of these studies were small, mostly anecdotal case reports, and none provided any quantitative data in support of an effect on HD clinical outcomes. Some available evidence suggests that loss of the CAA repeat interruption might be associated with an increased risk of intergenerational expansion of intermediate alleles (27 to 35 CAG repeats), but none of the studies was powered to provide a definitive answer to this question. None of the studies reported any effect of the presence or absence of repeat sequence interruptions on the somatic instability of *HTT* alleles.

### Added value of this study

We present a high-throughput ultra-deep sequencing method to reveal the frequency of synonymous CAA repeat interruptions in the *HTT* CAG/CAA repeat and determined that pure CAG length, not total encoded-glutamine number, better explains clinical outcomes in HD, and that mis-sizing of the expanded pure CAG tract using standard fragment length may yield misleading estimates of predicted HD outcomes. We precisely quantified the ratio of somatic CAG expansions in blood DNA, and revealed that somatic expansions are also better predicted by pure CAG length, and that individuals with more somatic expansions tend to have worse clinical outcomes, including an earlier age at onset, higher baseline total motor score and, in TRACK-HD, higher progression scores. We also established that variants in DNA repair genes *FAN1, MSH3* and *MLH1,* previously identified by GWASs of HD onset and progression, and *MLH3,* previously implicated by pathway analysis and animal models, are associated with somatic expansion scores in blood DNA.

### Implications of all the available evidence

These data suggest that consideration should be given in the future to potentially replacing fragment length analysis using a sequence-based approach to genotyping HD in order to improve diagnostic accuracy, prognostic precision and the power of clinical trials. This might be particularly clinically relevant for people with repeat lengths close to the pathological threshold, as a sequence-based approach would reduce ambiguity and the risk of false-positive/negative test results. Combined with previous data on the intergenerational instability of *HTT* alleles lacking CAA repeat interruptions, these data suggest that sequence-level genotypes will also enable provision of more accurate risk assessments to offspring for prospective parents. These data reveal CAG length as a more accurate predictor of disease outcomes rather than encoded-glutamine repeat length. This has implications for understanding the pathogenic events in HD, and the interpretation of apparently disease-moderating repeat interruptions in related disorders also caused by the expansion of polyglutamine-encoding CAG repeats which may also act through effects on somatic expansion rather than the assumed effects on protein structure. They also support a novel approach to therapy via gene editing, by introduction of synonymous DNA-stabilising interruptions. Our data establish somatic expansion in blood DNA as a molecular phenotype, and, combined with previous data on somatic expansion of the *HTT* CAG repeat in brain and animal models, suggest that some genetic modifiers of HD clinical outcomes may operate via effects on somatic expansion of the CAG repeat. Combined, these data support somatic expansion of the CAG repeat as a novel therapeutic target in HD, and likely in other repeat expansion disorders, reveal a potential peripheral biomarker of somatic expansion for clinical trials, and highlight FAN1, MLH1, MSH3 and MLH3 as potential drug targets.

## 1 Introduction

Huntington disease (HD) is one of a number of disorders including many of the spinocerebellar ataxias, dentatorubral pallidoluysian atrophy, and spinal and bulbar muscular atrophy, caused by the expansion of a genetically unstable CAG/CAA repeat encoding a toxic polyglutamine protein and characterised by extreme variability in age at onset (AAO) [1]. HD is caused by the expansion of a CAG repeat in exon one of *HTT* and the most common allele in Europeans is 17 CAG repeats. HD-associated alleles exceed 35 CAG repeats and penetrance increases to ∼100% by 40 repeats [2], [3]. HD alleles are unstable in the germline and frequently increase in length from one generation to the next, with inheritance of a single CAG increase reducing AAO by ∼2 years [2]. Disease-associated alleles also display allele length-, age- and cell type-dependent, and expansion-biased, somatic instability, with somatic expansions exceeding 1000 CAG repeats in some striatal neurons [4], [5], [6], [7], [8]. Although somatic expansion could provide an explanation for the tissue-specificity and progressive nature of symptoms [4], it has not been widely considered as a therapeutic target in HD [9] and related disorders. However, recent data arising from an unbiased genome-wide association study (GWAS) revealed signals in DNA repair pathways as modifiers of age at onset [10], some of which have previously been revealed as mediating somatic expansions in mouse models [1].

In a typical *HTT* allele, the pure CAG repeat (CAG copy number = Q^1^) is followed immediately downstream by an additional glutamine-encoding CAACAG cassette (number of additional glutamines encoded by this region = Q^2^, Fig. 2, Table 1). Thus, the total number of consecutive glutamines encoded by this region (Q^T^) is typically equal to the number of pure CAGs, plus two (Q^T^ = Q^1^ + Q^2^). The standard approach to genotyping in HD is to use PCR and fragment length analysis to infer the number of pure CAGs (Q^FL^) assuming the typical allele structure (*i.e.* Q^1^ = Q^FL^, assuming Q^2^ = 2) [11]. Sequence variants in which the CAACAG cassette is either deleted (Q^2^ = 0), or duplicated (Q^2^ = 4), have been reported (Table S1, appendix). In these cases, CAG length estimated by fragment length analysis may not equal the number of pure CAGs (*i.e.* Q^FL^ ≠ Q^1^). The glutamine-encoding CAG/CAA tract is followed downstream by a proline-encoding CCA/CCG/CCT repeat tract, that although polymorphic (Fig. 2, Table 1), has no apparent impact on HD AAO [12].

**Table 1:** Allele structures observed at the *HTT* exon one repeat locus.

Allele structure code Q^1^-Q^2^-P^1^-P^2^-P^3^	DNA sequence Variant annotation (CAG)_Q1_(CAACAG)_Q2/2_(CCGCCA)_P1/2_(CCG)_P2_(CCT)_P3_	Glutamines Total (Q^1^ + Q^2^)	Prolines Total (P^1^ + P^2^ + P^3^)	Allele frequency (%) [*n* individuals]			
				TRACK-HD [203]		Enroll-HD [543]	
				Non-disease	Disease	Non-disease	Disease
Q^1^-2-2-P^2^-2	(CAG)_Q1_(CAACAG)_1_(CCGCCA)_1_(CCG)_P2_(CCT)_2_	Q^1^ + 2	P^2^ + 4	94·09 [191]	96·06 [195]	91·53 [497]	97·61 [530]
	LRG_763:c.52_153CAG[Q1]CAACAG[1]CCGCCA[1]CCG[P2]CCT[2]						
Q^1^-4-2-7-3	(CAG)_Q1_(CAACAG)_2_(CCGCCA)_1_(CCG)_7_(CCT)_3_	Q^1^ + 4	P^2^ + 5	3·86 [8]	2·96 [6]	2·95 [16]	1·29 [7]
	LRG_763:c.52_153CAG[Q1]CAACAG[2]CCGCCA[1]CCG[7]CCT[3]						
17-2-0-6-2	(CAG)_17_(CAACAG)_1_(CCGCCA)_0_(CCG)_6_(CCT)_2_	Q^1^ + 2	P^2^ + 2	-	-	0·18 [1]	-
	LRG_763:c.52_153CAG[17]CAACAG[1]CCGCCA[0]CCG[6]CCT[2]						
Q^1^-2-0-9-2	(CAG)_Q1_(CAACAG)_1_(CCGCCA)_0_(CCG)_9_(CCT)_2_	Q^1^ + 2	P^2^ + 2	1·48 [3]	-	4·97 [27]	-
	LRG_763:c.52_153CAG[Q1]CAACAG[1]CCGCCA[0]CCG[9]CCT[2]						
Q^1^-2-2-9-3	(CAG)_Q1_(CAACAG)_1_(CCGCCA)_1_(CCG)_9_(CCT)_3_	Q^1^ + 2	P^2^ + 5	0·49 [1]	-	0·18 [1]	-
	LRG_763:c.52_153CAG[Q1]CAACAG[1]CCGCCA[1]CCG[9]CCT[3]						
Q^1^-0-2-7-2	(CAG)_Q1_(CAACAG)_0_(CCGCCA)_1_(CCG)_7_(CCT)_2_	Q^1^	P^2^ + 4	-	0·49 [1]	-	0·55 [3]
	LRG_763:c.52_153CAG[Q1]CAACAG[0]CCGCCA[1]CCG[7]CCT[2]						
20-0-2-11-2	(CAG)_20_(CAACAG)_0_(CCGCCA)_1_(CCG)_11_(CCT)_2_	Q^1^	P^2^ + 4	-	-	0·18 [1]	-
	LRG_763:c.52_153CAG[20]CAACAG[0]CCGCCA[1]CCG[11]CCT[2]						
41-0-0-9-2	(CAG)_41_(CAACAG)_0_(CCGCCA)_0_(CCG)_9_(CCT)_2_	Q^1^	P^2^ + 2	-	0·49 [1]	-	-
	LRG_763:c.52_153CAG[41]CAACAG[0]CCGCCA[0]CCG[9]CCT[2]						
Q^1^-0-0-11-2	(CAG)_Q1_(CAACAG)_0_(CCGCCA)_0_(CCG)_11_(CCT)_2_	Q^1^	P^2^ + 2	-	-	-	0·37 [2]
	LRG_763:c.52_153CAG[Q1]CAACAG[0]CCGCCA[0]CCG[11]CCT[2]						
41-3-2-7-2	(CAG)_41_(CAA)_1_(CAACAG)_1_(CCGCCA)_1_(CCG)_7_(CCT)_2_	Q^1^ + 3	P^2^ + 4	-	-	-	0·18 [1]
	LRG_763:c.52_153CAG[41]CAA[1]CAACAG[1]CCGCCA[1]CCG[7]CCT[2]						

See Fig. 2 for further details of allele structures. Q^1^ = number of pure CAGs; Q^2^ = the number of downstream glutamine-encoding CAA/CAG repeats; P^1^ = number of CCG/CCA proline codons upstream of the pure CCG repeat tract; P^2^ = number of pure CCGs; P^3^ = number of CCT repeats. Disease-associated alleles with either zero, three or four downstream glutamine codons (Q^2^ = 0, 3 or 4), are indicated with a red upward triangle, green diamond, or green downward triangle, respectively. The variant annotation describes the *HTT* exon one sequence variants using the Sequence Variant Nomenclature of the Human Genome Variation Society based on the *HTT* Locus Reference Genomic sequence LRG_763.

In other repeat expansion disorders, variant repeat interruptions modify mutational dynamics and disease severity [1]. We hypothesised that CAG repeat sequence variants in *HTT* exon one, and DNA repair gene variants, may directly modify somatic expansion and consequent HD outcomes. Here, we sought to test these hypotheses by high-throughput ultra-deep DNA sequencing to reveal allele structure and quantify somatic expansions in individuals carrying disease-associated *HTT* alleles and to correlate these data with HD clinical outcomes and DNA repair gene variants.

## 2 Methods

### 2.1 Study design and populations

The aim of the study was to sequence the *HTT* CAG/CAA repeat, quantify somatic expansions, genotype candidate DNA repair gene polymorphisms and correlate these data with HD clinical outcomes. As a discovery cohort, we selected 203 participants carrying disease-associated *HTT* alleles with from 40 to 50 CAG repeats with progression scores from TRACK-HD (105 premanifest, and 98 manifest for motor symptoms) (Table 2) (Supplementary methods in appendix). As a replication cohort, we selected 543 participants carrying disease-associated *HTT* alleles with from 40 to 50 CAG repeats from Enroll-HD, of which 531 had all of the data available for genotype to phenotype analyses (141 premanifest, and 390 manifest for motor symptoms) (Table 2) (Supplementary methods in appendix) [13].

**Table 2:** Participant characteristics**.** Participants excluded from the data analyses are not included.

Characteristics	TRACK-HD	Enroll-HD	TRACK-HD and Enroll-HD
Number of individuals	203	531	734
Range of age at DNA sampling (years)	18·63 to 64·13	18·42 to 75·50	18·42 to 75·50
Mean age at DNA sampling (years (SD))	45·00 (9·93)	48·84 (12·25)	47·78 (11·77)
Range of age at baseline (years)	18·63 to 64·13	18·42 to 75·50	18·42 to 75·50
Mean age at baseline (years (SD))	44·96 (9·90)	48·84 (12·25)	47·77 (11·77)
Sex = female (%)	54·68	53·67	53·95
Manifest for Huntington disease motor symptoms	48·28%	73·45%	66·49%
Mean age at onset of motor symptoms (years (SD))	45·27 (8·89)	45·85 (10·72)	45·73 (10·37)
Mean total motor score at baseline (SD)	12·06 (12·64)	23·58 (20·40)	20·40 (19·27)
Mean total functional capacity at baseline (SD)	11·93 (1·73)	NA	NA
Mean pure CAG (Q^1^) in the disease-associated allele (SD)	43·14 (2·16)	43·25 (2·40)	43·22 (2·33)
Mean total encoded glutamine (Q^T^) in the disease-associated allele (SD)	45·18 (2·25)	45·26 (2·42)	45·24 (2·37)
Mean fragment length estimate of CAG (Q^FL^) in the disease-associated allele (SD)	43·15 (2·21)	43·25 (2·42)	43·22 (2·36)

### 2.2 Procedures

Participants were recruited to TRACK-HD between 2008 and 2011 [14], and Enroll-HD from 2012 [13]. Clinical and genetic data were obtained from CHDI, and new genetic data generated, for TRACK-HD participants between October 2015 and October 2016, and Enroll-HD between June 2016 and December 2017. The primary HD clinical outcomes we considered in our analyses were: motor AAO; Unified Huntington's Disease Rating Scale (UHDRS) total motor score (TMS) [15]; UHDRS total functional capacity (TFC) [15]; and TRACK-HD progression score (Supplementary methods in appendix) [14].

### 2.3 *HTT* exon one repeat region sequencing and genotyping

The *HTT* exon one repeat region was amplified from 20 ng of blood DNA using MiSeq-compatible PCR primers, sequenced on MiSeq [16] and genotyped using ScaleHD (v0.251) (Supplementary methods in appendix). The ratio of somatic CAG expansions $\left(\frac{\mathrm{numb}\mathrm{er}\mathrm{of}\mathrm{soma}\mathrm{tic}\mathrm{expa}\mathrm{nsion}\mathrm{prod}\mathrm{ucts}}{\mathrm{numb}\mathrm{er}\mathrm{of}\mathrm{prog}\mathrm{enit}\mathrm{or}\mathrm{alle}\mathrm{le}\mathrm{prod}\mathrm{ucts}}\right)$ was quantified from the MiSeq read count distributions (see details in Supplementary methods in appendix).

### 2.4 Selection of candidate SNPs and genotyping

Full details of candidate SNP selection and genotyping are provided in section I.1.9 of the appendix.

### 2.5 Statistical analyses

**Fig. 1:**
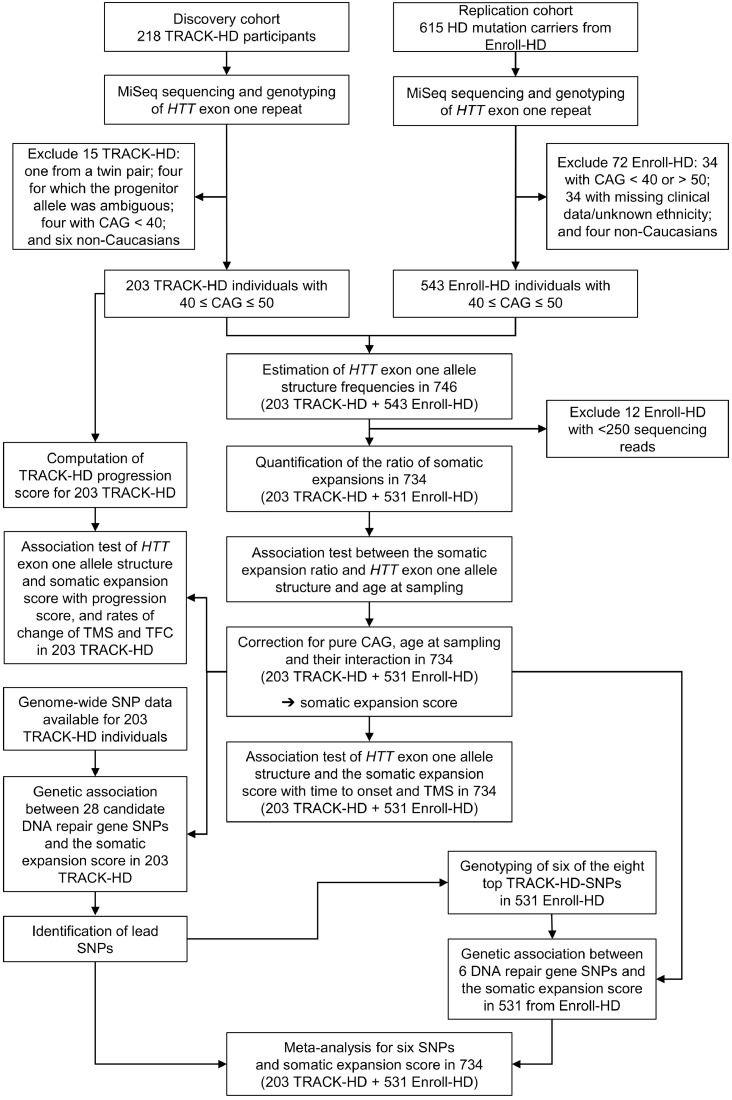
Study design and sample exclusions.

Statistical analyses were undertaken in R (v3.4.3) [17] using RStudio (version 1.0.153, see appendix for a full a list of R functions and packages used) [18]. All individuals are represented once in each analysis. Each data point represents a single baseline value for each participant except for three-year longitudinal data available in TRACK-HD for TMS and TFC. These longitudinal TMS and TFC data were used to express the influence of Q^2^ and the somatic expansion score on the progression score in units of clinical measurements that are more commonly used (*i.e*. TMS and TFC). To do so, participant-specific slopes derived from mixed effect models with correlated random intercepts and slopes (with a fixed ‘years of follow-up’ effect and a random ‘participant’ effect) were used as participant-specific rate of change in TMS and TFC (TMSrate and TFCrate). Multiple linear regressions were performed to investigate the association of ‘CAG’ length (Q^T^ or Q^1^), number of additional glutamine codons (Q^2^), and age, with the ratio of somatic CAG expansions (models SEQ^T^, SEQ^1^ and SEQ^1^Q^2^), and derive an age- and pure CAG (Q^1^)-corrected individual-specific somatic expansion score. Multivariate time-to-event analyses were carried out using stratified Cox proportional hazard regressions to investigate the association of ‘CAG’ length (Q^T^, Q^1^ or Q^FL^), number of additional glutamine codons (Q^2^) and the somatic expansion score with the time-to-onset of HD motor symptoms (models AAOQ^T^, AAOQ^1^, AAOQ^1^Q^2^ and AAOQ^FL^Q^2^). For individuals without HD motor onset, the censored time was the age at the last TRACK-HD or Enroll-HD periodic dataset 3 visit. Cohort and sex were considered as strata in all Cox regressions. We confirmed that the proportional hazard assumption could be assumed for each Cox regression model fit and each covariate (*p* > 0·106). To derive relative effect sizes, adjusted survival curves and median time to HD motor onset for the different numbers of additional glutamine codon categories (Q^2^), and positive and negative somatic expansion scores, were estimated based on the mean values of the other covariates. Multiple linear regressions were performed to investigate the association of ‘CAG’ length (Q^T^, Q^1^ or Q^FL^), number of additional glutamine codons (Q^2^), age and the somatic expansion score with the progression score (models ProgQ^1^ and ProgQ^FL^), TMSrate (model TMSrateQ^1^Q^2^), TFCrate (model TFCrateQ^1^Q^2^) and baseline TMS (models TMSQ^T^, TMSQ^1^, TMSQ^1^Q^2^ and TMSQ^FL^Q^2^). In the Cox and linear regression analyses, all continuous explanatory variables (Q^T^, Q^1^, Q^FL^, age, baseline TMS, and baseline TFC) were centred, except for the somatic expansion score, which, as a regression residual was effectively already quasi-centred. We compared the goodness of fit of regression models containing the same degrees of freedom (*i.e.* regression with Q^T^ vs regression including Q^1^) via nonparametric bootstrapping of the *r*^2^ (for multiple linear regressions) or the log-likelihood (for Cox proportional hazard regressions) statistic. We performed 5000 bootstrap replications and estimated the confidence interval via the bias-corrected accelerated (BCA) method [19], [20]. ANOVA was used to compare regression models containing, or not containing, the number of additional encoded-glutamines (Q^2^) as a covariate (model SEQ^1^ compared to SEQ^1^Q^2^, model AAOQ^1^ compared to AAOQ^1^Q^2^, and model TMSQ^1^ compared to TMSQ^1^Q^2^). For each comparison, the *p*-value associated with the ANOVA test-statistic (*F* for linear regressions and *χ*^2^ for Cox regressions) was then estimated based on 10^5^ permutations of the number of additional encoded-glutamines (Q^2^). In order to avoid potential biases in *p*-value estimates due to small sample sizes for some levels of the number of additional glutamine codons (Q^2^), for all linear and Cox regressions containing the number of additional glutamine codons (Q^2^) as a covariate (*i.e.* SEQ^1^Q^2^, AAOQ^1^Q^2^, AAOQ^FL^Q^2^, ProgQ^1^, ProgQ^FL^, TMSrateQ^1^Q^2^, TFCrateQ^1^Q^2^, TMSQ^1^Q^2^, TMSQ^FL^Q^2^), all the Q^2^-associated *p*-values were estimated based on the *t-* (for the linear regressions) or *z*-statistic (for the Cox regressions) using 10^5^ permutations of the number of additional glutamine codons (Q^2^). The effect size for each number of additional glutamine codons (Q^2^) genotype on the progression score, TMSrate, TFCrate, and baseline TMS, was derived from the least-square means (*i.e.* the means for each number of additional glutamine codons (Q^2^) genotype adjusted for all other variables in the linear regression). All statistical analyses were two-sided and included sex, cohort and age at baseline (when appropriate) as covariates. Participants with missing data were excluded from relevant analyses (Fig. 1). PLINK (v1.07) [21] and gPLINK (version 2.050; http://zzz.bwh.harvard.edu/plink/gplink.shtml) were used to test for Hardy-Weinberg equilibrium and the association between SNPs and somatic expansion score by linear regression with sex as a covariate. Meta-analyses of the association between SNPs and somatic expansion score were performed using sample size-weighted analysis (based on *p*-values) in METAL [22]. Within each set of analyses (AAO, progression score, rate of change of TMS and TFC, baseline TMS, SNP association), the Benjamini-Hochberg false discovery rate (FDR) procedure was used to correct for multiple testing [23].

## 3 Results

**Fig. 2:**
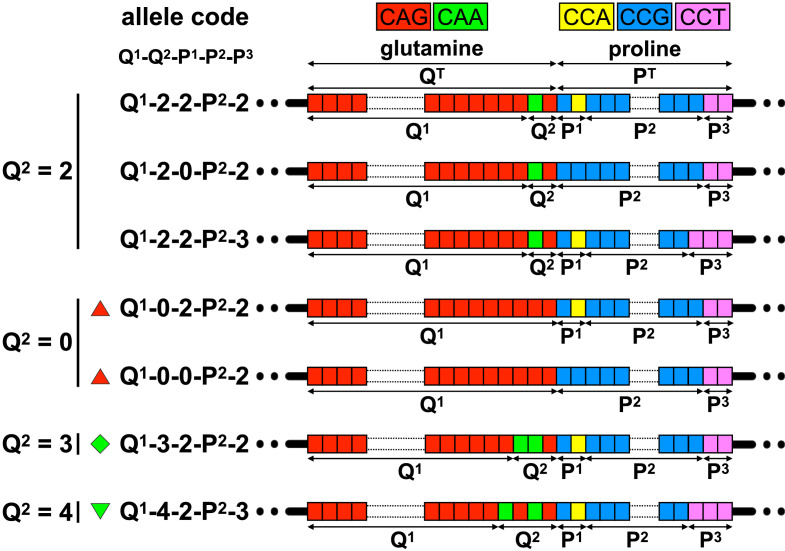
Allelic variation at the *HTT* exon one repeat locus**.** The schematic diagram shows the typical reference allele structure of the CAG/CCG repeat region in exon one of *HTT* (top). We here define the number of pure CAGs as Q^1^ and the number of additional downstream glutamine-encoding CAA/CAG repeats as Q^2^. Thus, total encoded-glutamine Q^T^ = Q^1^ + Q^2^. We here define the number of copies of the proline codons before the pure CCG repeat as P^1^, the number of pure CCGs as P^2^, and the number of copies of the CCT proline codons as P^3^. Thus, total encoded-proline P^T^ = P^1^ + P^2^ + P^3^. The figure shows schematic representations of the atypical alleles observed. Repeat codons are depicted: CAG glutamine codons as red boxes; CAA glutamine codons as green boxes; CCG proline codons as blue boxes; CCA proline codons as yellow boxes; and CCT proline codons as pink boxes. Disease-associated alleles with either zero, three or four downstream glutamine codons (Q^2^ = 0, 3 or 4), are indicated with a red upward triangle, green diamond, or green downward triangle, respectively. (For interpretation of the references to color in this figure legend, the reader is referred to the web version of this article.)

We analysed data from 203 participants from a TRACK-HD discovery cohort and 543 from an Enroll-HD replication cohort (Fig. 1, Table 2). DNA sequencing revealed that > 95% of *HTT* alleles comprised the typical glutamine-encoding repeat structure where there was a single copy of the CAACAG cassette (Q^2^ = 2) (Fig. 2, Table 1). We also detected three atypical glutamine-encoding repeat structures with either duplication (Q^2^ = 4, *n* = 13), or deletion (Q^2^ = 0, *n* = 7), of the glutamine-encoding CAACAG cassette, or an additional CAA between the pure CAG tract and the CAACAG cassette (Q^2^ = 3, *n* = 1) on the disease-associated chromosome (Fig. 2, Table 1). As expected, alleles with deletion of the CAACAG cassette (Q^2^ = 0, *n* = 7) yielded fragment length CAG estimates (Q^FL^) two repeats shorter than the pure CAG number (Q^1^). Surprisingly, all the disease-associated alleles with duplication of the CAACAG cassette (Q^2^ = 4, *n* = 13) yielded fragment length estimates (Q^FL^) only one repeat longer than the pure CAG (Q^1^), rather than the expected two (Figure S1A, appendix). These discrepancies presumably reflect mispriming of the PCR primer used for fragment length analysis that binds across the polymorphic region between the CAG and CCG tracts, yielding artefactual fragment length estimates (Q^FL^) that have no direct biological correlate (*i.e.* when Q^2^ = 4, Q^1^ ≠ Q^FL^ ≠ Q^T^ − 2) (Figure S1B–F, appendix).

**Fig. 3:**
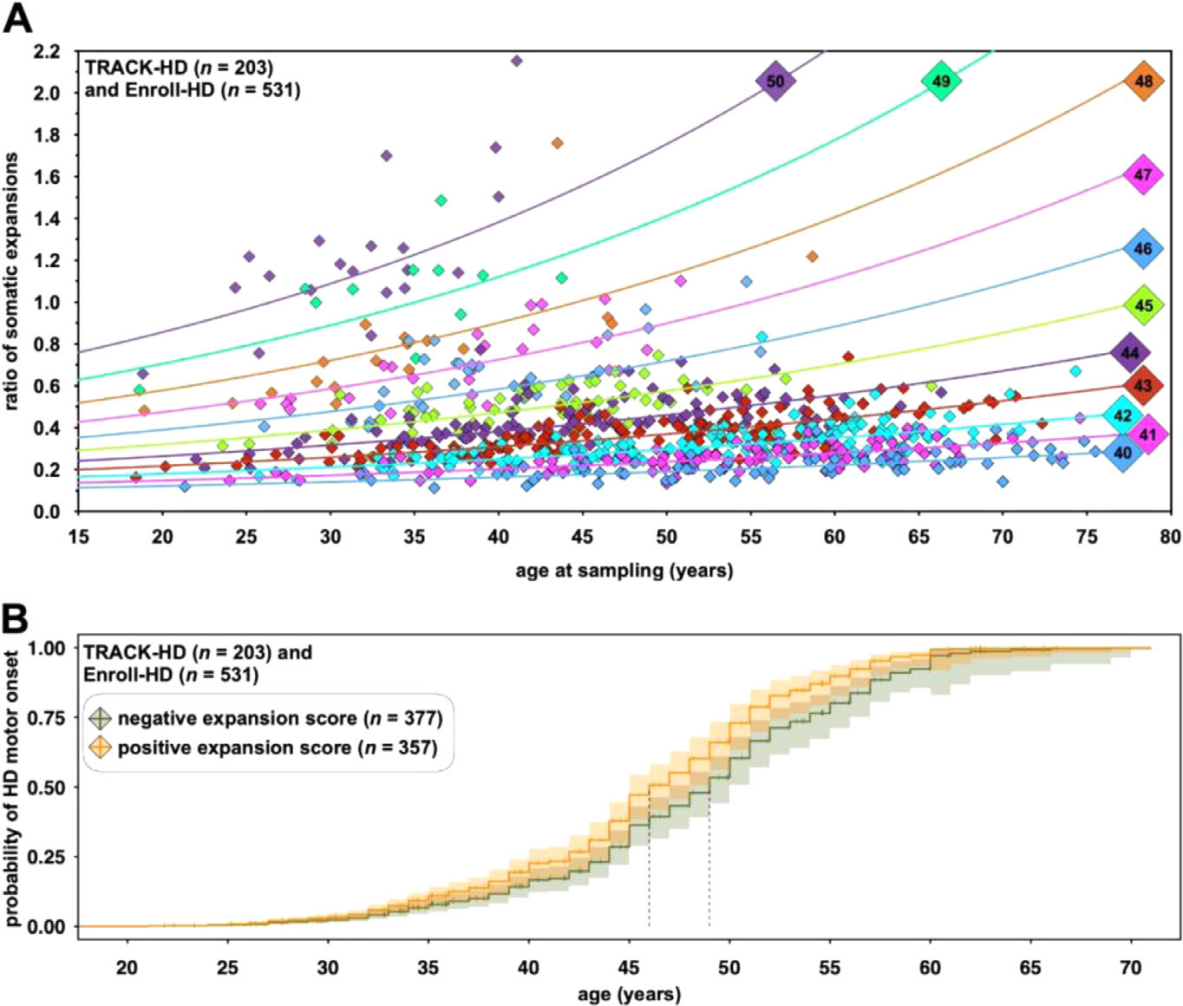
Somatic expansion in Huntington disease. **A)** The ratio of somatic expansions of the *HTT* exon one CAG repeat is allele length and age-dependent. The graph shows the ratio of somatic expansions $\left(\frac{\mathrm{number}\;\mathrm{of}\;\mathrm{somatic}\;\mathrm{expansion}\;\mathrm{products}}{\mathrm{number}\;\mathrm{of}\;\mathrm{progenitor}\;\mathrm{allele}\;\mathrm{products}}\right)$ in blood DNA plotted against the age at sampling for 746 participants in the TRACK-HD and Enroll-HD cohorts. Each point is colour coded with respect to the length of the inherited progenitor allele length (Q^1^). The scatterplot also shows the sex- and cohort-adjusted fitted regression lines for each pure CAG repeat length (Q^1^) derived from model SEQ^1^ which includes an interaction between age at sampling and pure CAG repeat length (Q^1^) (Table S2, appendix). **B)** Somatic expansion scores in blood DNA predict age at onset in Huntington disease. The graph shows adjusted time to event curves from a Cox proportional hazard model of HD motor onset according to the sign of the pure CAG somatic expansion score (determined using model SEQ^1^, Table S2, appendix). The solid lines represent the age-dependent probability of HD motor onset: orange for participants with positive expansion scores; and green for participants with negative expansion scores. The light shaded regions represent the 95% CI for the onset probability curves. A vertical tick mark on the curves indicates that a participant was censored at this time. Vertical dashed lines indicate median survival for each somatic expansion score category: individuals with a positive blood DNA somatic expansion score had a median age at onset = 46 years (95% CI 45 to 48); whilst individuals with a negative blood DNA somatic expansion score had a median age at onset = 49 years (95% CI 47 to 50). (For interpretation of the references to color in this figure legend, the reader is referred to the web version of this article.)

Although somatic expansions in HD patient brain tissue may be very large [4], the level of somatic mosaicism in peripheral tissues is low, but allele length-dependent and expansion-biased [7], [8], [24]. Here, sequencing of single input molecules of DNA revealed high levels of smaller PCR slippage products, but only a low proportion of products larger than the progenitor molecule (Figures S2 and S3, appendix). These data suggest the vast majority of expansion products detected in bulk DNA analyses of disease-associated alleles represent genuine somatic expansions (Figure S3C, appendix). The ratio of somatic expansions $\left(\frac{\mathrm{numb}\mathrm{er}\mathrm{of}\mathrm{soma}\mathrm{tic}\mathrm{expa}\mathrm{nsion}\mathrm{prod}\mathrm{ucts}}{\mathrm{numb}\mathrm{er}\mathrm{of}\mathrm{prog}\mathrm{enit}\mathrm{or}\mathrm{alle}\mathrm{le}\mathrm{prod}\mathrm{ucts}}\right)$ represents a quantitative measure of the degree of somatic expansion present in the blood DNA of an individual at a given point. Given the stabilising effects of repeat interruptions in other disorders [1], it was not surprising that the ratio of somatic expansions was best predicted by an interaction between age at sampling and the length of the pure CAG repeat (Q^1^), rather than the total encoded-glutamine length (Q^T^) (model SEQ^T^ *r*^2^_QT_ = 0·822, *p* < 2 × 10^−16^, model SEQ^1^ *r*^2^_Q1_ = 0·836, *p* < 2 × 10^−16^, Table S2, appendix, Fig. 3A) (difference in *r*^2^ = 0·014 (95% CI = 0·005 to 0·025)). Once corrected for pure CAG (Q^1^), the number of additional CAA/CAG codons (Q^2^) had no impact on the ratio of somatic expansions in blood DNA (model SEQ^1^Q^2^ *pFDR*_Q2=0_ = 0·36, *pFDR*_Q2=4_ = 0·36, p*FDR*_Q2=0*Age_ = 0·12, *pFDR*_Q2=4*Age_ = 0·51, Table S2, appendix) (permutation test for difference between models SEQ^1^Q^2^ and model SEQ^1^*, p* = 0·10). The ratio of somatic expansions corrected for age at sampling, the number of pure CAG repeats (Q^1^), sex and cohort (model SEQ^1^) represents an individual-specific somatic expansion score.

Motor AAO in HD is strongly predicted by the number of ‘CAG’ repeats inherited as estimated by PCR fragment length analysis (Q^FL^) [25]. However, the existence of atypical CAG/CAA structures allows us to ask whether HD clinical outcomes are best predicted by the length of the longest pure CAG tract (Q^1^), or by the total number of consecutive glutamines encoded by the CAG/CAA repeat (Q^T^). In addition, the availability of individual-specific somatic expansion scores, allows us to assess whether somatic expansions in blood are associated with variation in AAO. We thus performed multivariate time-to-event Cox proportional hazard regression analyses of variation in AAO (time to HD onset) dependent on ‘CAG’ length (pure CAG (Q^1^) or total encoded-glutamine length (Q^T^)), and the somatic expansion score. Pure CAG length (Q^1^) was a better predictor of AAO than total encoded-glutamine (Q^T^)(model AAOQ^T^ log-likelihood = −1899, model AAOQ^1^ log-likelihood = −1884, 95% CI of log-likelihood difference: 5·05 to 30·93, Table S3, appendix).

**Fig. 4:**
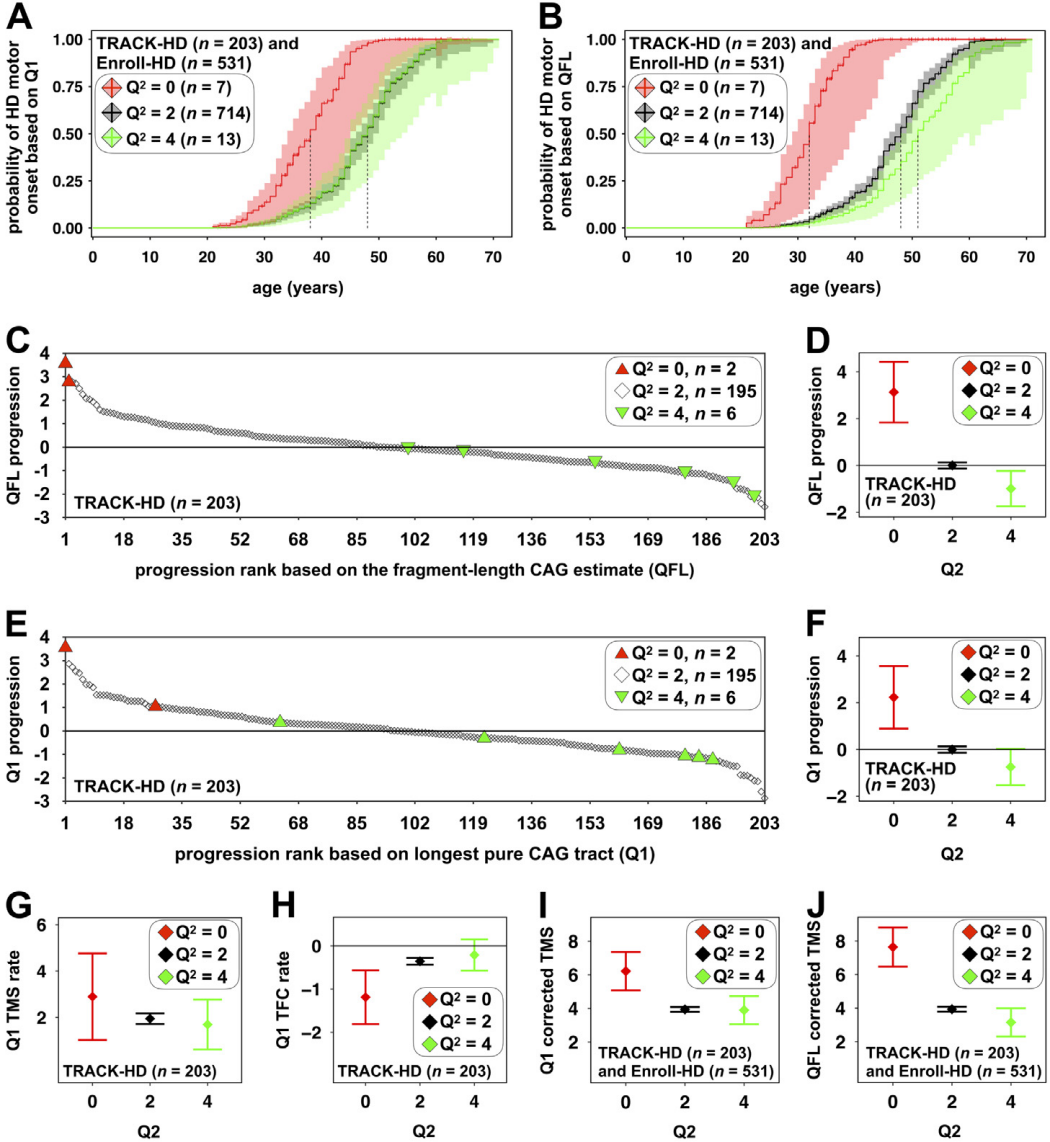
Associations between *HTT* exon one CAG repeat structures and HD clinical outcomes and biases associated with the prediction of HD clinical outcomes based on fragment length estimates of CAG**.** The adjusted probability curves of age at HD motor onset (**A/B**) show the probability of motor onset predicted on the basis of the pure CAG length (Q^1^, **A**) or the fragment length estimate of CAG (Q^FL^, **B**) for each number of additional glutamine codons genotype (Q^2^ = 0, red; Q^2^ = 2, black; Q^2^ = 4, green). The light shaded regions represent the 95% CI for the onset probability curves. A vertical tick mark on the curves indicate that a participant was censored at this time. Vertical dashed lines indicate median survival for each Q^2^ genotype. For the pure CAG (Q^1^) estimates (**A**): individuals Q^2^ = 0 had a median age at onset = 38 years (95% CI = 34 to 45); individuals Q^2^ = 2 had a median age at onset = 48 years (95% CI = 46 to 49); and individuals Q^2^ = 4 had a median age at onset = 48 years (95% CI = 45 to 54). For the CAG fragment length (Q^FL^) estimates (**B**): individuals Q^2^ = 0 had a median age at onset = 32 years (95% CI = 29 to 39); individuals Q^2^ = 2 had a median age at onset = 48 years (95% CI = 46 to 49); and individuals Q^2^ = 4 had a median age at onset = 51 years (95% CI = 48 to 59). The scatterplots (**C/E**) and the whisker plots (**D/F**) show the ranked TRACK-HD progression score predicted on the basis of the fragment length estimate of CAG (Q^FL^, **C/D**) or pure CAG (Q^1^, **E/F**) dependent on the number of additional glutamine codons (Q^2^). The progression scores are normally distributed around zero, with zero representing the population average disease progression. Negative values represent individuals with slower than average disease progression and positive values represent individuals with faster than average disease progression. The whisker plots show the mean (diamond) and 95% CI (whiskers) on the basis of the fragment length estimate of CAG (Q^FL^, **E**) or pure CAG length (Q^1^, **F**) dependent on the number of additional glutamine codons (Q^2^ = 0, red; Q^2^ = 2, black; Q^2^ = 4, green). The whisker plots (**G/H/I/J**) show the least-square means for the rate of change of TMS (TMS rate, **G**) and TFC (TFC rate**, H**) predicted on the basis of the pure CAG length (Q^1^), and TMS predicted on the basis of the pure CAG (Q^1^, **G**) or fragment length estimate of CAG (Q^FL^, **H**) for each number of additional glutamine codons (Q^2^) genotype (*i.e.* the means associated with each number of additional glutamine codons (Q^2^) genotype adjusted for all other variables in the linear regression).

Although pure CAG length (Q^1^) was a better predictor of AAO than total encoded glutamine length (Q^T^), under the assumption that the actual toxic element in cells is the polyglutamine protein [9], we might still expect that after correcting for the number of pure CAG repeats (Q^1^), fewer glutamine-encoding CAA/CAG codons (Q^2^) would increase AAO (Figure S4, appendix). Surprisingly, rather than increasing AAO relative to typical allele carriers (Q^2^ = 2), after correcting for pure CAG length (Q^1^), absence of the CAACAG cassette (Q^2^ = 0, *n* = 7) was associated with an earlier AAO of ∼10 years with a median age at HD onset = 38 years (95% CI = 34 to 45) relative to 48 years (95% CI = 46 to 49) for typical alleles (Q^2^ = 2) (*pFDR*_Q2=0_ = 7·3 × 10^−4^, model AAOQ^1^Q^2^, Table S3, appendix) (Fig. 4A). After correcting for pure CAG length, duplication of the CAACAG cassette (Q^2^ = 4, *n* = 13) was not associated with an earlier AAO with a median time to HD onset = 48 years (95% CI = 45 to 54) (*pFDR*_Q2=4_ = 0·95, model AAOQ^1^Q^2^, Table S3, appendix) (Fig. 4A).

The number of additional glutamine codons (Q^2^) was not associated with the length of the pure CAG tract (Q^1^) (*r*^2^ = 0·001, *p* = 0·17) (Figure S5, appendix). However, over the pure CAG (Q^1^) range of 40 to 50 repeats, there was a positive association between the number of additional glutamine codons (Q^2^) and the length of the total glutamine-encoding CAA/CAG repeat (Q^T^) (*r*^2^ = 0·034, *p* = 2 × 10^−7^) (Figure S5, appendix). Some association was inevitable because the total glutamine length (Q^T^) is sum of the number of pure CAGs (Q^1^) and the number of additional glutamine codons (Q^2^) (*i.e.* Q^T^ = Q^1^ + Q^2^). Furthermore, the pure CAG length selection criteria (40 ≤ Q^1^ ≤ 50) differentially eliminates some genotypes at either end of the distribution: individuals with Q^T^ = 40 and Q^2^ = 0 (and therefore Q^1^ = 40) were included, but individuals with Q^T^ = 40, and Q^2^ = 2 (and therefore Q^1^ = 38) were excluded; likewise, individuals with Q^T^ = 53, and Q^2^ = 4 (and therefore Q^1^ = 49) were included, but individuals with Q^T^ = 53 and Q^2^ = 2 (and therefore Q^1^ = 51) were excluded. This association might also have been compounded by an ascertainment bias whereby individuals with absence of the CAACAG cassette (Q^2^ = 0) may have an increased probability of receiving a diagnosis with a smaller total encoded glutamine repeat length (Q^T^).

In order to gauge the level of prognostic inaccuracy with the standard test, we revealed that relative to that predicted by fragment length analyses (Q^FL^) alone, absence of the CAACAG cassette (Q^2^ = 0, *n* = 7) was associated with an error in the predicted AAO of ∼16 years with a median age at HD onset = 32 years (95% CI = 29 to 39) relative to 48 years (95% CI = 46 to 49) for typical alleles (Q^2^ = 2) (*pFDR*_Q2=__0_ < 1·5 × 10^−5^, model AAOQ^FL^Q^2^, Table S3, appendix) (Fig. 4B). Conversely, duplication of the CAACAG cassette (Q^2^ = 4, *n* = 13) appeared to be associated with an ∼3-year later predicted AAO with a median time to HD onset = 51 years (95% CI = 48 to 59) (*pFDR*_Q2=4_ = 0·11, model AAOQ^FL^Q^2^, Table S3, appendix) (Fig. 4B).

If the accumulation of somatic expansions contributes toward disease development, then individual-specific variation in the rate of somatic expansion should be associated with variation in HD outcomes. As hypothesised, variation in AAO, corrected for the number of pure CAG repeats (Q^1^), was correlated with the individual-specific somatic expansion scores in blood DNA (model AAOQ^1^Q^2^, hazard ratio = 3·05, 95% CI = 1·94 to 4·80, *pFDR_SE_* = 2·8 × 10^−6^). Individuals with a positive blood DNA somatic expansion score (median age at onset = 46, 95% CI 45 to 48) had a three year earlier median age at onset than individuals with a negative blood DNA somatic expansion score (median age at onset = 49, 95% CI 47 to 50) (Fig. 3B) *i.e.* individuals with more somatic expansions in blood DNA than average, had an earlier age at onset than average.

Although AAO is of particular interest to patients, it can be difficult to assess, and it is unlikely to be a clinical trial outcome in the near term. However, careful clinical testing and imaging can reveal differences in the premanifest HD population many years before the onset of overt motor symptoms [2], [26], [27]. The TRACK-HD study was established specifically to determine the utility of multiple outcomes to assess disease progression in the late premanifest and early HD population as representative of likely trial cohorts. Previously, Hensman Moss et al., used principal component analysis to combine 24 clinical variables, encompassing brain volume, cognition and motor performance, corrected for age and ‘CAG’ repeat length (Q^FL^) to derive a progression score in TRACK-HD (appendix) [14]. This progression score also proved very sensitive for detecting genetic modifiers of HD [14]. Here, the two individuals with deletion of the CAACAG cassette (Q^2^ = 0) in their disease-associated allele ranked as the two fastest progressing TRACK-HD participants using the previously determined progression score based on their measured ‘CAG’ length (Q^FL^) (Fig. 4C). Conversely, 5/6 carriers of the CAACAG cassette duplication (Q^2^ = 4) had a lower disease progression score than average (Fig. 4C). Regression analysis confirmed that relative to that predicted by the fragment length estimate of CAG (Q^FL^), absence of the CAACAG cassette (Q^2^ = 0, *n* = 2) was associated with a 3·1 unit (95% CI = 1·8 to 4·4) increase in the progression score (*pFDR*_Q2=0_ = 1 × 10^−4^); and duplication of the CAACAG cassette (Q^2^ = 4, *n* = 6) was associated with an apparent −0·99 unit (95% CI = −1·76 to −0·22) decrease in the progression score (*pFDR*_Q2=4_ = 0·028) (Fig. 4D, model ProgQ^FL^, Table S4, appendix). When the disease progression score was re-calculated using the length of the pure CAG repeat (Q^1^), the protective effect of the number of additional glutamine codons (Q^2^) was reduced, but remained apparent: absence of the CAACAG cassette (Q^2^ = 0, *n* = 2) was associated with a 2·2 unit (95% CI = 0·9 to 3·6, *pFDR*_Q2=0_ = 0·014) increase in the progression score; and duplication of the CAACAG cassette (Q^2^ = 4, *n* = 6) was nominally associated with a −0·74 unit (95% CI = −1·54 to 0·05, *pFDR*_Q2=4_ = 0·110) decrease in the progression score (Fig. 4E/F, model ProgQ^1^, Table S4, appendix). Individuals with higher somatic expansion scores were also revealed as having higher TRACK-HD progression scores: a one-unit increase in the blood DNA somatic expansion score was associated with a 0·98 unit increase in the pure CAG (Q^1^) progression score (95% CI = 0·24 to 1·72, *pFDR*_SEQ1_ = 0·028, model ProgQ^1^, Table S4, appendix).

Sensitive as the TRACK-HD progression score is as a detector of genetic modifiers, it is hard to relate to individual features of the disease. Thus, in order to increase the interpretability of the overall effects on progression, we determined the effect sizes of the sequence of the CAG/CAA repeat and the somatic expansion score on the rate of change of total motor score (TMS) and the total functional capacity (TFC) score. TMS and TFC score are components of the TRACK-HD progression score that are widely used, sensitive clinical outcome measures of HD (note that a higher TMS, and a lower TFC, is a worse outcome) [15]. These analyses revealed that after correcting for baseline TMS, age and the length of the pure CAG tract (Q^1^), absence of the CAACAG cassette (Q^2^ = 0, *n* = 2) was nominally associated with a faster rate of increase of TMS of ∼1 units year^−1^ (95% CI = −0·9 to 2·8, *pFDR*_Q2=0_ = 0.328) and a faster rate of decrease of TFC of ∼-0·8 units year^−1^ (95% CI = −1·5 to −0·2, *pFDR*_Q2=0_ = 0.038), whilst presence of the CAACAG duplication (Q^2^ = 4, *n* = 6) appeared to be associated with a slower rate of increase in TMS of ∼-0·3 units year^−1^ (95% CI = −1·4 to 0·9, *pFDR*_Q2=4_ = 0·66) and a slower rate of decrease in TFC of ∼0·1 units year^−1^ (95% CI = −0·2 to 0·5, *pFDR*_Q2=4_ = 0·53) (Table S5, models TMSrateQ^1^Q^2^ and TFCrateQ^1^Q^2^, appendix) (Fig. 4G/H). A one-unit increase in the blood DNA somatic expansion score was nominally associated with a 1·7 unit year^−1^ (95% CI = 0·7 to 2·8, *pFDR*_SEQ1_ = 0·002) acceleration in the rate of increase of TMS and with a −0·12 unit year^−1^ (95% CI = −0·46 to 0·23, *pFDR*_SEQ1_ = 0·561) acceleration in the rate of decrease of TFC (Table S5, models TMSrateQ^1^Q^2^ and TFCrateQ^1^Q^2^, appendix).

TMS was also available for Enroll-HD, thus in order to assess the generalisability of the TRACK-HD data, we also determined if baseline TMS was associated with the sequence of the CAG/CAA repeat and the somatic expansion score. In the combined cohorts, pure CAG length (Q^1^) was a better predictor of age-adjusted baseline TMS than total encoded-glutamine (Q^T^) (model TMSQ^T^ *r*^2^ = 0·558, model TMSQ^1^ *r*^2^ = 0·578, Table S6, appendix) (difference in *r*^2^ = 0·020 (95% CI = 0·0098 to 0·036)). Higher TMS is a worse outcome and these analyses revealed that after correcting the age-adjusted TMS for pure CAG (Q^1^), absence of the CAACAG cassette (Q^2^ = 0, *n* = 7) was associated with an increase in baseline TMS of ∼2·3 units with a mean baseline TMS = 6·2 units (95% CI = 5·1 to 7·4) relative to 3·9 units (95% CI = 3·8 to 4·1) for typical alleles (Q^2^ = 2) (*pFDR*_Q2=0_ = 1·9 × 10^−4^, model TMSQ^1^Q^2^, Table S6, appendix) (Fig. 4I). Duplication of the CAACAG cassette (Q^2^ = 4, *n* = 13) was not associated with a lower TMS = 3·9 units (95% CI = 3·1 to 4·7) (p*FDR*_Q2=4_ = 0·92, model TMSQ^1^Q^2^, Table S6, appendix) (Fig. 4I). If instead, the age-adjusted TMS was corrected for the fragment length estimate of CAG (Q^FL^), absence of the CAACAG cassette (Q^2^ = 0, *n* = 7) was associated with an increase in baseline TMS of ∼3·7 units with a mean baseline TMS = 7·6 units (95% CI = 6·5 to 8·8) relative to 3·9 units (95% CI = 3·8 to 4·1) for typical alleles (Q^2^ = 2) (*pFDR*_Q2=0_ = 1·9 × 10^−5^, model TMSQ^FL^Q^2^, Table S6, appendix) (Fig. 4J). Duplication of the CAACAG cassette (Q^2^ = 4, *n* = 13) appeared to be associated with a 0·8 unit lower TMS with a mean TMS = 3·1 years (95% CI = 2·3 to 4·0) (*pFDR*_Q2=4_ = 0·08, model TMSQ^FL^Q^2^, Table S6, appendix) (Fig. 4J). Individuals with higher somatic expansion scores were also revealed as having higher TMS (*pFDR*_SEQ1_ = 2·5 × 10^−4^, model TMSQ^1^Q^2^, Table S6, appendix).

To determine whether DNA repair gene variants found to modify HD outcomes [10], [14] might act by modifying somatic expansion, we genotyped 28 candidate SNPs in the discovery TRACK-HD cohort (Table 3, Table S7, appendix). Although five SNPs were nominally significantly (*p* < 0·05) associated with the somatic expansion scores, only three SNPs at the *MLH3* and *MTMR10/FAN1* loci remained significant after correction for multiple testing (*pFDR*_rs175080_ = 0·034 in *MLH3*, and *pFDR*_rs2140734_ and *pFDR*_rs3512_ = 0·034 at the *MTMR10/FAN1* locus, Table 3, Table S7, appendix). To replicate these effects in Enroll-HD, we selected six candidate SNPs that approached statistical significance in the TRACK-HD cohort (*p* *<* ∼0·1) and that could be genotyped using KASP assays. We revealed associations with somatic expansion scores for variants in *MLH3* (*pFDR*_rs175080_* = *0·026), *FAN1* (*pFDR_rs3512_ = *4·0 × 10^−4^) and *MLH1* (*pFDR*rs1799977 = 0·026) in the Enroll-HD cohort (Table 3). Signals for *MLH3* (*pFDR*_rs175080_* = *8·0 × 10^−4^), *FAN1* (*pFDR_rs3512_ = *4·8 × 10^−6^) and *MLH1* (*pFDR*rs1799977 = 0·004) were further amplified in a meta-analysis of the TRACK-HD and Enroll-HD association tests, which also brought a variant in *MSH3* below the nominal multiple testing significance threshold (*pFDR*rs1382539 = 0·009, Table 3, Table S7, appendix).

**Table 3:** Genetic modifiers of blood DNA somatic expansion scores of the *HTT* CAG repeat. Shown is the genetic association data for the six SNPs genotyped in TRACK-HD and replicated in Enroll-HD. Additional details of all the SNPs analysed are provided in Table S7 (appendix). SNPs are ordered by decreasing *p*-value of their association with the somatic expansion score in the TRACK-HD cohort. Chr: chromosome. A1: minor allele. *β*: regression coefficient. *p*: unadjusted *p*-value. *p FDR: p*-value adjusted for multiple testing using the Benjamini-Hochberg false discovery rate correction. *Note that in a preliminary analysis using a slightly larger TRACK-HD cohort including four participants with 39 pure CAG repeats (Q^1^ = 39) and six non-Caucasians, the association between somatic expansion score and rs20579 in *LIG1* was *p* = 0·072, and rs20579 was thus selected for replication in Enroll-HD.

SNP ID	Chr	Gene	A1	A2	TRACK-HD			Enroll-HD			Meta-analysis
					*β*	*p*	*p FDR*	*β*	*p*	*p FDR*	*p FDR*
rs3512	15	*FAN1*	C	G	0·060	0·003	0·034	0·050	6·7 × 10^−5^	4·0 × 10^−4^	4·8 × 10^−6^
rs175080	14	*MLH3*	A	G	−0·053	0·004	0·034	−0·029	0·013	0·026	8·0 × 10^−4^
rs147804330	2	*RP11-481J13.1*	A	G	−0·107	0·010	0·073	0·003	0·912	0·912	0·246
rs1382539	5	*MSH3*	A	G	−0·045	0·018	0·101	−0·023	0·077	0·116	0·009
rs1799977	3	*MLH1*	G	A	−0·032	0·093	0·314	−0·034	0·009	0·026	0·004
rs20579	19	*LIG1*	A	G	−0·042	0·112*	0·314	−0·002	0·908	0·912	0·351

## 4 Discussion

We used high-throughput ultra-deep sequencing to determine the precise structure of the *HTT* exon one HD-causing repeat, and simultaneously quantify somatic expansions, in two large cohorts of carriers of HD-associated alleles. We established that, although none of the DNA sequence variants detected changed the amino acid sequence of the encoded polyglutamine repeat (beyond its length), the precise DNA sequence of the *HTT* polyglutamine-encoding repeat was associated with HD outcomes. Notably, HD clinical outcomes were best predicted by the number of pure CAG repeats (Q^1^) rather than the total number of consecutive glutamine-encoding CAG/CAA repeats (Q^T^) on the disease chromosome. The finding that duplication of the CAACAG cassette (Q^2^ = 4) appeared to be associated with a later AAO compared to that predicted using standard fragment length analysis (Q^FL^) is consistent with previous reports of a protective *HTT* haplotype present in a subset of Danish HD families [28]. Although not originally considered as a likely explanation, the protective HD haplotype was revealed to carry the duplication of the CAACAG cassette (Q^2^ = 4) [28] on a (CCGCCA)_1_(CCG)_7_(CCT)_3_ repeat haplotype [29], identical to all 37 CAACAG cassette duplication alleles we detected (Table 1) and in three previous reports (Table S1, appendix). The effects of absence or duplication of the CAACAG cassette with predicted age at onset have also been replicated in two very recent reports published while this paper was in review [30,31]. In the largest GWAS of modifiers of HD age at motor onset yet reported, the Genetic Modifiers of HD team identified a rare protective *HTT* haplotype, which, based on the fragment length estimate of ‘CAG’ (Q^FL^), delayed motor onset by ∼ 4 to 5 years and was associated with the CAACAG cassette duplication (Q^2^ = 4) and (CCGCCA)_1_(CCG)_7_(CCT)_3_ repeat haplotype [30]. Wright et al., also associated the CAACAG cassette duplication (Q^2^ = 4) with delayed motor onset of ∼ 4 years based on the fragment length estimate of ‘CAG’ (Q^FL^) [31]. The Genetic Modifiers of HD team also detected an even rarer deleterious *HTT* haplotype, which, based on the fragment length estimate of ‘CAG’, accelerated motor onset by ∼ 9 to 10 years, and was associated with loss of the CAACAG cassette (Q^2^ = 0) and a (CCGCCA)_0_(CCG)_12_(CCT)_2_ repeat haplotype [30]. Similarly, Wright et al., associated the CAACAG cassette deletion (Q^2^ = 0) with an accelerated age at onset of ∼29 years in carriers of reduced penetrance alleles (36 to 39 ‘CAG’ repeats), and ∼ 13 years in carriers of full mutations (40 to 50 ‘CAG’ repeats), relative to the age at onset predicted by the fragment length estimate of ‘CAG’ (Q^FL^) [31]. Interestingly, Wright et al., observed the loss of the CAACAG cassette (Q^2^ = 0) on either a (CCGCCA)_0_(CCG)_10_, (CCGCCA)_0_(CCG)_7_ or (CCGCCA)_1_(CCG)_10_ repeat haplotype [31]. Notably, we have not observed the (CAACAG)_0_(CCGCCA)_0_(CCG)_12_, (CAACAG)_0_(CCGCCA)_0_(CCG)_10_, (CAACAG)_0_(CCGCCA)_0_(CCG)_7_ or (CAACAG)_0_(CCGCCA)_1_(CCG)_10_ disease-associated repeat haplotypes in our data. Nonetheless, we observed a similar effect size combining data from (CAACAG)_0_ (Q^2^ = 0) disease associated alleles observed on three different (CCGCCA)_P1_(CCG)_P2_ repeat haplotypes (Table 1). These data strongly suggest multiple origins for the CAACAG cassette deletion alleles (Q^2^ = 0), and, that the size of the modifying effect of loss of the CAACAG cassette (Q^2^ = 0) is overestimated due to the mis-sizing the pure CAG repeat tract (Q^1^) by fragment length analysis and is not mediated by some other linked modifier. The finding that fragment length analysis of *HTT* CAG length is inaccurate in subjects with atypical alleles has implications for clinical trials with CAG repeat length inclusion criteria, potentially modifying the predicted AAO by from approximately −16 to +3 years. These findings also have implications at the lower end of the disease-associated CAG range where discrepancies of +/- one to two CAG repeats could move an allele between the non-pathogenic and low penetrance ranges, or between the low and fully penetrant ranges. As atypical disease-associated *HTT* alleles are relatively rare, additional larger and more detailed studies will be needed to better predict absolute effect sizes on HD outcomes for each specific structure. Nonetheless, these findings have implications for genetic diagnosis and counselling, and support a move toward sequence-based diagnosis and genetic stratification in HD clinical trials.

A possible explanation for the greater predictive power of pure CAG (Q^1^) as opposed to total encoded-glutamine length (Q^T^), is that somatic expansion of the CAG repeat is a driver of disease pathology. This interpretation is supported by the observation that somatic expansion scores in blood DNA were, as expected [1], best predicted by the length of the pure CAG repeat (Q^1^). Moreover, we were also able to determine that individuals with higher somatic expansion scores in blood DNA, had worse clinical outcomes than average. In further support of a direct role for somatic expansions in mediating pathology in HD, we showed that some DNA repair gene variants revealed by GWAS to associate with HD outcomes [10], [14], [30], also associate with somatic expansion scores in blood DNA, consistent with the role of these genes in generating expansions in model systems [1], [32]. However, the effect for *MSH3* was modest and we were not able to detect associations with *LIG1, PMS1*, and *PMS2*, possibly as a limitation of the sample size. A role for somatic expansion in driving brain pathology is supported by the finding that a greater frequency of large expansions in the cortex of end-stage patients is associated with extreme early AAO in HD relative to that predicted using fragment length analysis (Q^FL^) [33]. Thus, unless somatic expansions in blood DNA are directly impacting on primarily neurological disease outcomes, then the associations between somatic expansion scores in blood and disease outcomes, suggest that the dynamics of *HTT* somatic expansion measured in blood broadly parallel those in the brain (Figure S6, appendix). Additional data are required to evaluate directly the degree of comparability between somatic expansion dynamics in blood and brain. A role for pure CAG length in driving pathology via somatic expansion may also apply to other disorders associated with the expansion of polyglutamine-encoding repeats, such as SCA1, in which repeat interruptions also appear to modify disease outcomes [34].

It is also possible that the greater predictive power of the pure CAG (Q^1^) relative to the total number of consecutive encoded glutamines (Q^T^) is not driven by somatic expansion, but other mechanisms. These could include: i) there is an additional effect of linked variants, on, for instance, *HTT* transcription [29]; ii) there are sequence-dependent effects on the *HTT* transcript, that might modify folding [35], splicing [36], or canonical translation [37]; iii) there are sequence-dependent effects on RAN translation, possibly generating toxic polyalanine, polyserine, polyleucine or polycysteine proteins from the sense or antisense transcript [38]; or, iv) the toxic element is not the encoded protein, but toxic RNA [39].

Somatic expansion ratios were best predicted by the length of the pure CAG repeat (Q^1^) and in our data there was no additional stabilising effect of the number of copies of the CAACAG cassette. Indeed, after correcting for pure CAG (Q^1^), there was a slight trend for additional copies of the CAACAG cassette to be associated with greater somatic expansion ratios. In contrast, Wright et al., have recently reported that the number of copies of the CAACAG cassette does directly modify somatic mutational dynamics in blood DNA [31]. However, it should be noted that their somatic expansion ratios have been corrected for the fragment length estimate of ‘CAG’, and not the number of pure CAG repeats (Wright and Hayden, personal communication) [31]. In addition, most of their data derive from the analysis of reduced penetrance alleles (36 to 39 ‘CAG’ repeats). Larger studies will be required to further evaluate possible interactions between pure CAG length (Q^1^) and the number of copies of the CAACAG cassette on somatic expansions in blood DNA.

Interestingly, correcting for pure CAG (Q^1^) does not appear to completely eliminate the disease-moderating effect of the number of additional glutamine codons (Q^2^). This effect appeared to be more pronounced for the deletion of the CAACAG cassette (Q^2^ = 0), than the duplication of the CAACAG cassette (Q^2^ = 4). Nonetheless, if the polyglutamine protein is the toxic entity in cells, correcting for pure CAG would be expected to invert the disease-modifying effect of the number of copies of the CAACAG cassette, since alleles with more CAACAG cassettes will translate to proteins containing a greater total number of toxic glutamines (Figure S4, appendix). In addition to a contribution of the alternative mechanisms outlined above, explanations for this could also include: i) the number of additional CAA/CAG codons has an additional stabilising effect on somatic expansions in the brain, not reflected in somatic expansion scores in blood; or ii) somatically acquired disease-relevant expansions in brain are so large, that +/− two glutamines are not detectable/biologically relevant.

Our data, the GWAS results [10], [14], [30], and data on somatic expansions in HD brains [[4], [5], [6],^33^], converge on somatic expansion as a potential driver of disease pathology in HD. For the variants in *MLH1* and *MSH3*, the directions of the effect in independent association studies [10], [14], [30] are consistent, *i.e.* the alleles we have associated with higher somatic expansion scores in blood DNA are associated with an earlier AAO [10] or more rapid disease progression [14]. However, for the variants in *MLH3* and *FAN1*, the effect directions are not consistent, *i.e.* alleles associated with higher somatic expansion scores in blood DNA are associated with later AAO^10^ or slower disease progression [14]. Notably, both the *MLH3* and *FAN1* SNPs are revealed as bidirectional tissue-specific expression quantitative trait loci in the human Genotype-Tissue Expression database [40] (Table S8, appendix) (*i.e.* the same allele is associated with increased mRNA levels in some tissues and lower in others). Thus, the causative variant could mediate opposite effects on *FAN1*/*MLH3* expression and somatic expansion in the haematopoietic stem cells from which circulating white blood cells are derived, relative to the critical regions of the brain affected in HD (Figure S6, appendix). Tissue-specific effects of genetic modifiers might also explain the relatively modest associations between disease outcomes and blood DNA somatic expansions scores, which, given the associations of DNA repair gene SNPs with disease outcomes [10,14,30], might reasonably have been expected to be more dramatic. Although confounded by the complexity of the underlying biology, our data nonetheless reveal somatic expansion in blood as a potential peripheral biomarker of disease-relevant modifiers of somatic expansion for genetic studies and drug trials. To this end, longitudinal analyses of somatic expansion will be a key next step, in addition to the development of an assay that quantifies somatic contractions.

By associating genetic modifiers of HD outcomes to individual-specific somatic expansion scores, these data support somatic expansion as a factor in HD pathogenesis and further highlight FAN1, MSH3, MLH3 and MLH1 and other components of the expansion pathway and/or the sequence integrity of the CAG repeat tract as potential therapeutic targets in this disorder. Although null alleles in some DNA mismatch repair genes, such as *MLH1* and *MSH2* [41]*,* have been associated with hereditary non-polyposis colon cancer, the common DNA repair gene polymorphisms analysed here are not associated with an overt cancer predisposition phenotype, yet are nonetheless associated with positive impacts on HD outcomes. This suggests that pharmacological interventions that achieved a similar impact on DNA repair activity might be therapeutically beneficial and safe in HD and related repeat expansion disorders.

## Role of the funding source

The funders of this study had no role in study design, data collection, data analysis, data interpretation, or writing of the report. The corresponding authors had full access to all the data in the study and had final responsibility for the decision to submit for publication.

## CRediT authorship contribution statement

**Marc Ciosi:** Conceptualization, Formal analysis, Investigation, Methodology, Validation, Visualization, Writing - original draft, Writing - review & editing. **Alastair Maxwell:** Formal analysis, Investigation, Methodology, Software, Writing - review & editing. **Sarah A. Cumming:** Investigation, Methodology, Writing - review & editing. **Davina J. Hensman Moss:** Conceptualization, Resources, Writing - review & editing. **Asma M. Alshammari:** Methodology, Writing - review & editing. **Michael D. Flower:** Resources, Writing - review & editing. **Alexandra Durr:** Resources, Writing - review & editing. **Blair R. Leavitt:** Resources, Writing - review & editing. **Raymund A.C. Roos:** Resources, Writing - review & editing. **Peter Holmans:** Supervision, Writing - review & editing. **Lesley Jones:** Supervision, Writing - review & editing. **Douglas R. Langbehn:** Formal analysis, Supervision, Writing - review & editing. **Seung Kwak:** Resources, Supervision, Writing - review & editing. **Sarah J. Tabrizi:** Resources, Supervision, Writing - review & editing. **Darren G. Monckton:** Formal analysis, Funding acquisition, Supervision, Validation, Visualization, Writing - original draft, Writing - review & editing.

## Declaration of Competing Interest

MC, AM, SAC, DJHM, AMA, MDF, AD, BRL, PH, and LJ have nothing to disclose. RACR reports personal fees from UniQure, outside the submitted work. DRL reports personal fees from Roche Pharmaceutical, Voyager, Teva Pharmaceutical, Wave Life Sciences, Takeda Pharmaceutical Company and Axon Advisors, and other from CHDI, outside the submitted work. SK reports and is employed by CHDI Management, Inc., as an advisor to the CHDI Foundation. SJT reports personal fees from Alnylam Pharmaceuticals, DDF Discovery, F. Hoffmann-La Roche, Genentech, GSK, Heptares Therapeutics, Takeda Pharmaceutical Company, Teva Pharmaceuticals, Triplet Therapeutics, UCB Pharma and Vertex Pharmaceuticals, outside the submitted work. DGM reports other from CHDI Foundation, during the conduct of the study and personal fees from Vertex Pharmaceuticals, LoQus23 Therapeutics and Triplet Therapeutics, outside the submitted work.

## Data Availability

The TRACK-HD and Enroll-HD clinical data that support the findings of this study are available for approved studies from CHDI (https://chdifoundation.org) and https://www.enroll-hd.org/for-researchers/access-data/ respectively, but restrictions apply to the availability of these data, which were used under license for the current study, and so are not publicly available. The linked novel data generated here are available from the authors upon request (darren.monckton@glasgow.ac.uk), subject to approval from TRACK-HD and/or Enroll-HD as appropriate.

## Appendix Supplementary materials

Image, application 1
