# Supplementary material for: A genetic association study of glutamine-encoding DNA sequence structures, somatic CAG expansion, and DNA repair gene variants, with Huntington disease clinical outcomes
Source: eBioMedicine. 2019 Oct 10;48:568–80. doi: 10.1016/j.ebiom.2019.09.020 (PMC6838430; doi:10.1016/j.ebiom.2019.09.020)
Supplement: Supplementary file 1 [file mmc1.pdf]

A genetic association study of glutamine-encoding DNA sequence structures, somatic CAG expansion, and DNA repair gene variants, with Huntington disease clinical outcomes

Marc Ciosi, Alastair Maxwell, Sarah A. Cumming, Davina J. Hensman Moss, Asma M. Alshammari, Michael D. Flower, Alexandra Durr, Blair R. Leavitt, Raymund A. Roos, the TRACK-HD team, the Enroll-HD team, Peter Holmans, Lesley Jones, Douglas R. Langbehn, Seung Kwak, Sarah J. Tabrizi and Darren G. Monckton

EBioMedicine 2019

Supplementary material: table of contents

Section	Subsection	Pages
**I. Supplementary text**	I.1. Supplementary methods	2 to 6
**II. Supplementary tables**	Tables are listed in the order in which they are referred to in the main and then the supplementary text	7 to 19
	Table S1: Literature review of atypical *HTT* structures and their association with Huntington disease (HD) clinical outcomes and genetic instability	7
	Table S2: Linear regression models of relationships between allele structures and somatic expansions in HD	8 to 9
	Table S3: Cox regression models of relationships between allele length, sequence structure and somatic expansion scores with age at onset of HD motor symptoms	10
	Table S4: Linear regression models of relationships between sequence structure and somatic expansions scores with disease progression in HD	11
	Table S5: Linear regression models of relationships between sequence structure and somatic expansions scores with the rate of change of TMS and TFC in HD	12
	Table S6: Linear regression models of the relationship between sequence structure and somatic expansions scores with TMS in HD	13 to 14
	Table S7: Genetic associations between candidate SNPs and the somatic expansion scores of the *HTT* CAG repeat	15
	Table S8: Human tissue-specific expression quantitative trait data for DNA repair gene SNPs	16 to 19
**III. Supplementary figures**	Figures are listed in the order in which they are referred to in the main and then the supplementary text	20 to 25
	Figure S1: Comparison of CAG repeat lengths determined by deep-sequencing (Q^1) and estimated by fragment-length analyses (Q^{FL})	20
	Figures S2/3: Read depth distributions for CAG repeat length support a somatic origin for repeat length gains	21 to 22
	Figure S4: Relative toxicities for alleles dependent either on total glutamine or pure CAG length	23
	Figure S5: Association of pure CAG repeat length (Q^1), fragment length estimated CAG (Q^{FL}) and total encoded-glutamine length (Q^T) with the number of additional glutamine codons (Q^2) on disease-associated chromosomes	24
	Figure S6: Blood and brain dynamics of CAG repeats dependent on CAG structures and SNP genotypes	25
**IV. References for supplementary material**	IV.1. References	26 to 27
**V. Investigator lists**	V.1. TRACK-HD investigator list	28 to 29
	V.2. Enroll-HD investigator list	29 to 35

I. Supplementary text
I.1. Supplementary Methods
I.1.1. HD cohorts

I.1.1.1. TRACK-HD

Track-HD was a prospective, observational study that collected deep clinical data, including imaging, quantitative motor and cognitive assessments, from adults with early HD, premanifest HD and controls. Comprehensive details of entry inclusion and exclusion criteria and group definitions are available in the online supplement to Tabrizi *et al.*[1] Briefly, multivariate clinical, neuropsychological, psychiatric, and brain-imaging data were collected annually at four visits spanning approximately 36 months of follow-up from 2008 to 2011. Baseline data were collected on 120 premanifest HD, 123 early HD, and 123 control participants, recruited evenly from study sites in London (UK), Paris (France), Leiden (Netherlands), and Vancouver (BC, Canada).[1] Manifest HD subjects were required to demonstrate motor abnormalities that were unequivocal signs of HD, as evidenced by total motor scores (TMS) over five, and diagnostic confidence level of four on the Unified Huntington's Disease Rating Scale (UHDRS).[2] Furthermore, their functional level measured by the UHDRS total functional capacity scale (TFC) was seven or higher. Thus, these subjects were in the early clinical stages of manifest HD. Premanifest HD mutation carriers were required to have a burden of pathology score (age x [CAG – 36·5))[3] greater than 250. This criterion was adopted to ensure that potential participants with very little probability of showing any progression toward diagnosed HD were excluded. Further, the pre-HD participants were required to have a screening baseline UHDRS TMS of five or lower diagnostic confidence level less than four, which is widely used as the threshold for considering a person "diagnosed" with clinically significant HD. Potential subjects were excluded if they were less than 18 years of age, if their burden of pathology score was less than 250, if they were unable to give informed consent, if they were unable to tolerate MRI scanning or bio-sample collection, if they had a history of major psychiatric illness (*e.g.* schizophrenia) or history of significant head trauma, or if they were currently participating in a clinical trial for any experimental HD treatment.

The present study initially considered 218 adult participants from TRACK-HD with premanifest or early clinical HD who had at least one follow-up visit that included successful brain MRI measurement (disease progression, the target outcome, could not be scored on 25 other participants).[4] Fifteen TRACK-HD participants were excluded: one from a twin pair; one whom, in addition to a non-disease associated allele, presented with a bimodal read count distribution with pure CAG (Q^1) modes at 43 and 45 CAG repeats; three participants with a pure CAG tract (Q^1) modal allele > 50 CAG in who, because of the combination of PCR slippage and high levels of somatic expansion, the inherited progenitor allele could not be unambiguously identified; four participants with a pure CAG < 40; and six non-Caucasians. The data presented here thus correspond to 203 out of the 218 TRACK-HD participants, comprising 105 premanifest, and 98 manifest, for motor symptoms. TRACK-HD DNA repair gene SNP genotypes were taken from Hensman Moss *et al.*[4] TRACK-HD fragment length analysis genotypes (Q^{FL}) were generated by BioRep (http://www.biorep.it/en) using standard procedures.[5-7]

I.1.1.2. Enroll-HD

Enroll-HD is a global clinical research platform designed to facilitate clinical research in Huntington disease.[8] Full details of the HD eligibility and exclusion criteria and clinical assessments are available from https://www.enroll-hd.org/for-researchers/technical-support/. Briefly, Enroll-HD aims to recruit subjects 18 years or older who are HD gene expansion mutation carriers (CAG > 35) independent of the clinical manifestation or the stage of HD, and controls who do not carry the HD expansion mutation and who comprise the comparator study population. For individuals under the age of 18 years, those with clinically diagnosed features of HD in the setting of a confirmatory family history or a positive genetic test result were also eligible. Individuals who do not meet the criteria above or with choreic movement disorders with a negative test for HD expansion mutation are excluded. Enroll-HD study sites recruit participants through clinical visits, family referral, outreach through the many lay associations, and genetic counselling centres. Core data sets are collected annually on all research participants as part of this multi-centre longitudinal observational study of HD. Data are monitored for quality and accuracy using a risk-based monitoring approach. Enroll-HD remains open to recruitment and, as of 2nd January 2019, had 17,886 active participants recruited via 172 centres in 19 countries.

With the aim of replicating the findings of the initial analysis on TRACK-HD data, we collected data from a replication cohort of 615 participants from Enroll-HD carrying a disease-associated allele ($Q^1 > 35$). To perform the replication study with similar characteristics to the initial TRACK-HD analysis, 72 Enroll-HD participants were excluded: eight participants with a pure CAG tract (Q^1) modal allele > 50 CAG; 26 participants with a pure CAG < 40; four non-Caucasians; and 34 for whom clinical data was not available and ethnicity was unknown. Thus, the final cohort of Enroll-HD participants used for the description of allelic variation at the *HTT* exon one repeat locus comprised 543 individuals. Of these, 12 participants generated < 250 DNA sequencing reads for the progenitor expanded *HTT* allele and thus a somatic expansion score could not be determined. Thus, the final cohort of Enroll-HD participants used for

the genotype to phenotype analyses comprised 531 individuals, of which 141 were premanifest, and 390 manifest, for motor symptoms.

This study complies with all relevant ethical regulations. All participants were recruited with informed consent and all collaborating clinical sites are required to obtain and maintain local ethics committee approvals. The study was approved by TRACK-HD and Enroll-HD.

TRACK-HD and Enroll-HD are sponsored by CHDI Foundation, Inc., a nonprofit biomedical research organization exclusively dedicated to developing therapeutics for Huntington disease. Data used in this work was generously provided by the participants in the TRACK-HD and Enroll-HD study and made available by CHDI Foundation, Inc.

I.1.2. HD outcomes

I.1.2.1. Age at motor onset (AAO). For Track-HD, AAO was recorded as the onset of motor symptoms in HD, as determined by the Track-HD investigating neurologist based on clinical history, review of case notes and examination. For Enroll-HD, AAO was recorded as onset of motoric symptom estimated by an independent rater based on clinical history, case notes and examination. Age at motor onset was available for 105 Track-HD and 395 Enroll-HD participants.

I.1.2.2. Total motor score (TMS). TMS is a standard scale in the Unified Huntington Disease Rating Scale (UHDRS).[2] The motor section of the UHDRS assesses motor features of HD using standardized ratings of oculomotor function, dysarthria, motor impersistence, chorea, dystonia, bradykinesia, gait, and postural stability. The total motor impairment score is the sum of the individual motor ratings (range 0 to 124); higher scores indicate more severe motor impairment. Motor impairment is considered to be present once a TMS score of 10 is achieved. TMS at baseline was available for all 203 TRACK-HD participants and 531 Enroll-HD participants. Longitudinal TMS data over three years were also available for all 203 TRACK-HD participants. For linear regression analyses where baseline TMS was the dependent variable, square-root transformation of baseline TMS was used for normalisation and to minimise the influence of extreme values (W statistics of the Shapiro-Wilk test before and after transformation were 0·89 and 0·97).

I.1.2.3. Total functional capacity (TFC). The UHDRS TFC scale assesses a person with HD in terms of ability to work, complete household finances, chores and activities of daily living, and what level of care they need.[2] The scale ranges from 0 (fully dependent for all care) to 13 (fully independent). Longitudinal TFC data over three years were available for all 203 TRACK-HD participants.

I.1.2.4. HD progression score. HD progression scores were calculated for the 203 TRACK-HD participants as previously described (see particularly Figure 1B).[4] This score was determined as follows:

1) We estimated the longitudinal influence of CAG length, using either the fragment-length estimate (Q^{FL}) or the pure CAG (Q^{1}), upon each of a wide range of motor, neuropsychological, and brain imaging outcomes. The following measurements were used as outcome variables: symbol digit; Stroop word; paced tapping 3 Hz (inverse SD); spot the change 5K; emotion recognition; direct circle (log annulus length); indirect circle (log annulus length); total brain volume; ventricular volume; grey matter volume; white matter volume; caudate volume; metronome tapping, nondominant hand (log of tap initiation SD for all trials); metronome tapping, nondominant hand (inv tap initiation SD for self-paced trials); speeded tapping, nondominant hand (log of repetition time SD); speeded tapping, nondominant hand (log of tap duration SD); speeded tapping, nondominant hand (mean inter-tap time); tongue force—heavy (log coefficient of variation); tongue force—light (log coefficient of variation); grip force, dominant hand, heavy condition (log of mean orientation); grip force, dominant hand, heavy condition (log of mean position); grip force, nondominant hand, heavy condition (log of coefficient of variation); grip force, dominant hand, light condition (log of coefficient of variation); and grip force, nondominant hand, light condition (log of coefficient of variation). We used a mixed effect linear model for the visit-dependent values of each outcome. Random subject effects in these mixed linear models were accounted for using correlated random intercepts and slopes. The models used age, fragment-length estimated CAG and the age-by-CAG interaction as predictors. Further nonlinearity in relationships between CAG length and the outcomes was modelled by also using as a predictor the cumulative probability of onset statistic, which is defined as 1 minus the estimated survival probability as calculated using the fragment-length CAG-dependent age of onset formula of Langbehn *et al.*[9] Note that the use of these fragment-length CAG estimates precludes derivation of a progression score using the total glutamine length encoded that would be two repeats longer. These models also controlled for study site, sex, and education level. We accounted for the longitudinal influence of all predictor variables using their interactions with length of follow-up from the baseline visit.

2) For each separate outcome, atypical progression unaccounted for by the model in step 1 was estimated using the empirical Bayes estimated random slope for each participant.

3) We performed a principal component analysis on the estimated slopes from step 2 and found that the first component was substantially correlated ($r > 0{\cdot}40$) with the majority of the 24 outcomes listed above. Exceptions were: paced tapping 3 Hz (inverse SD); spot the change 5K; emotion recognition; direct circle (log annulus length); metronome tapping, nondominant hand (inv tap initiation SD for self-paced trials); speeded tapping, nondominant hand (log of repetition time SD); speeded tapping, nondominant hand (log of repetition time SD); speeded tapping, nondominant hand (log of tap duration SD); speeded tapping, nondominant hand (mean inter-tap time); tongue force—heavy (log coefficient of variation); and tongue force—light (log coefficient of variation).

4) The first principal component score from step 3 was defined as the atypical progression score.

In the main text, we refer to this measure of atypical progression as the 'progression score'. One unit increase in progression score in TRACK-HD corresponded to an increase of 0·71 units year^{-1} (95% CI 0·34 to 1·08) in the rate of change of TMS, and an increase of approximately 0·2 units year^{-1} (95% CI 0·12 to 0·30) in the rate of change of TFC.[4]

I.1.3. *HTT* exon one repeat region sequencing and genotyping

The *HTT* exon one repeat region was amplified from 20 ng of blood DNA (whole blood from TRACK-HD and buffy coat from Enroll-HD) using MiSeq-compatible PCR primers.[10] TruSeq CD indexes allowing the sequencing of up to 96 samples per MiSeq run were used for the TRACK-HD samples (see table 1 in Ciosi *et al.*).[10] Nextera XT Index Kit v2 indexes allowing the sequencing of up to 384 samples per MiSeq run were used for the Enroll-HD samples (see table 2 in Ciosi *et al.*).[10] See Ciosi *et al.*,[10] for the full details of the sequencing library preparation and MiSeq sequencing. MiSeq library preparation for the TRACK-HD cohort was undertaken at the University of Glasgow and sequencing was performed by Glasgow Polyomics (http://www.polyomics.gla.ac.uk). MiSeq library preparation and sequencing for the Enroll-HD cohort was performed at Q^2 Solutions – EA Genomics (https://www.q2labsolutions.com/genomics-laboratories) using the same methods. A separate batch of replicate samples was sequenced at both centres with 100% concordance for the sequence and modal length of alleles $Q^1 \leq 52$.

I.1.4. Genotyping of the *HTT* exon one repeat region

The *HTT* exon one repeat region was genotyped from the MiSeq reads generated using ScaleHD (version 0.251)(AM, MC and DGM., manuscript in preparation). ScaleHD is a bioinformatics pipeline for the automated genotyping of high-throughput next-generation sequencing reads of the *HTT* exon one repeat region. ScaleHD is a collection of open-source software, combined with internally-developed modules to genotype individuals from *HTT* amplicon sequencing data. Prior to processing them through ScaleHD, reads were demultiplexed using cutadapt (version 1.9.1) using the options -g, -e 0, --overlap 10 and --discard-untrimmed.[11] Forward (*i.e.* Read 1) and reverse (*i.e.* Read 2) reads were respectively demultiplexed based on the first 5'-bases of the HD319F and 33935.5 locus-specific PCR primers.[10] This initial 5'-demultiplexing/trimming step removes reads that do not start with a PCR primer binding site and trims all the reads at the same position within the PCR primer binding site. This allows processing of reads, through ScaleHD, which start at the same position and allows for trimming of the spacer which is present 5' of the locus-specific primer in the sequencing reads.[10] Reads were then processed through ScaleHD. The ScaleHD configuration and parameter values used can be found below (supplementary text I.1.5).

The ScaleHD pipeline was used to remove the Illumina sequencing adapter at the 3'-end of the reads. Resultant trimmed forward reads for each sample were then aligned by ScaleHD using BWA-MEM against a library of 4,000 *HTT* reference sequences, each with a typical allele structure Q^1-1-1-P^2-2 (table 1), but each with a unique Q^1/P^2 combination with $1 \leq Q^1 \leq 200$ and $1 \leq P^2 \leq 20$. Each of these 4,000 reference sequences also extends to the binding sites of the HD319F and 33935.5 locus-specific PCR primers, respectively 5' and 3' of the *HTT* exon one repeat region. In the same way, reverse reads for a sample were then aligned to a library of 20 references in which $Q^1 = 100$ and $1 \leq P^2 \leq 20$. The reverse read alignments are less complex than the forward, which allowed the genotyping of the *HTT* CCG repeat independently from the CAG repeat. ScaleHD then scanned the forward and reverse alignment maps with digital signal processing to determine the literal structure of the *HTT* exon one repeat region. If known atypical allele structures were detected (table 1),[12] re-alignment to custom dynamically-generated atypical reference libraries was performed for confirmation. ScaleHD informs the user if an unknown atypical allele structure is detected, *i.e.* a structure different from Q^1-Q^2-P^1-P^2-P^3 or with $Q^2 > 2$, $P^2 > 1$ or $P^3 > 3$. ScaleHD then utilises data from digital signal processing to guide machine-learning driven genotyping modules towards the correct classification for the data (AM, MC and DGM, manuscript in preparation). The forward read count distribution, *i.e.* the distribution of the number of forward

reads aligned to each of the 4,000 references considered, for all the TRACK-HD samples was manually plotted and inspected to deduce the genotype of the *HTT* exon one repeat region to confirm ScaleHD genotyping. For all the TRACK-HD and Enroll-HD samples, the alignment of forward reads to the two reference sequences corresponding to one non-disease associated allele and one disease-associated allele were visually inspected in Tablet (version 1.17.08.17).[13] Visual inspection was performed for all alignments to a typical reference library and all the re-alignments to an atypical reference library.

I.1.5. ScaleHD *HTT* genotyping configuration file


```
<config data_dir="/TRACK-HDandEnrollHD_MiSeqdata_DMPXedWithCutadapt191" forward_reference="4k-HD-INTER.fas" reverse_reference="RV_CAG-1-1-CCG-2.fasta">
  <instance_flags quality_control="True" sequence_alignment="True" atypical_realignment="True" genotype_prediction="True" snp_calling="False"/>
	<trim_flags trim_type="Adapter" quality_threshold="5" adapter_flag="-a" forward_adapter="GATCGGAAGAGCACACGTCTGAACTCCAGTCAC" reverse_adapter="AGATCGGAAGAGCGTCGTGTAGGGAAAGAGTGT" error_tolerance="0.39"/>
  <alignment_flags min_seed_length="19" band_width="100" seed_length_extension="1.5" skip_seed_with_occurrence="500" chain_drop="0.50" seeded_chain_drop="0" seq_match_score="1" mismatch_penalty="4" indel_penalty="6,6" gap_extend_penalty="4,4" prime_clipping_penalty="5,5" unpaired_pairing_penalty="17"/>
  <prediction_flags plot_graphs="True"/>
</config>
```


I.1.6. Single-molecule sequencing experiment

HD patients carrying alleles of the typical structure Q^1-1-1-7-2 and Q^1 between 40 and 50 were selected based on genotypes of the *HTT* exon one repeat obtained as described above. For blood DNA samples from these selected patients, DNA concentrations were determined using the Qubit HS kit (Thermo Fisher UK Ltd). An equimolar pool of genomic DNA was then prepared, and the pooled DNA was digested with 10 units HindIII (NEB UK Ltd, 37°C for 2 h followed by heat inactivation at 65°C for 20 min), then diluted to 5 pg μl^{-1} for PCR. Each PCR plate included a 1 ng μl^{-1} positive control, five no-template controls (NTC) and 90 separate reactions each containing 5 pg pooled genomic DNA template. The *HTT* exon one repeat region was amplified by PCR for 10 cycles using the HD319F and 33935.5 primers,[10] then each reaction was split into two aliquots of equal volume. More Taq polymerase, primers, DMSO and 'Custom PCR Master Mix+βME' were added to aliquot 1 to the appropriate concentrations,[10] and 19 further cycles of amplification were performed on aliquot 1 using the same primers. The aliquot 1 PCR products were resolved by electrophoresis, Southern blotted and hybridised with a CTG•CAG repeat probe essentially as described.[14] From the autoradiographs, aliquot 1 reactions that had a single band, or two widely spaced bands, amplified from disease-associated alleles were selected for further analysis. Aliquot 2 from each of the selected single molecule-containing reactions, as well as the NTCs, were digested with 10 U ExoI to destroy the primers (NEB UK Ltd, 37°C for 1 h, followed by heat inactivation at 80°C for 10 min). MiSeq-compatible PCR primers with TruSeq CD indexes and all other PCR reagents, as described previously, were then added to each aliquot 2 sample to restore the appropriate concentrations in the final reactions, and 19 further cycles of PCR were carried out as described by Ciosi *et al.*[10] Sequencing library preparation steps post-PCR were performed as described by Ciosi *et al.*,[10] except for the fact that the aliquot 2 sequencing was mixed with another sequencing library of higher DNA concentration because its concentration was too low to be sequenced on its own. *HTT* exon one repeat region reads were then genotyped using ScaleHD as described in the previous section.

I.1.7. Quantification of the ratio of somatic expansions of disease-associated alleles

From the MiSeq read count distribution obtained for each disease-associated allele sequenced (*e.g.* figure S3B) we quantified the ratio of somatic expansions using the measure $\frac{\sum_{i=1}^{10} n_{+i}}{n}$, where n is the number of MiSeq reads corresponding to the progenitor allele (for example $(CAG)_{44}$ in figure S3) and n_{+i} is the number of MiSeq reads corresponding to the sequenced variants with i more CAG repeats than the progenitor allele. This measure of somatic expansions is thus the relative ratio of somatic variants that have gained one to ten CAG repeats over the number of repeats in the progenitor allele. Somatic variants with more CAG repeats than the progenitor allele were quantified in the interval $[i = 1, i = 10]$ because this interval included 99·9% of the sequenced reads longer than the progenitor allele in our MiSeq data obtained from blood DNA. The relative ratio of somatic expansions of disease-associated alleles was quantified in TRACK-HD and Enroll-HD participants for which $n \geq 250$ reads. n was < 250 reads for only 12 Enroll-HD participants. For linear regression analyses, ln-transformation of the somatic expansion ratio was used for normalisation and to minimise the influence of extreme values (W statistics of the Shapiro-Wilk test before and after transformation were 0·78 and 0·97).

I.1.8. Determination of the somatic expansion score of disease-associated alleles

Individual-specific somatic expansion scores were defined as the residual variation in the ratio of somatic expansions corrected for sex, cohort, and an interaction between age at sampling and length of the pure CAG repeat (Q^1) (model SEQ1 in table S2, appendix).

I.1.9. Selection of candidate SNPs and SNP genotyping in the Enroll-HD cohort

Single-nucleotide polymorphisms (SNP) were selected as previously defined by Bettencourt *et al.*,[15] as "the most significant genes (gene-wide, $p < 0{\cdot}1$) in the 'DNA repair pathway cluster' from the GeM-HD analysis" as well as SNPs in *RRM2B* and *UBR5*. We also selected SNPs that were associated with a genome-wide $p < 10^{-6}$ in the GeM-HD analysis[16], as well as an *MSH3* variant associated with HD progression[4]. In the discovery TRACK-HD cohort, out of 31 SNPs meeting the above criteria, genotypes for 28 SNPs were available[4] (tables 3 and S7, appendix). SNPs in the Enroll-HD replication cohort were genotyped using a KASP assay (LGC Genomics). The top *MSH3* SNP of the genome-wide association study of HD progression (rs557874766)[4] is located in a 9 bp tandem repeat which is not suitable for SNP genotyping by KASP. We, therefore, selected *MSH3* rs1382539 instead of rs557874766 because both SNPs are in high linkage disequilibrium ($r^2 = 0{\cdot}97$, D' = 1 in the TRACK-HD cohort) and because rs1382539 genotypes were available for the TRACK-HD cohort. SNPs considered for replication in the Enroll-HD cohort were selected as the top eight most significantly associated SNPs ($p < 0{\cdot}1$) in the TRACK-HD cohort in a preliminary analysis using a slightly larger TRACK-HD cohort including four participants with 39 pure CAG repeats ($Q^1 = 39$) and six non-Caucasians. In the preliminary analysis, the association between somatic expansion score and rs20579 in *LIG1* was $p = 0.072$, and rs20579 was thus selected for replication in Enroll-HD. Conversely, in the same preliminary analysis, rs11061229 and rs72810940 did not reach the selection threshold of $p < 0{\cdot}10$, and thus were not chosen for replication in Enroll-HD. KASP assays were already available for four out of these eight SNPs[4] (*MLH3* rs175080, *FAN1* rs3512, *LIG1* rs20579 and *MLH1* rs1799977). KASP assay design and validation was attempted by LGC Genomics for the remaining four SNPs (*MTMR10* rs2140734, *RP11-481J13.1* rs147804330, *MSH3* rs1382539 and *MLH1* rs144287831). KASP assays could not be validated for *MTMR1*0 rs2140734 and *MLH1* rs144287831. Six candidate SNPs were thus genotyped in the Enroll-HD replication cohort. SNP genotyping using these six KASP assays was performed by LGC Genomics. Hardy-Weinberg equilibrium tests were performed as quality control of the KASP genotyping for these six SNPs. None of these six SNPs was in significant deviation from Hardy-Weinberg equilibrium after correction for multiple testing (*p*-values > $0{\cdot}22$ after correction for multiple testing).

I.1.10. Availability of code and software used

The *HTT* exon one repeat region was genotyped from the MiSeq reads generated using ScaleHD (version 0.251) (https://github.com/helloabunai/ScaleHD). ScaleHD utilises a number of Python dependencies: cutadapt (version 1.9.1) (http://cutadapt.readthedocs.io/en/v1.9.1/); generatr (version 0.252) (https://github.com/helloabunai/RefGeneratr); lxml (version 4.0.0) (http://lxml.de); numpy (version 1.13.1) (http://www.numpy.org); pandas (version 0.14.1) (https://pandas.pydata.org); peakutils (version 1.0.3) (https://pypi.org/project/PeakUtils/1.0.3); pysam (version 0.9.1.4) (https://github.com/pysam-developers/pysam); regex (version 2017.1.17) (https://pypi.org/project/regex/); scipy (version 0.17.1) (https://www.scipy.org/scipylib/index.html); and sklearn (version 0.19.1) (http://scikit-learn.org/stable/). In addition to a number of third party binaries: Java (version 1.8.0_20)(https://java.com/en/); SeqTK (version 1.2-r101-dirty) (https://github.com/lh3/seqtk); BWA-MEM (version 0.7.15-r1140)(https://github.com/lh3/bwa/releases/tag/v0.7.15); and Samtools (version 1.3.1) (https://sourceforge.net/projects/samtools/files/samtools/1.3.1/). Sequence alignments were visually inspected in Tablet (version 1.17.08.17) (https://ics.hutton.ac.uk/tablet/). Statistical analyses were undertaken in R (version 3.4.3) (https://www.r-project.org) using RStudio (version 1.0.153) (https://www.rstudio.com). Genetic association studies were undertaken using PLINK (version 1.07) through gPLINK (version 2.050) (http://zzz.bwh.harvard.edu/plink/gplink.shtml). The meta-analysis of the SNP association tests was performed using METAL (http://csg.sph.umich.edu/abecasis/metal/).

R functions and packages: multiple linear regressions were performed using the function stats::lm; comparison of linear models and Cox regressions were performed using the stats::anova function; least-square means and their confidence intervals were estimated using the emmeans package (version 1.3.4);[17] Cox proportional hazard regressions were performed using the survival::coxph function (version 2.38);[18,19] the proportional hazard assumption for each Cox regression model fit and each covariate was tested using the survival::cox.zph function;[18,19] adjusted survival curves were produced using the survminer package (version 0.4.3);[20] bootstrapping and estimation of the confidence interval of the difference between goodness of fit statistics were performed using the boot package (version 1.3-22);[21,22] and mixed effect models were performed using the function lme4::lmer.[23]

II. Supplementary tables

Table S1: Literature review of atypical *HTT* exon one repeat structures and their association with Huntington disease clinical outcomes and genetic instability. This literature review was performed as described in the Research in Context section.

Reference	Atypical allele structure(s) described	Number of atypical HD-causing alleles	Association with clinical outcomes, germline instability and/or somatic instability
Pêcheux *et al*., 1995[24]	$(CAG)_n$ --- $(CAACAG)_2(CCGCCA)_1(CCG)_7$	2	Not investigated.
Goldberg *et al*., 1995[25]	$(CAG)_n$--- $(CAACAG)_0(CCGCCA)_0(CCG)_9$	4 from one family	Germline instability: unusually large intergenerational jump from an intermediate allele into the disease range within one family.
Gellera *et al.,* 1996[26]	$(CAG)_{45}$--- $(CAACAG)_0(CCGCCA)_1(CCG)_{10}$<br>$(CAG)_n$ --- $(CAACAG)_0(CCGCCA)_1(CCG)_n$	3	Not investigated.
Chong *et al*., 1997[27]	$(CAG)_{33}$--- $(CAACAG)_0(CCGCCA)_0(CCG)_{12}$<br>$(CAG)_{36}$--- $(CAACAG)_0(CCGCCA)_0(CCG)_9$	2 from two families	Germline instability: higher frequency of sperm carrying HD-causing alleles in two individuals, carrying intermediate atypical alleles (the individual carrying an allele with nine CCGs belonged to the family described by Goldberg *et al.* 1995).
Margolis *et al*., 1999[28]	$(CAG)_{27}$--- $(CAACAG)_1(CCGCCA)_0(CCG)_{12}$	1	Not investigated.
Kelly *et al*., 1999[29]	$(CAG)_n$--- $(CAACAG)_0(CCGCCA)_0(CCG)_9$	1 from one family	Germline instability: unusual germline expansion from an intermediate 27-repeat allele to a disease-associated 38-repeat allele.
Williams *et al.,* 2000[30]	$(CAG)_n$ --- $(CAACAG)_0(CCGCCA)_0(CCG)_n$	8 from three families	Clinical outcome: One individual with 37 pure CAGs with mild symptoms of HD.<br>Germline instability: Allele structure not associated with particularly high germline instability.<br>Somatic instability: Allele structure not associated with somatic mosaicism.
Yu *et al.,* 2000[12]	$(CAG)_n$ --- $(CAACAG)_2(CCGCCA)_1(CCG)_n$<br>$(CAG)_{19}$CAA $(CAACAG)_1(CCGCCA)_1(CCG)_n$	5	Not investigated.
Nørremølle *et al*. 2009[31]	$(CAG)_n$ --- $(CAACAG)_2(CCGCCA)_1(CCG)_7$	1	Clinical outcome: The *HTT* haplotype of the atypical allele sequenced is the same as the HD-onset-delaying haplotype described in 47 other individuals.
Houge *et al*., 2013[32]	$(CAG)_n$ --- $(CAACAG)_1(CCGCCA)_0(CCG)_9$	2 from one family	Germline instability: Unusual germline expansion from a 26-repeat allele to a disease-associated 44 repeat allele.
Bečanović *et al*., 2015[33]	$(CAG)_n$ --- $(CAACAG)_2(CCGCCA)_1(CCG)_7$	26	Clinical outcome: The atypical alleles sequenced are in linkage disequilibrium with the rs13102260 minor (A) variant which is associated with *HTT* expression and HD onset.

Table S2: Linear regression models of the relationships between allele length, age and sequence structures with somatic expansions in Huntington disease. The table shows the squared coefficient of correlation (r^2, raw and adjusted) and statistical significance (p) for each model, and the coefficient and 95% confidence interval, t-statistic (t), statistical significance (p) and the p-value adjusted for multiple testing using the Benjamini-Hochberg false discovery rate correction[34] ($pFDR$) associated with each parameter in the model. The coefficient provides an indication of the relative weight of the contribution, and effect size, of each parameter to the model and its associated 95% confidence interval. The t-statistic and corresponding p-value provide an indication of the statistical significance that the parameter is adding explanatory power to the model. SE: ratio of somatic expansions. Q^T: total number of glutamines in the disease-associated allele (centred values). Q^1: number of pure CAGs in the disease-associated allele (centred values). Age: age at DNA sampling in years (centred values). Sex: factor, male or female. Cohort: factor, TRACK-HD (n = 203) or Enroll-HD (n = 531). Q^2: factor, number of additional glutamine codons in the disease-associated allele, 0 (n = 7), 2 (n = 714) or 4 (n = 13). Models SEQ^1 and SEQ^1Q^2 were compared with an ANOVA and the p-value associated with the F-statistic was then estimated based on 10^5 permutations of Q^2 (F = 1·93, p = 0·10). $^+$: p-values were estimated using 10^5 permutations of the number of additional glutamine codons (Q^2).

	Model	r^2	Adjusted r^2	Model p	Parameter values						
					**Parameter**	**Coefficient**	**Lower 95% CI**	**Upper 95% CI**	***t***	***p***	***pFDR***
SEQ^T	*ln(SE) ~ Q^T + Age + Q^T x Age + Sex + Cohort*	0·822	0·821	$<2 \times 10^{-16}$	intercept	-0·987	-1·010	-0·964	-85·1	$<2 \times 10^{-16}$	$<2 \times 10^{-16}$
					Q^T	0·209	0·201	0·217	51·7	$<2 \times 10^{-16}$	$<2 \times 10^{-16}$
					Age	0·017	0·015	0·018	21·9	$<2 \times 10^{-16}$	$<2 \times 10^{-16}$
					Q^T x Age	0·000	$-3{\cdot}9 \times 10^{-5}$	0·001	1·8	0·069	0·12
					Sex = male	0·009	-0·020	0·038	0·58	0·56	0·62
					Cohort = TRACK-HD	-0·014	-0·047	0·019	-0·85	0·40	0·51
SEQ^1	*ln(SE) ~ Q^1 + Age + Q^1 x Age + Sex + Cohort*	0.836	0·835	$<2 \times 10^{-16}$	intercept	-0·986	-1·008	-0·964	-88·2	$<2 \times 10^{-16}$	$<2 \times 10^{-16}$
					Q^1	0·220	0·212	0·228	54·0	$<2 \times 10^{-16}$	$<2 \times 10^{-16}$
					Age	0·018	0·016	0·019	24·1	$<2 \times 10^{-16}$	$<2 \times 10^{-16}$
					Q^1 x Age	0·001	0·000	0·001	3·3	0·001	0·002
					Sex = male	0·010	-0·018	0·038	0·72	0·47	0·56
					Cohort = TRACK-HD	-0·002	-0·033	0·030	-0·11	0·91	0·91

Continued

Table S2: continued

	Model	r^2	Adjusted r^2	Model *p*	Parameter values						
					**Parameter**	**Coefficient**	**Lower 95% CI**	***Upper 95% CI***	***t***	***p***	***pFDR***
SEQ¹Q²	*ln(SE) ~ Q¹ + Age + Q¹ x Age + Sex + Cohort + Q² + Q² x Age*	0·838	0·835	$<2 \times 10^{-16}$	intercept	-0·987	-1·009	-0·965	-88·2	$<2 \times 10^{-16}$	$<2 \times 10^{-16}$
					Q^1	0·219	0·211	0·227	53·5	$<2 \times 10^{-16}$	$<2 \times 10^{-16}$
					Age	0·018	0·016	0·019	23·8	$<2 \times 10^{-16}$	$<2 \times 10^{-16}$
					Q^1 x Age	0·001	0·000	0·001	3·1	0·002	0·004
					Sex = male	0·010	-0·018	0·038	0·70	0·48	0·56
					Cohort = TRACK-HD	-0·002	-0·034	0·029	-0·14	0·89	0·91
					Q^2 = 0	-0·085	-0·227	0·057	-1·17	$0·23^+$	0·36
					Q^2 = 4	0·063	-0·044	0·171	1·16	$0·24^+$	0·36
					Q^2 = 0 x Age	-0·011	-0·023	0·000	-1·89	$0·06^+$	0·12
					Q^2 = 4 x Age	0·005	-0·006	0·015	0·86	$0·40^+$	0·51

Table S3: Cox regression models of the relationship between allele length, sequence structure and somatic expansion scores with time to onset of Huntington disease motor symptoms. The table shows the number of events (motor onset), log-likelihood, statistical significance (*p*) for each model, and the hazard ratio and 95% confidence interval, *z*-statistic (*z*), statistical significance (*p*) and the *p*-value adjusted for multiple testing using the Benjamini-Hochberg false discovery rate correction[34] (*pFDR*), associated with each parameter in the model. The hazard ratio indicates the relative increase in the risk of motor onset associated with a 1 unit increase in the parameter. The *z*-statistic and corresponding *p*-value provide an indication of the statistical significance that the parameter is adding explanatory power to the model. Q^T: total number of glutamines in the disease-associated allele. Q^1: number of pure CAGs in the disease-associated allele. Q^{FL}: number of CAGs estimated by fragment length analysis. SEQ^1: somatic expansion score (residuals of model SEQ1 (table S2, appendix), *i.e.* residual variation in the somatic expansion ratio not accounted for by Q^1, age at sampling and their interaction). Q^2: factor, number of additional glutamine codons in the disease-associated allele (Q^2) 0 (n = 7), 2 (n = 714) or 4 (n = 13). Sex (male or female) and cohort (TRACK-HD (n = 203) or Enroll-HD (n = 531)) were used as strata in the analyses. Models $AAOQ^1$ and $AAOQ^1Q^2$ were compared with an ANOVA and the *p*-value associated with the χ^2-statistic was then estimated based on 10^5 permutations of Q^2 ($\chi^2 = 11{\cdot}3$, $p = 0{\cdot}002$). $^+$: *p*-values were estimated using 10^5 permutations of the number of additional glutamine codons (Q^2).

Model		Events	Log-likelihood	Model *p*	Parameter values						
					Parameter	Hazard ratio	Lower 95% CI	Upper 95% CI	*z*	*p*	*pFDR*
$AAOQ^T$	*time to HD motor signs ~* Q^T *+* SEQ^1 *+ strata(Sex) + strata(Cohort)*	488	-1,899	<2 x 10^{-16}	Q^T	1·71	1·63	1·80	21·96	<2 x 10^{-16}	<2 x 10^{-16}
					SEQ^1	2·56	1·61	4·05	3·99	6·6 x 10^{-5}	8·8 x 10^{-5}
$AAOQ^1$	*time to HD motor signs ~* Q^1 *+* SEQ^1 *+ strata(Sex) + strata(Cohort)*	488	-1,884	<2 x 10^{-16}	Q^1	1·80	1·69	1·87	22·48	<2 x 10^{-16}	<2 x 10^{-16}
					SEQ^1	2·81	1·79	4·42	4·48	7·6 x 10^{-6}	1·3 x 10^{-5}
$AAOQ^1Q^2$	*time to HD motor signs ~* Q^1 *+* SEQ^1 *+ strata(Sex) + strata(Cohort) +* Q^2	488	-1,878	<2 x 10^{-16}	Q^1	1·79	1·70	1·89	22·64	<2 x 10^{-16}	<2 x 10^{-16}
					SEQ^1	3·05	1·94	4·80	4·84	1·3 x 10^{-6}	2·8 x 10^{-6}
					$Q^2 = 0$	5·19	2·38	11·32	4·14	6·1 x 10^{-4+}	7·3 x 10^{-4}
					$Q^2 = 4$	1·02	0·56	1·88	0·07	0·95$^+$	0·95
$AAOQ^{FL}Q^2$	*time to HD motor signs ~* Q^{FL} *+* SEQ^1 *+ strata(Sex) + strata(Cohort) +* Q^2	488	-1,878	<2 x 10^{-16}	Q^{FL}	1·79	1·70	1·89	22·64	<2 x 10^{-16}	<2 x 10^{-16}
					SEQ^1	3·05	1·94	4·79	4·82	1·4 x 10^{-6}	2·8 x 10^{-6}
					$Q^2 = 0$	16·65	7·48	37·06	6·89	<1 x 10^{-5+}	<1·5 x 10^{-5}
					$Q^2 = 4$	0·57	0·31	1·05	-1·79	0·10$^+$	0·11

Table S4: Linear regression models of the relationship between sequence structure and somatic expansions scores with disease progression in Huntington disease. The table shows the squared coefficient of correlation (r^2, raw and adjusted) and statistical significance (p) for each model, and the coefficient and 95% confidence interval, t-statistic (t), statistical significance (p) and p-value adjusted for multiple testing using the Benjamini-Hochberg false discovery rate correction[34] (*pFDR*) associated with each parameter in the model. The coefficient provides an indication of the relative weight of the contribution and effect size of each parameter to the model and its associated 95% confidence interval. The t-statistic and corresponding p-value provide an indication of the statistical significance that the parameter is adding explanatory power to the model. SEQ^1: somatic expansion score (residuals of model SEQ1 (table S2, appendix), *i.e.* residual variation in SE not accounted for by Q^1, age at sampling and their interaction). Q^{FL}Prog: TRACK-HD progression score based on the fragment length estimated number of CAGs (Q^{FL}) in the disease-associated allele. Q^1Prog: TRACK-HD progression score based on the number of pure CAGs (Q^1) in the disease-associated allele. Sex: factor, male or female. Cohort: TRACK-HD (n = 203). Q^2: factor, number of additional glutamine codons in the disease-associated allele (Q^2) 0 (n = 2), 2 (n = 195) or 4 (n = 6). $^{+}$: p-values were estimated using 10^5 permutations of the number of additional glutamine codons (Q^2).

	Model	*r2*	Adjusted *r2*	Model *p*	Parameter values						
					**Parameter**	**Coefficient**	**Lower 95% CI**	**Upper 95% CI**	***t***	***p***	***pFDR***
ProgQ^1	$Q^1Prog \sim Q^2 + SEQ^1 + Sex$	0·098	0·080	3·9 x 10^{-4}	intercept	-0·001	-0·181	0·179	-0·01	0·991	0·991
					Q^2 = 0	2·237	0·891	3·582	3·28	0·003^{+}	0·014
					Q^2 = 4	-0·743	-1·535	0·049	-1·85	0·065^{+}	0·110
					SEQ^1	0·983	0·243	1·722	2·62	0·009	0·028
					Sex = male	0·002	-0·267	0·272	0·02	0·987	0·991
ProgQ^{FL}	$Q^{FL}Prog \sim Q^2 + SEQ^1 + Sex$	0·153	0·136	1·1 x 10^{-6}	intercept	-0·008	-0·183	0·167	-0·09	0·927	0·991
					Q^2 = 0	3·127	1·824	4·431	4·73	<1 x 10^{-5+}	<1 x 10^{-4}
					Q^2 = 4	-0·990	-1·758	-0·223	-2·55	0·013^{+}	0·028
					SEQ^1	0·903	0·186	1·620	2·48	0·014	0·028
					Sex = male	0·015	-0·247	0·276	0·11	0·912	0·991

Table S5: Linear regression models of the relationship between sequence structure and somatic expansions scores with the rate of change of TMS and TFC in Huntington disease. The table shows the squared coefficient of correlation (r^2, raw and adjusted) and statistical significance (*p*) for each model, and the coefficient and 95% confidence interval, *t*-statistic (*t*), statistical significance (*p*) and the *p*-value adjusted for multiple testing using the Benjamini-Hochberg false discovery rate correction[34] (*pFDR*) associated with each parameter in the model. The coefficient provides an indication of the relative weight of the contribution of each parameter to the model and its associated 95% confidence interval. The *t*-statistic and corresponding *p*-value provide an indication of the statistical significance that the parameter is adding explanatory power to the model. TMSrate and TFCrate were derived from three-year longitudinal data using random slope and random intercept mixed effect models (with a fixed 'years of follow-up' effect and a random 'participant' effect) to estimate the rate of change of TMC and TFC for each participant. Q^1: number of pure CAGs in the disease-associated allele (centred values). BaselineTMS: TMS at baseline (centred values). BaselineTFC: TFC at baseline (centred values). Age_b: Age at baseline in years (centred values). Sex: factor, male or female. SEQ^1: somatic expansion score (residuals of model SEQ1 (table S2, appendix), *i.e.* residual variation in SE not accounted for by Q^1, age at sampling and their interaction). Q^2: number of additional glutamine codons in the disease-associated allele (Q^2) 0 ($n = 2$), 2 ($n = 195$), or 4 ($n = 6$). Cohort: TRACK-HD ($n = 203$). $^+$: *p*-values were estimated using 10^5 permutations of the number of additional glutamine codons (Q^2).

	**Model**	r^2	**Adjusted r^2**	**Model *p***	**Parameter values**						
					**Parameter**	**Coefficient**	**Lower 95% CI**	**Upper 95% CI**	***t***	***p***	***pFDR***
TMSrateQ^1Q^2	*TMSrate ~ BaselineTMS + Age_b + Q^1 + Age_b * Q^1 + Q^2 + SEQ^1 + Sex*	0·434	0·410	<2 x 10^{-16}	intercept	2·102	1·829	2·374	15·211	<2 x 10^{-16}	<2 x 10^{-16}
					BaselineTMS	0·042	0·020	0·063	3·865	1·5 x 10^{-4}	4·5 x 10^{-4}
					Age_b	0·068	0·030	0·106	3·501	5·8 x 10^{-4}	1·5 x 10^{-3}
					Q^1	0·461	0·294	0·629	5·433	1·7 x 10^{-7}	9·6 x 10^{-7}
					Age_b * Q^1	0·008	0·000	0·017	1·874	0·062	0·093
					$Q^2 = 0$	0·946	-0·916	2·808	1·002	0·255$^+$	0·328
					$Q^2 = 4$	-0·254	-1·359	0·851	-0·454	0·627$^+$	0·664
					SEQ^1	1·743	0·730	2·755	3·394	8·4 x 10^{-4}	1·9 x 10^{-3}
					Sex = male	-0·296	-0·667	0·076	-1·571	0·118	0·163
TFCrateQ^1Q^2	*TFCrate ~ BaselineTFC + Age_b + Q^1 + Age_b * Q^1 + Q^2 + SEQ^1 + Sex*	0·307	0·278	1·8 x 10^{-12}	intercept	-0·354	-0·446	-0·262	-7·573	1·4 x 10^{-12}	1·3 x 10^{-11}
					BaselineTFC	0·051	0·007	0·094	2·277	0·024	0·039
					Age_b	-0·030	-0·041	-0·019	-5·380	2·1 x 10^{-7}	9·6 x 10^{-7}
					Q^1	-0·129	-0·178	-0·080	-5·188	5·3 x 10^{-7}	1·9 x 10^{-6}
					Age_b * Q^1	-0·004	-0·007	-0·001	-2·730	0·007	0·014
					$Q^2 = 0$	-0·830	-1·453	-0·208	-2·632	0·021$^+$	0·038
					$Q^2 = 4$	0·146	-0·227	0·519	0·771	0·439$^+$	0·527
					SEQ^1	-0·118	-0·463	0·226	-0·677	0·499	0·561
					Sex = male	-0·003	-0·128	0·123	-0·044	0·965	0·965

Table S6: Linear regression models of the relationship between sequence structure and somatic expansions scores with TMS in Huntington disease. The table shows the squared coefficient of correlation (r^2, raw and adjusted) and statistical significance (p) for each model, and the coefficient and 95% confidence interval, t-statistic (t), statistical significance (p) and p-value adjusted for multiple testing using the Benjamini-Hochberg false discovery rate correction[34] ($pFDR$) associated with each parameter in the model. The coefficient provides an indication of the relative weight of the contribution of each parameter to the model and its associated 95% confidence interval. The t-statistic and corresponding p-value provide an indication of the statistical significance that the parameter is adding explanatory power to the model. Sqrt(TMS_b): The square-root of TMS at baseline. Cohort: factor, TRACK-HD (n = 203) or Enroll-HD (n = 531). SEQ^1: somatic expansion score (residuals of model SEQ1 (table S2, appendix), *i.e.* residual variation in SE not accounted for by Q^1, age at sampling and their interaction). Q^T: total number of encoded-glutamines in the disease-associated allele (centred values). Q^1: number of pure CAGs in the disease-associated allele (centred values). Q^{FL}: number of CAGs estimated by fragment length analysis (centred values). Age_b: Age at baseline in years (centred values). Sex: factor, male or female. Q^2: factor, number of additional glutamine codons in the disease-associated allele (Q^2) 0, (n = 7), 2 (n = 714) or 4 (n = 13). Models $TMSQ^1$ and $TMSQ^1Q^2$ were compared with an ANOVA and the p-value associated with the F-statistic was estimated based on 10^5 permutations of Q^2 (F = 7·66, p = 6·3 x 10^{-4}).

	Model	r^2	Adjusted r^2	Model p	Parameter values						
					Parameter	Coefficient	Lower 95% CI	Upper 95% CI	t	p	$pFDR$
$TMSQ^T$	*Sqrt(TMS_b) ~ Age_b + Q^T + Age_b * Q^T + SEQ^1 + Sex + Cohort*	0·558	0·554	<2 x 10^{-16}	intercept	4·184	4·003	4·365	45·4	<2 x 10^{-16}	<2 x 10^{-16}
					Age_b	0·167	0·155	0·178	27·6	<2 x 10^{-16}	<2 x 10^{-16}
					Q^T	0·650	0·587	0·713	20·2	<2 x 10^{-16}	<2 x 10^{-16}
					Age_b * Q^T	0·013	0·009	0·017	5·9	6 x 10^{-9}	1·2 x 10^{-8}
					SEQ^1	0·971	0·369	1·574	3·2	1.6 x 10^{-3}	2·0 x 10^{-3}
					Sex = male	-0·019	-0·250	0·212	-0·2	0·871	0·911
					Cohort = TRACK-HD	-0·560	-0·820	-0·300	-4·2	3 x 10^{-5}	4·6 x 10^{-5}
$TMSQ^1$	*Sqrt(TMS_b) ~ Age_b + Q^1 + Age_b * Q^1 + SEQ^1 + Sex + Cohort*	0·578	0·575	<2 x 10^{-16}	intercept	4·205	4·027	4·382	46·5	<2 x 10^{-16}	<2 x 10^{-16}
					Age_b	0·173	0·161	0·184	28·8	<2 x 10^{-16}	<2 x 10^{-16}
					Q^1	0·706	0·642	0·771	21·5	<2 x 10^{-16}	<2 x 10^{-16}
					Age_b * Q^1	0·015	0·011	0·019	7·0	7 x 10^{-12}	1·5 x 10^{-11}
					SEQ^1	1·066	0·478	1·655	3·6	4 x 10^{-4}	5·1 x 10^{-04}
					Sex = male	-0·017	-0·243	0·209	-0·1	0·883	0·911
					Cohort = TRACK-HD	-0·515	-0·769	-0·260	-4·0	8 x 10^{-5}	1·3 x 10^{-4}

Continued

Table S6: continued

	Model	r^2	Adjusted r^2	Model p	Parameter values						
					**Parameter**	**Coefficient**	**Lower 95% CI**	**Upper 95% CI**	***t***	***p***	***pFDR***
TMSQ[1]Q[2]	*Sqrt(TMS$_b$) ~ Age$_b$ + Q^1 + Age$_b$ * Q^1 + Q^2 + SEQ^1 + Sex + Cohort*	0·558	0·554	$<2 \times 10^{-16}$	intercept	4·195	4·019	4·371	46·8	$<2 \times 10^{-16}$	$<2 \times 10^{-16}$
					Age$_b$	0·173	0·162	0·185	29·1	$<2 \times 10^{-16}$	$<2 \times 10^{-16}$
					Q^1	0·715	0·650	0·779	21·8	$<2 \times 10^{-16}$	$<2 \times 10^{-16}$
					Age$_b$ * Q^1	0·016	0·012	0·020	7·3	1×10^{-12}	$2{\cdot}3 \times 10^{-12}$
					$Q^2 = 0$	2·275	1·134	3·417	3·9	$1 \times 10^{-4+}$	$1{\cdot}9 \times 10^{-4}$
					$Q^2 = 4$	-0·043	-0·888	0·803	-0·1	$0{\cdot}921^{+}$	0·921
					SEQ^1	1·120	0·536	1·705	3·8	2×10^{-4}	$2{\cdot}5 \times 10^{-4}$
					Sex = male	-0·027	-0·251	0·197	-0·2	0·813	0·908
					Cohort = TRACK-HD	-0·510	-0·763	-0·258	-4·0	8×10^{-5}	$1{\cdot}3 \times 10^{-4}$
TMSQ[FL]Q[2]	*Sqrt(TMS$_b$) ~ Age$_b$ + Q^{FL} + Age$_b$ * Q^{FL} + Q^2 + SEQ^1 + Sex + Cohort*	0·578	0·575	$<2 \times 10^{-16}$	intercept	4·190	4·014	4·366	46·7	$<2 \times 10^{-16}$	$<2 \times 10^{-16}$
					Age$_b$	0·173	0·162	0·185	29·1	$<2 \times 10^{-16}$	$<2 \times 10^{-16}$
					Q^{FL}	0·712	0·648	0·776	21·8	$<2 \times 10^{-16}$	$<2 \times 10^{-16}$
					Age$_b$ * Q^{FL}	0·016	0·011	0·020	7·2	1×10^{-12}	$2{\cdot}7 \times 10^{-12}$
					$Q^2 = 0$	3·702	2·545	4·860	6·3	$<1 \times 10^{-5+}$	$1{\cdot}9 \times 10^{-5}$
					$Q^2 = 4$	-0·785	-1·640	0·070	-1·8	$0{\cdot}071^{+}$	0·084
					SEQ^1	1·100	0·515	1·684	3·7	2×10^{-4}	$3{\cdot}2 \times 10^{-4}$
					Sex = male	-0·026	-0·249	0·198	-0·2	0·823	0·908
					Cohort = TRACK-HD	-0·506	-0·759	-0·253	-3·9	9×10^{-5}	$1{\cdot}4 \times 10^{-4}$

Table S7: Genetic associations between candidate SNPs and the somatic expansion scores of the *HTT* CAG repeat. SNPs are ordered by decreasing *p*-value of their association with somatic expansion scores in the discovery TRACK-HD cohort. Chr: chromosome. A1: minor allele. N: number of allele observations. MAF: Minor allele frequency. β: regression coefficient. previous β: β obtained in previous genome-wide association studies: a, GeM-HD β (years/minor allele) with motor onset of HD as reported in Bettencourt *et al.*[15] Supplementary table 4; b, GeM-HD β (years/minor allele) with motor onset of HD as reported in GeM-HD[16] table 1; c, β with TRACK-HD progression score.[4] *t*: *t*-statistic *p*: unadjusted *p*-value. *pFDR*: *p*-value adjusted for multiple testing using the Benjamini-Hochberg false discovery rate correction.[34] *z*: overall *z*-statistic. *Note that in a preliminary analysis using a slightly larger TRACK-HD cohort including four participants with 39 pure CAG repeats ($Q^1 = 39$) and six non-Caucasians, the association between somatic expansion score and rs20579 in *LIG1* was *p* = 0·072, and rs20579 was thus selected for replication in Enroll-HD. Conversely, in the same preliminary analyses rs11061229 had a *p*-value > 0.10 and thus was not selected for replication in Enroll-HD.

SNP ID	Chr	Gene	A1	A2	previous *β*	TRACK-HD						Enroll-HD						Meta-analysis		
						N	MAF	*β*	*t*	*p*	*pFDR*	N	MAF	*β*	*t*	*p*	*pFDR*	*z*	*p*	*pFDR*
rs2140734	15	*MTMR10*	G	T	1·4[b]	404	0·339	0·060	2·969	0·003	0·034									
rs3512	15	*FAN1*	C	G	1·33[a]	404	0·339	0·060	2·969	0·003	0·034	1062	0·326	0·050	4·019	6·7 x 10^{-5}	4·0 x 10^{-4}	4·933	8·1 x 10^{-7}	4·8 x 10^{-6}
rs175080	14	*MLH3*	A	G	-0·43[a]	404	0·448	-0·053	-2·938	0·004	0·034	1048	0·442	-0·029	-2·495	0·013	0·026	-3·644	2·7 x 10^{-4}	8·0 x 10^{-4}
rs147804330	2	*RP11-481J13·1*	A	G	-1·6[b]	402	0·052	-0·107	-2·588	0·010	0·073	1026	0·065	0·003	0·110	0·912	0·912	-1·267	0·205	0·246
rs1382539	5	*MSH3*	A	G	-0·54[c]	404	0·265	-0·045	-2·386	0·018	0·101	1062	0·277	-0·023	-1·771	0·077	0·116	-2·746	0·006	0·009
rs144287831	3	*MLH1*	C	T	0·9[b]	402	0·313	-0·033	-1·701	0·091	0·314									
rs1799977	3	*MLH1*	G	A	0·85[a]	404	0·312	-0·032	-1·686	0·093	0·314	1062	0·304	-0·034	-2·630	0·009	0·026	-3·111	0·002	0·004
rs11061229*	12	*P2_RNA*	C	G	-1·7[b]	378	0·098	0·051	1·675	0·096	0·314									
rs72810940	2	-	A	G	2·4[b]	398	0·023	-0·099	-1·609	0·109	0·314									
rs20579*	19	*LIG1*	A	G	0·77[a]	404	0·151	-0·042	-1·597	0·112	0·314	1062	0·133	-0·002	-0·115	0·908	0·912	-0·932	0·351	0·351
rs72734283	14	*MLH3*	G	A	0·86[a]	404	0·097	0·035	1·179	0·240	0·610									
rs1805323	7	*PMS2*	T	G	-0·95[a]	404	0·037	0·051	1·052	0·294	0·657									
rs3735721	8	*RRM2B*	G	A	-1·53[a]	404	0·067	0·034	1·002	0·318	0·657									
rs61752302	8	*UBR5*	T	C	-1·67[a]	404	0·025	-0·057	-0·960	0·338	0·657									
rs6151792	5	*MSH3*	T	C	-1·05[a]	404	0·099	0·029	0·933	0·352	0·657									
rs115109737	5	*MSH3*	A	G	-1·29[a]	404	0·057	0·028	0·737	0·462	0·775									
rs114136100	15	*FAN1*	T	C	-5·07[a]	404	0·010	0·066	0·717	0·475	0·775									
rs261453	13	-	A	C	-1·3[b]	404	0·087	0·021	0·679	0·498	0·775									
rs4150407	2	*ERCC3*	C	T	0·58[a]	400	0·405	0·008	0·417	0·677	0·835									
rs11133929	5	-	C	T	1·5[b]	398	0·093	-0·013	-0·410	0·683	0·835									
rs1037699	8	*RRM2B*	T	C	-1·57[a]	402	0·097	0·010	0·349	0·727	0·835									
rs1037700	8	*RRM2B*	C	G	-1·54[a]	402	0·097	0·010	0·349	0·727	0·835									
rs5893603	8	*RRM2B*	CG	C	-1·55[a]	402	0·097	0·010	0·349	0·727	0·835									
rs5742933	2	*PMS1*	C	G	-0·70[a]	404	0·183	0·008	0·332	0·740	0·835									
rs12531179	7	*PMS2*	T	C	0·94[a]	400	0·150	0·007	0·278	0·781	0·835									
rs16869352	8	*UBR5*	C	T	-1·53[a]	404	0·089	0·008	0·258	0·797	0·835									
rs1800937	2	*MSH6*	T	C	0·82[a]	404	0·079	-0·007	-0·228	0·820	0·835									
rs71636247	5	*MSH3*	G	A	-1·40[a]	390	0·031	-0·011	-0·209	0·835	0·835									

Tables S8A-D: Human tissue-specific expression quantitative trait data for DNA repair gene SNPs. For each SNP yielding an association with somatic expansion scores, we queried the Gene-Tissue Expression database (https://gtexportal·org/) to identify potential human eQTL effects. For each SNP we show the normalised effect size on gene expression, the raw *p*-value for an association between the SNP and tissue-specific gene expression levels, and the false discovery rate corrected *p*-value (*pFDR*).[34] Tissues with significantly decreased gene expression associated with the minor allele are indicated in red, and tissues with significantly increased gene expression associated with the minor allele are indicated in green.

Table S8A: *MSH3*

**Gene Symbol**	**Gencode Id**	**Variant Id**	**SNP**
MSH3	ENSG00000113318.9	5_79952154_G_A_b37	rs1382539
**Tissue**	**Normalised effect size**	***p*-value**	***pFDR***
Thyroid	-0·49	1·20E-28	6·00E-27
Skin - Sun Exposed (Lower leg)	-0·41	9·90E-25	2·48E-23
Artery - Tibial	-0·49	2·70E-24	4·50E-23
Skin - Not Sun Exposed (Suprapubic)	-0·45	4·40E-24	5·50E-23
Adipose - Subcutaneous	-0·39	8·30E-21	6·14E-20
Nerve - Tibial	-0·48	8·60E-21	6·14E-20
Nerve - Tibial	-0·48	8·60E-21	6·14E-20
Whole Blood	-0·41	3·00E-19	1·88E-18
Heart - Atrial Appendage	-0·41	1·20E-14	6·67E-14
Artery - Aorta	-0·51	2·80E-14	1·40E-13
Adipose - Visceral (Omentum)	-0·33	2·10E-11	9·55E-11
Pancreas	-0·44	7·60E-11	3·17E-10
Adrenal Gland	-0·44	4·90E-09	1·82E-08
Colon - Sigmoid	-0·36	5·10E-09	1·82E-08
Breast - Mammary Tissue	-0·36	3·40E-08	1·13E-07
Colon - Transverse	-0·31	8·60E-08	2·69E-07
Pituitary	-0·42	1·50E-07	4·41E-07
Esophagus - Muscularis	-0·26	4·20E-07	1·17E-06
Esophagus - Mucosa	-0·19	0·0000029	7·63E-06
Artery - Coronary	-0·33	0·000016	4·00E-05
Stomach	-0·3	0·000035	8·33E-05
Brain - Nucleus accumbens (basal ganglia)	-0·27	0·000075	0·000170455
Brain - Cortex	-0·37	0·00014	0·000304348
Heart - Left Ventricle	-0·14	0·00015	0·0003125
Lung	-0·16	0·00023	0·00046
Brain - Amygdala	-0·48	3·50E-04	0·000648148
Brain - Amygdala	-0·48	0·00035	0·000648148
Brain - Caudate (basal ganglia)	-0·25	4·20E-04	0·000724138
Brain - Caudate (basal ganglia)	-0·25	0·00042	0·000724138
Brain - Anterior cingulate cortex (BA24)	-0·28	7·00E-04	0·001129032
Brain - Anterior cingulate cortex (BA24)	-0·28	0·0007	0·001129032
Vagina	-0·27	0·00078	0·00121875
Ovary	-0·32	0·0014	0·002121212
Brain - Hippocampus	-0·3	0·0019	0·002794118
Brain - Putamen (basal ganglia)	-0·28	0·0032	0·004571429
Prostate	-0·24	0·0053	0·007361111
Minor Salivary Gland	-0·3	0·0075	0·01013514
Brain - Spinal cord (cervical c-1)	-0·39	0·011	0·01447368
Brain - Hypothalamus	-0·21	0·021	0·02692308
Liver	0·16	0·024	0·03
Uterus	-0·24	0·028	0·03414634
Brain - Substantia nigra	-0·24	0·06	0·07142857
Brain - Cerebellum	-0·14	0·08	0·09302326
Spleen	-0·16	0·085	0·09659091
Muscle - Skeletal	-0·058	0·09	0·1
Brain - Frontal Cortex (BA9)	-0·13	0·14	0·1521739
Brain - Cerebellar Hemisphere	-0·11	1·70E-01	0·1770833
Brain - Cerebellar Hemisphere	-0·11	0·17	0·1770833
Small Intestine - Terminal Ileum	-0·084	0·44	0·4489796
Testis	0·037	0·55	0·55

Table S8B: *MLH1*

Gene Symbol	Gencode Id	Variant Id	SNP
MLH1	ENSG00000076242.10	3_37053568_A_G_b37	rs1799977
**Tissue**	**Normalised effect size**	***p*-value**	***pFDR***
Muscle - Skeletal	0·18	2·90E-09	1·45E-07
Testis	0·11	0·035	0·6956522
Adipose - Visceral (Omentum)	-0·096	0·043	0·6956522
Brain - Spinal cord (cervical c-1)	0·18	0·063	0·6956522
Pancreas	-0·12	0·076	0·6956522
Uterus	-0·21	0·088	0·6956522
Artery - Tibial	0·054	0·11	0·6956522
Esophagus - Muscularis	0·055	0·14	0·6956522
Minor Salivary Gland	0·11	0·16	0·6956522
Heart - Left Ventricle	-0·044	0·18	0·6956522
Prostate	0·091	0·19	0·6956522
Whole Blood	-0·043	0·19	0·6956522
Thyroid	0·041	0·2	0·6956522
Ovary	-0·13	0·21	0·6956522
Colon - Sigmoid	0·062	0·24	0·6956522
Brain - Anterior cingulate cortex (BA24)	-0·13	0·25	0·6956522
Brain - Anterior cingulate cortex (BA24)	-0·13	0·25	0·6956522
Artery - Aorta	0·059	0·27	0·6956522
Vagina	-0·1	0·27	0·6956522
Brain - Caudate (basal ganglia)	-0·082	0·3	0·6956522
Brain - Caudate (basal ganglia)	-0·082	0·3	0·6956522
Brain - Hypothalamus	-0·09	0·31	0·6956522
Brain - Substantia nigra	0·11	0·32	0·6956522
Brain - Hippocampus	-0·078	0·38	0·7916667
Lung	-0·026	0·41	0·8103448
Adipose - Subcutaneous	-0·034	0·44	0·8103448
Spleen	-0·05	0·44	0·8103448
Liver	-0·042	0·46	0·8103448
Small Intestine - Terminal Ileum	-0·044	0·47	0·8103448
Brain - Frontal Cortex (BA9)	-0·066	0·56	0·9
Brain - Cerebellar Hemisphere	0·04	0·6	0·9
Brain - Cerebellar Hemisphere	0·04	0·6	0·9
Brain - Nucleus accumbens (basal ganglia)	-0·047	0·66	0·9
Artery - Coronary	0·033	0·67	0·9
Pituitary	0·027	0·68	0·9
Adrenal Gland	0·019	0·7	0·9
Stomach	0·022	0·7	0·9
Nerve - Tibial	-0·017	0·72	0·9
Colon - Transverse	-0·014	0·72	0·9
Nerve - Tibial	-0·017	0·72	0·9
Skin - Sun Exposed (Lower leg)	-0·011	0·74	0·902439
Brain - Putamen (basal ganglia)	-0·033	0·76	0·9042553
Brain - Cortex	0·02	0·82	0·9042553
Esophagus - Mucosa	0·0074	0·82	0·9042553
Heart - Atrial Appendage	-0·0078	0·84	0·9042553
Brain - Amygdala	0·023	0·85	0·9042553
Brain - Amygdala	0·023	0·85	0·9042553
Skin - Not Sun Exposed (Suprapubic)	-0·0058	0·88	0·9166667
Brain - Cerebellum	0·0026	0·97	0·98
Breast - Mammary Tissue	0·0013	0·98	0·98

Table S8C: *MLH3*

Gene Symbol	Gencode Id	Variant Id	SNP
MLH3	ENSG00000119684.11	14_75513828_G_A_b37	rs175080
**Tissue**	**Normalised effect size**	***p*-value**	***pFDR***
Whole Blood	-0·21	9·60E-07	**4·80E-05**
Nerve - Tibial	0·19	0·0000075	**0·000125**
Nerve - Tibial	0·19	0·0000075	**0·000125**
Brain - Hippocampus	0·21	0·00016	**0·002**
Testis	-0·15	0·00022	**0·0022**
Muscle - Skeletal	-0·13	0·00045	**0·00375**
Esophagus - Mucosa	-0·10	0·0017	**0·01214286**
Colon - Transverse	0·15	0·0037	**0·023125**
Brain - Nucleus accumbens (basal ganglia)	-0·18	0·0097	0·05388889
Pituitary	0·12	0·011	0·055
Skin - Sun Exposed (Lower leg)	0·076	0·017	0·07727273
Adipose - Visceral (Omentum)	-0·087	0·028	0·1166667
Heart - Left Ventricle	-0·099	0·033	0·1269231
Breast - Mammary Tissue	0·10	0·053	0·18
Colon - Sigmoid	0·14	0·054	0·18
Stomach	-0·072	0·073	0·228125
Artery - Aorta	-0·076	0·083	0·2441176
Artery - Tibial	0·062	0·096	0·2666667
Liver	-0·092	0·17	0·4473684
Small Intestine - Terminal Ileum	0·094	0·19	0·475
Vagina	0·088	0·2	0·4761905
Brain - Frontal Cortex (BA9)	-0·096	0·22	0·5
Thyroid	0·042	0·24	0·5
Brain - Cerebellar Hemisphere	0·073	0·25	0·5
Brain - Cerebellar Hemisphere	0·073	0·25	0·5
Skin - Not Sun Exposed (Suprapubic)	-0·037	0·3	0·5740741
Spleen	-0·078	0·31	0·5740741
Esophagus - Muscularis	-0·042	0·36	0·6282051
Brain - Cortex	-0·065	0·37	0·6282051
Ovary	-0·094	0·38	0·6282051
Uterus	-0·093	0·41	0·6282051
Brain - Caudate (basal ganglia)	-0·055	0·44	0·6282051
Brain - Caudate (basal ganglia)	-0·055	0·44	0·6282051
Pancreas	-0·044	0·46	0·6282051
Brain - Anterior cingulate cortex (BA24)	0·054	0·47	0·6282051
Brain - Anterior cingulate cortex (BA24)	0·054	0·47	0·6282051
Adrenal Gland	-0·035	0·48	0·6282051
Brain - Amygdala	0·053	0·49	0·6282051
Brain - Amygdala	0·053	0·49	0·6282051
Brain - Substantia nigra	-0·048	0·54	0·6707317
Lung	0·019	0·55	0·6707317
Brain - Cerebellum	0·025	0·57	0·6785714
Brain - Hypothalamus	-0·04	0·6	0·6976744
Brain - Spinal cord (cervical c-1)	0·03	0·66	0·75
Brain - Putamen (basal ganglia)	-0·029	0·72	0·7826087
Minor Salivary Gland	-0·031	0·72	0·7826087
Heart - Atrial Appendage	-0·01	0·85	0·9042553
Artery - Coronary	-0·0081	0·92	0·9583333
Adipose - Subcutaneous	0·0031	0·94	0·9591837
Prostate	0·0014	0·99	0·99

Table S8D: *FAN1*

Gene Symbol	Gencode Id	Variant Id	SNP
FAN1	ENSG00000198690.5	15_31235005_G_C_b37	rs3512
**Tissue**	**Normalised effect size**	***p*-value**	***pFDR***
Brain - Cortex	0·35	4·20E-07	1·89E-05
Adipose - Subcutaneous	0·19	0·0000025	5·63E-05
Brain - Anterior cingulate cortex (BA24)	0·25	0·000088	0·00132
Skin - Sun Exposed (Lower leg)	0·1	0·00021	0·0023625
Brain - Nucleus accumbens (basal ganglia)	0·16	0·0016	0·0135
Whole Blood	0·13	0·0018	0·0135
Heart - Left Ventricle	-0·087	0·0039	0·02507143
Brain - Frontal Cortex (BA9)	0·17	0·0085	0·0478125
Brain - Hippocampus	0·15	0·011	0·055
Breast - Mammary Tissue	0·12	0·019	0·08181818
Ovary	-0·22	0·02	0·08181818
Brain - Putamen (basal ganglia)	0·13	0·027	0·1003846
Pancreas	-0·13	0·029	0·1003846
Artery - Tibial	0·083	0·044	0·1414286
Heart - Atrial Appendage	-0·08	0·065	0·195
Lung	0·067	0·071	0·1996875
Brain - Substantia nigra	0·13	0·092	0·2435294
Brain - Spinal cord (cervical c-1)	0·11	0·12	0·3
Pituitary	-0·083	0·13	0·3078947
Artery - Coronary	0·1	0·15	0·3375
Liver	-0·069	0·18	0·3857143
Esophagus - Mucosa	-0·038	0·23	0·4704545
Brain - Amygdala	-0·085	0·29	0·5673913
Brain - Cerebellum	0·076	0·31	0·58125
Testis	-0·047	0·35	0·63
Nerve - Tibial	0·033	0·37	0·6362069
Colon - Transverse	-0·032	0·39	0·6362069
Brain - Cerebellar Hemisphere	0·046	0·41	0·6362069
Brain - Hypothalamus	0·043	0·41	0·6362069
Spleen	0·048	0·44	0·6387097
Stomach	-0·035	0·44	0·6387097
Skin - Not Sun Exposed (Suprapubic)	0·023	0·48	0·6545455
Uterus	0·08	0·48	0·6545455
Thyroid	0·025	0·52	0·6875
Esophagus - Muscularis	0·019	0·55	0·6875
Small Intestine - Terminal Ileum	-0·04	0·55	0·6875
Colon - Sigmoid	-0·017	0·64	0·7783784
Muscle - Skeletal	-0·013	0·66	0·7815789
Minor Salivary Gland	-0·034	0·68	0·7846154
Brain - Caudate (basal ganglia)	0·021	0·74	0·8325
Artery - Aorta	0·014	0·79	0·8571429
Adrenal Gland	0·013	0·8	0·8571429
Adipose - Visceral (Omentum)	0·0072	0·85	0·8895349
Prostate	0·01	0·91	0·9306818
Vagina	-0·0022	0·98	0·98

Supplementary figures

Figure S1: Comparison of pure CAG repeat lengths determined by deep-sequencing (Q^1) and estimated by fragment-length analyses (Q^{FL}). A) Scatter plot of pure CAG length determined via direct sequencing versus estimated CAG length determined via standard fragment-length analysis. Fragment-length analysis consistently mis-sized all disease-associated atypical alleles ($Q^1 \geq 40$) with the CAACAG deletion ($Q^2 = 0$, $n = 7$) as two repeats smaller than they actually are. Surprisingly, fragment-length analysis consistently mis-sized all atypical alleles with the CAACAG duplication ($Q^2 = 4$, $n = 13$) as only one repeat larger than they actually are, rather than the expected two repeat difference. Similarly, fragment-length analysis unexpectedly correctly sized the one atypical allele with the $(CAA)_2(CAG)_1$ complement of glutamine encoding codons downstream of the pure CAG tract ($Q^2 = 3$), rather than the expected one repeat difference. Only one typical allele ($Q^2 = 2$) was differentially sized by fragment-length analysis as 49 rather than 48 CAG repeats. Symbol size is proportional to the number of observations as shown. **B) Location of PCR primers used for fragment-length analysis.** The position of the PCR primers used in the standard diagnostic test[7] are indicated. Note that the reverse primer, HD3, spans the polymorphic region between the pure CAG and CCG tracts. **C/D/E/F)** Schematic representation of the common *HTT* CAG structures (top) and the estimates of CAG length derived by standard fragment-length analysis (Q^{FL}, lower) for a typical allele ($Q^2 = 2$) **(C)**, an atypical allele with deletion of the CAACAG cassette ($Q^2 = 0$) **(D)**, an atypical allele with the $(CAA)_2(CAG)_1$ complement of glutamine encoding codons downstream of the pure CAG tract ($Q^2 = 3$) **(E)**, and an atypical allele with duplication of the CAACAG cassette ($Q^2 = 4$) **(F)**. Note that fragment-length analysis under-estimates the pure CAG length (Q^1) by two repeats when the CAACAG cassette is deleted ($Q^1 = Q^{FL} + 2$, when $Q^2 = 0$) **(D)**. Note also, that fragment-length analysis unexpectedly correctly-estimated the pure CAG length (Q^1) for an atypical allele with the $(CAA)_2(CAG)_1$ complement of glutamine encoding codons downstream of the pure CAG tract ($Q^2 = 3$) **(E)**, and that fragment-length analysis over-estimates the pure CAG length (Q^1) by one repeat when the CAACAG cassette is duplicated ($Q^1 = Q^{FL} - 1$, when $Q^2 = 4$) **(F)**, presumably due to mis-priming of HD3.

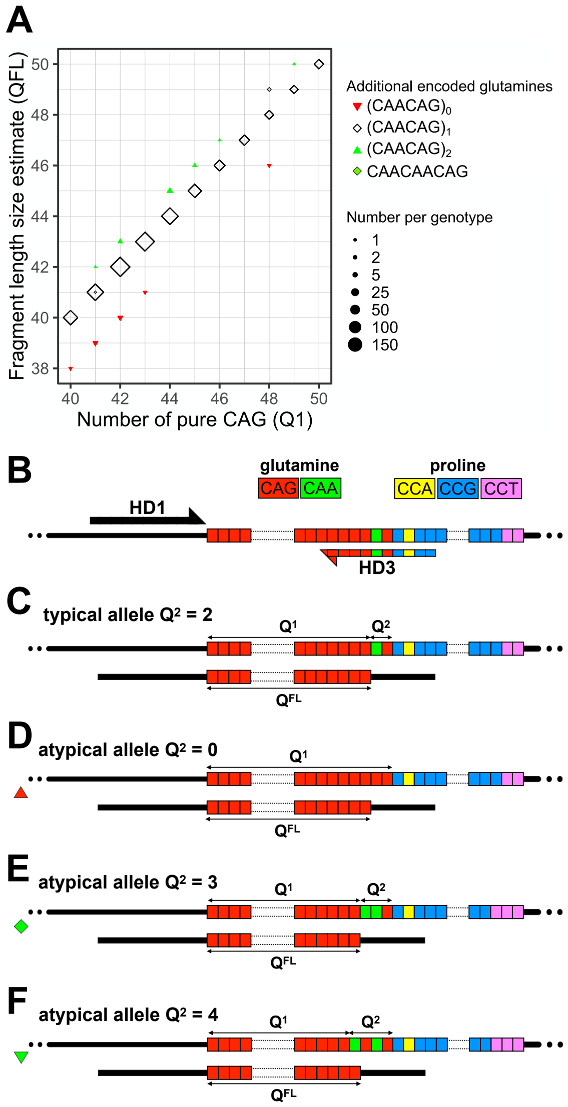

Figure S2: Read depth distributions for CAG repeat length support a somatic origin for repeat length gains. The graphs show the proportion of DNA sequence reads mapped to each CAG repeat length reference for progenitor alleles of different lengths derived from sequencing of PCR amplification of either single molecules or bulk blood DNA samples (20 ng) from either younger or older carriers of disease-associated alleles. Note that all non-progenitor (Q^1) reads for the single-molecule amplified products must represent PCR slippage errors and that these are almost exclusively biased toward loss of repeats and that repeat length gains are virtually absent (see also figure S3). Note further that frequent repeat length gains are observed in the bulk DNA analyses (see also figure S3), and that these are much more frequent in the older individuals.

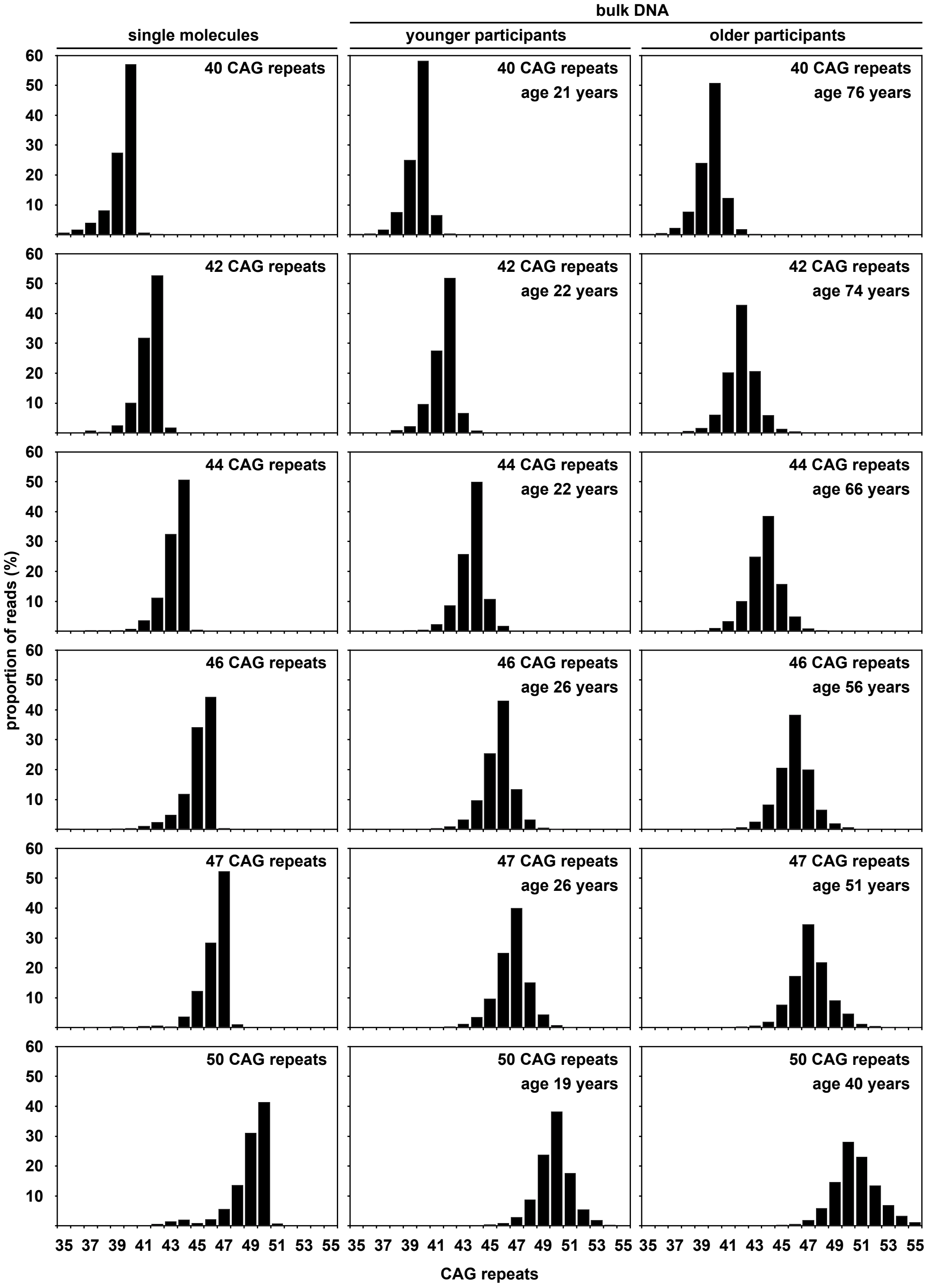

Figure S3: Read depth distributions for CAG repeat length support a somatic origin for repeat length gains. A) The proportion of DNA sequence reads derived from larger alleles is much higher in bulk DNA than in alleles amplified from single molecules. The graph shows the proportion of reads mapped to CAG repeat length references greater (n + 1) than the progenitor alleles (n = Q^1) of different lengths derived from PCR amplification of either single molecules (crosses) or bulk blood DNA samples (open diamonds). Note that the fraction of expansions observed in bulk DNA sample is much greater than that caused by PCR slippage in single molecules. **B) The proportion of DNA sequence reads derived from smaller alleles is similar in single molecules and in bulk DNA.** The graph shows the proportion of reads mapped to CAG repeat length references smaller (n - 1) than the progenitor alleles (n = Q^1) of different lengths derived from PCR amplification of either single molecules (crosses) or bulk blood DNA samples (open diamonds). Note that the fraction of contractions observed in bulk DNA samples is similar to that caused by PCR slippage in single molecules. **C) Interpretation of non-progenitor sequence reads in bulk DNA analyses.** The data presented in figures S2 and S3A and B support a model in which the vast majority of reads shorter than the progenitor (n = Q^1) are PCR Taq polymerase slippage errors (n-1, n-2 *etc.*) and that the vast majority of reads longer than the progenitor allele represent genuine somatic expansions (n+1, n+2 *etc.*).

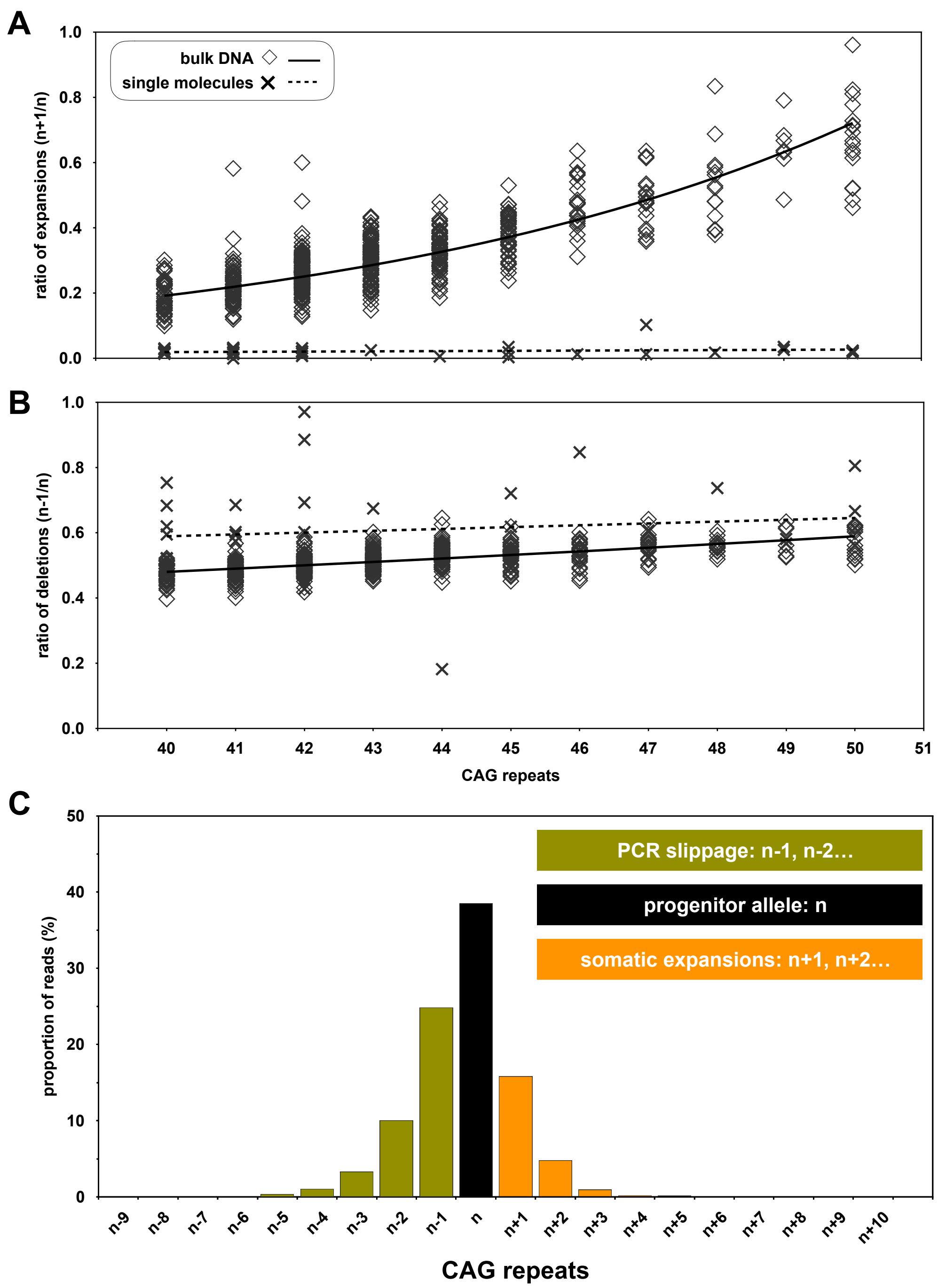

Figure S4: Relative toxicities for alleles dependent either on total encoded glutamine or pure CAG length. Alleles with the same number of total encoded glutamines (Q^T) (upper), but differing in the number of CAACAG cassettes (Q^2), have a degree of somatic expansion and disease severity that is best reflected by the number of pure CAG repeats (Q^1) *i.e.* for the same number of encoded glutamines, more somatic expansions associate with worse disease outcomes. Alleles with the same number of pure CAG repeats (Q^1) (lower), but differing in the number of CAACAG cassettes (Q^2), have a similar degree of somatic expansion. Alleles with the same number of pure CAG repeats (Q^1) (lower), but with more CAACAG cassettes (Q^2) should generate proteins with longer toxic polyglutamine (pQ) tracts. Longer toxic polyglutamine tracts might be expected to result in worse outcomes. However, our data suggest that after correcting for pure CAG length (Q^1), people with fewer total encoded glutamines have worse outcomes. Repeat codons are depicted: CAG glutamine codons as red boxes; and, CAA glutamine codons as green boxes. The direction of the arrows and brighter red indicate: more somatic expansions; worse outcomes; and, greater expected polyglutamine (polyQ) toxicity.

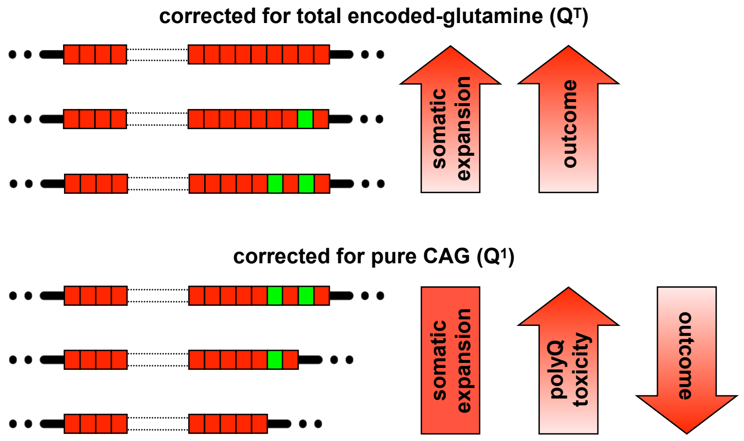

Figure S5: Association of pure CAG repeat length (Q^1), fragment length estimated CAG (Q^{FL}) and total encoded-glutamine length (Q^T) with the number of additional glutamine codons (Q^2) on disease-associated chromosomes. The histograms show the relationship between pure CAG repeat length (Q^1, left), fragment length estimated CAG (Q^{FL}, middle) and total encoded-glutamine length (Q^T, right), with the number of downstream glutamine codons (Q^2) on disease-associated chromosomes ($40 \leq Q^1 \leq 50$) in the TRACK-HD and Enroll-HD cohorts. A linear regression analysis between pure CAG repeat length (Q^1) and the number of additional glutamine codons (Q^2) revealed: $r = 0.05$, adjusted $r^2 = 0{\cdot}001$, $p = 0{\cdot}17$. A linear regression analysis between pure CAG repeat length (Q^{FL}) and the number of additional glutamine codons (Q^2) revealed: $r = 0.14$, adjusted $r^2 = 0{\cdot}019$, $p = 8{\cdot}8 \times 10^{-5}$. A linear regression analysis between total encoded-glutamine length (Q^T) and the number of downstream glutamine codons (Q^2) revealed: $r = 0.19$, adjusted $r^2 = 0{\cdot}034$, $p = 2{\cdot}3 \times 10^{-7}$.

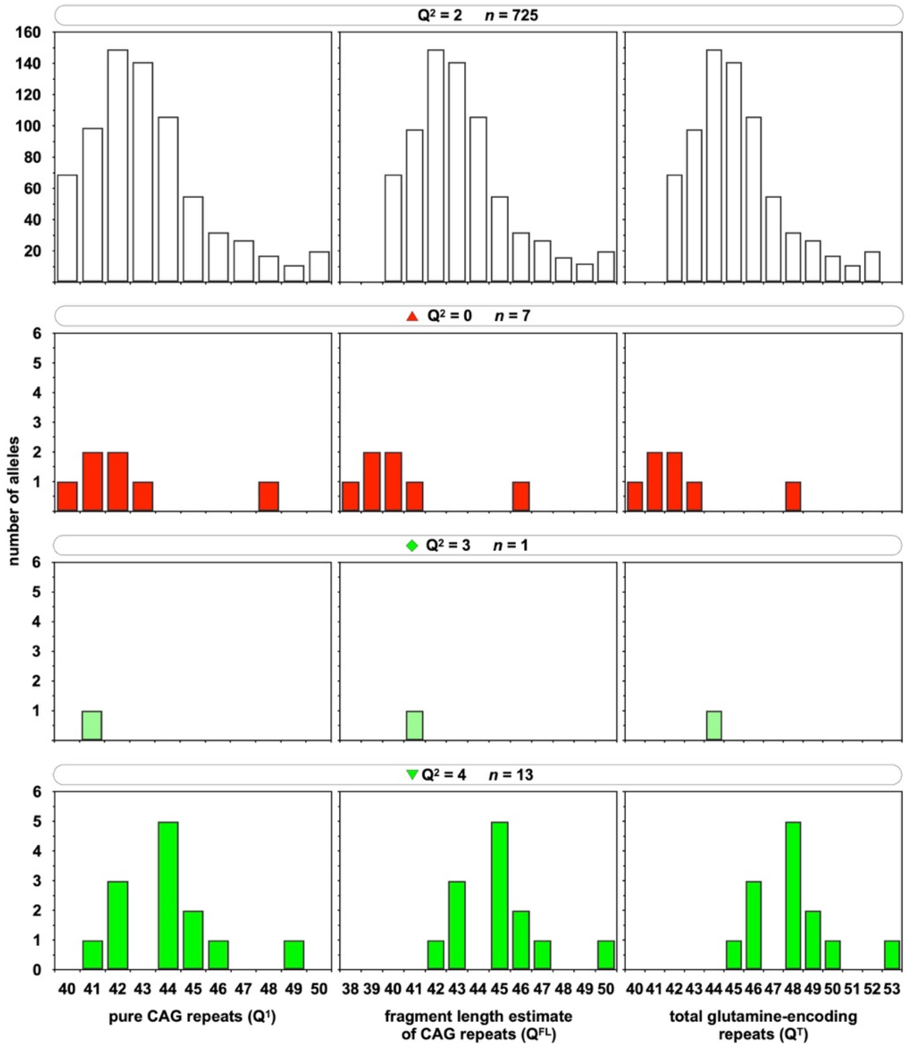

Figure S6: Blood and brain dynamics of CAG repeats dependent on CAG structures and SNP genotypes. A) Blood and brain dynamics of CAG repeats dependent on CAG structures. We have shown that alleles with the same number of total glutamines (Q^T), but with fewer CAACAG cassettes (Q^2) expand more rapidly with time in blood DNA. We predict that expansions in brain are larger, but mirror the pattern observed in blood DNA and that this difference in mutational dynamics explains variable disease outcomes *i.e.* alleles with the same number of total glutamines (Q^T), but with fewer CAACAG cassettes (Q^2), expand more rapidly with time in brain causing worse outcomes. **B) Blood and brain dynamics of CAG repeats dependent on *MSH3* rs1382539 SNP genotypes.** Individuals homozygous for the rs1382539 SNP G allele have more somatic expansions in blood DNA. rs1382539 is an expression quantitative trait locus (eQTL) and the G allele is associated with higher gene expression in multiple tissues, including vulnerable regions of the HD brain such as the caudate and cortex (table S8A, appendix). Mouse model data reveal *Msh3* is essential to drive somatic CAG repeat expansions.[35-37] We thus hypothesise that the rs1382539 G allele drives higher *MSH3* expression in blood stem cells and that resultant higher enzymatic activity increases the rate of somatic expansion in blood DNA, and that this effect is mirrored in critical brain regions leading to worse HD outcomes. **C) Blood and brain dynamics of CAG repeats dependent on *MLH3* rs175080 SNP genotypes.** Individuals homozygous for the rs175080 SNP A allele have fewer somatic expansions in blood DNA. rs175080 is an eQTL with the A allele associated with lower gene expression in some tissues (*e.g.* whole blood), and higher gene expression in other tissues (*e.g.* hippocampus) (table S8C, appendix). Mouse model data reveal *Mlh3* is essential to drive somatic CAG repeat expansions.[38] We thus hypothesise that the rs175080 A allele drives lower *MLH3* expression in blood stem cells and that resultant lower enzymatic activity decreases the rate of somatic expansion in blood DNA. We hypothesise that the gene expression effects are reversed in critical brain regions leading to worse HD outcomes with the rs175080 A allele. Repeat codons are depicted: CAG glutamine codons as red boxes; and, CAA glutamine codons as green boxes. The direction of the arrows and brighter red indicate: worse outcomes; and, higher gene expression.

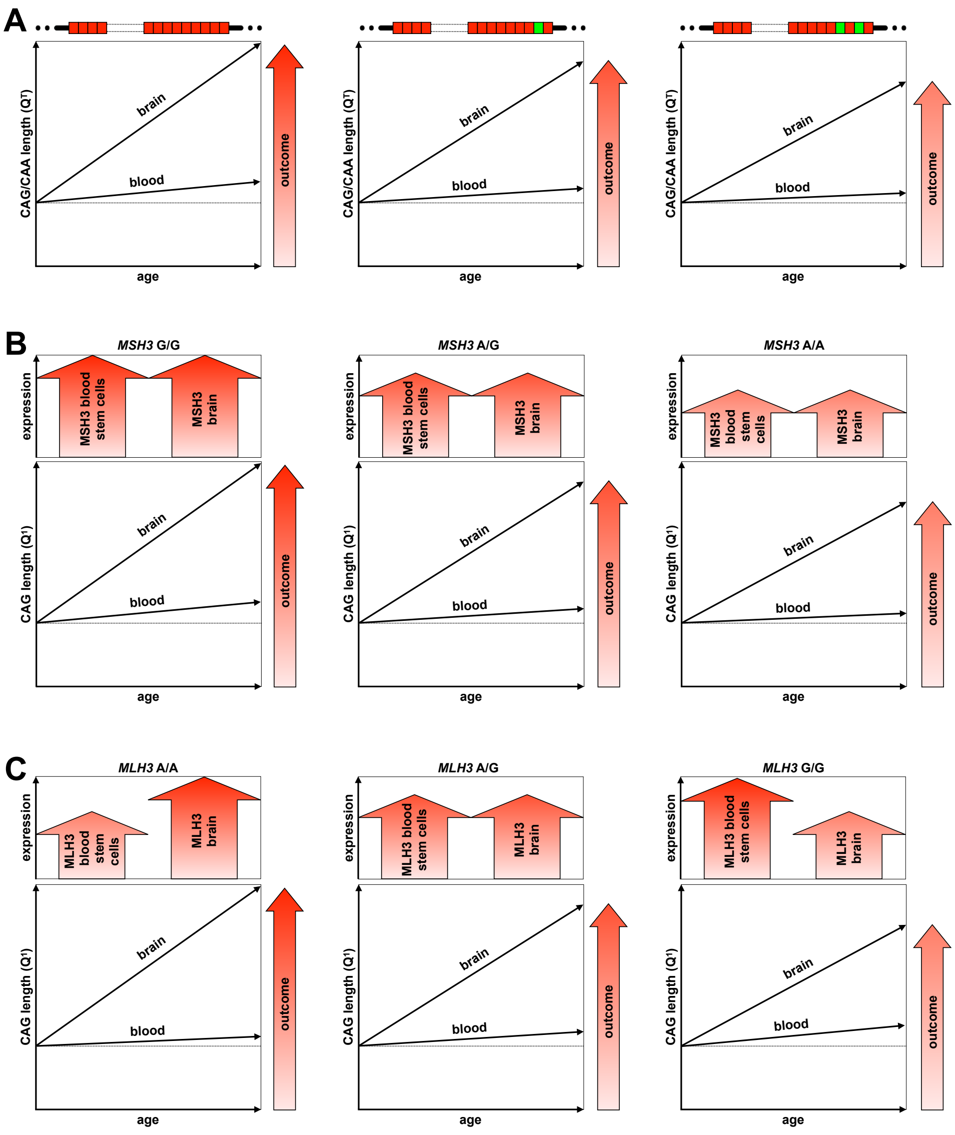

IV. References for supplementary material

1 Tabrizi SJ, Langbehn DR, Leavitt BR, et al. Biological and clinical manifestations of Huntington's disease in the longitudinal TRACK-HD study: cross-sectional analysis of baseline data. *Lancet Neurol* 2009; **8:** 791–801.

2 Huntington Study Group. Unified Huntington's Disease Rating Scale: reliability and consistency. *Mov Disord* 1996; **11:** 136–42.

3 Penney JB, Jr., Vonsattel JP, MacDonald ME, Gusella JF, Myers RH. CAG repeat number governs the development rate of pathology in Huntington's disease. *Ann Neurol* 1997; **41:** 689–92.

4 Hensman Moss DJ, Pardinas AF, Langbehn D, et al. Identification of genetic variants associated with Huntington's disease progression: a genome-wide association study. *Lancet Neurol* 2017; **16:** 701–11.

5 Perlis RH, Smoller JW, Mysore J, et al. Prevalence of incompletely penetrant Huntington's disease alleles among individuals with major depressive disorder. *Am J Psychiatry* 2010; **167:** 574–79.

6 Warner JP, Barron LH, Brock DJ. A new polymerase chain reaction (PCR) assay for the trinucleotide repeat that is unstable and expanded on Huntington's disease chromosomes. *Mol Cell Probes* 1993; **7:** 235–39.

7 Losekoot M, van Belzen MJ, Seneca S, et al. EMQN/CMGS best practice guidelines for the molecular genetic testing of Huntington disease. *Eur J Hum Genet* 2013; **21:** 480–86.

8 Landwehrmeyer GB, Fitzer-Attas CJ, Giuliano JD, et al. Data analytics from Enroll-HD, a global clinical research platform for Huntington's disease. *Mov Disord Clin Pract* 2017; **4:** 212—24.

9 Langbehn DR, Brinkman RR, Falush D, Paulsen JS, Hayden MR, International Huntington's Disease Collaborative Group. A new model for prediction of the age of onset and penetrance for Huntington's disease based on CAG length. *Clin Genet* 2004; **65:** 267–77.

10 Ciosi M, Cumming SA, Alshammari AM, et al. Library preparation and MiSeq sequencing for the genotyping-by-sequencing of the Huntington disease *HTT* exon one trinucleotide repeat and the quantification of somatic mosaicism. *Protocol Exchange* 2018; **DOI: 10.1038/protex.2018.089**.

11 Martin M. Cutadapt removes adapter sequences from high-throughput sequencing reads. *EMBnetjournal* 2011; **17:** 10–12.

12 Yu S, Fimmel A, Fung D, Trent RJ. Polymorphisms in the CAG repeat--a source of error in Huntington disease DNA testing. *Clin Genet* 2000; **58:** 469–72.

13 Milne I, Stephen G, Bayer M, et al. Using Tablet for visual exploration of second-generation sequencing data. *Brief Bioinform* 2013; **14:** 193–202.

14 Gomes-Pereira M, Bidichandani SI, Monckton DG. Analysis of unstable triplet repeats using small-pool polymerase chain reaction. *Methods Mol Biol* 2004; **277:** 61–76.

15 Bettencourt C, Hensman-Moss D, Flower M, et al. DNA repair pathways underlie a common genetic mechanism modulating onset in polyglutamine diseases. *Ann Neurol* 2016; **79:** 983–90.

16 The Genetic Modifiers of Huntington's Disease Consortium. Identification of genetic factors that modify clinical onset of Huntington's disease. *Cell* 2015; **162:** 516–26.

17 Lenth R. emmeans: estimated marginal means, aka least-squares means. R package. version 1.3.4, 2019. https://cran.r-project.org/package=emmeans

18 Therneau T. A package for survival analysis in S. R package version 2.38, 2015. https://cran.r-project.org/package=survival

19 Therneau TM, Grambsch PM. Modeling survival data: extending the Cox model. New York: Springer, 2000.

20 Kassambara A, Kosinski M. survminer: Drawing Survival Curves using 'ggplot2'. R package. version 0.4.3, 2018. https://cran.r-project.org/package=survminer

21 Canty A, Ripley B. boot: bootstrap R (S-Plus) functions. R package. version 1.3-22, 2019. https://cran.r-project.org/web/package=boot

22 Davison AC, Hinkley DV. Bootstrap methods and their applications. Cambridge: Cambridge University Press, 1997.

23 Bates D, Machler M, Bolker BM, Walker SC. Fitting linear mixed-effects models using lme4. *J Stat Softw* 2015; **67:** 1–48.

24 Pecheux C, Mouret JF, Durr A, et al. Sequence analysis of the CCG polymorphic region adjacent to the CAG triplet repeat of the HD gene in normal and HD chromosomes. *J Med Genet* 1995; **32:** 399–400.

25 Goldberg YP, McMurray CT, Zeisler J, et al. Increased instability of intermediate alleles in families with sporadic Huntington disease compared to similar sized intermediate alleles in the general population. *Hum Mol Genet* 1995; **4:** 1911–18.

26 Gellera C, Meoni C, Castellotti B, et al. Errors in Huntington disease diagnostic test caused by trinucleotide deletion in the *IT15* gene. *Am J Hum Genet* 1996; **59:** 475–77.

27 Chong SS, Almqvist E, Telenius H, et al. Contribution of DNA sequence and CAG size to mutation frequencies of intermediate alleles for Huntington disease: evidence from single sperm analyses. *Hum Mol Genet* 1997; **6:** 301–09.

28 Margolis RL, Stine OC, Callahan C, et al. Two novel single-base-pair substitutions adjacent to the CAG repeat in the huntington disease gene (*IT15*): implications for diagnostic testing. *Am J Hum Genet* 1999; **64:** 323–26.

29 Kelly TE, Allinson P, McGlennen RC, Baker J, Bao Y. Expansion of a 27 CAG repeat allele into a symptomatic Huntington disease-producing allele. *Am J Med Genet* 1999; **87:** 91–92.
30 Williams LC, Hegde MR, Nagappan R, et al. Null alleles at the Huntington disease locus: implications for diagnostics and CAG repeat instability. *Genetic Testing* 2000; **4:** 55–60.
31 Norremolle A, Budtz-Jorgensen E, Fenger K, Nielsen JE, Sorensen SA, Hasholt L. 4p16.3 haplotype modifying age at onset of Huntington disease. *Clin Genet* 2009; **75:** 244–50.
32 Houge G, Bruland O, Bjornevoll I, Hayden MR, Semaka A. *De novo* Huntington disease caused by 26-44 CAG repeat expansion on a low-risk haplotype. *Neurology* 2013; **81:** 1099–100.
33 Becanovic K, Norremolle A, Neal SJ, et al. A SNP in the *HTT* promoter alters NF-kappaB binding and is a bidirectional genetic modifier of Huntington disease. *Nat Neurosci* 2015; **18:** 807–16.
34 Benjamini Y, Hochberg Y. Controlling the false discovery rate: a practical and powerful approach to multiple testing. *J R Stat Soc B* 1995; **57:** 289–300.
35 Tome S, Manley K, Simard JP, et al. MSH3 polymorphisms and protein levels affect CAG repeat instability in Huntington's disease mice. *PLoS Genet* 2013; **9:** e1003280.
36 van den Broek WJ, Nelen MR, Wansink DG, et al. Somatic expansion behaviour of the $(CTG)_{(n)}$ repeat in myotonic dystrophy knock-in mice is differentially affected by Msh3 and Msh6 mismatch-repair proteins. *Hum Mol Genet* 2002; **11:** 191–98.
37 Dragileva E, Hendricks A, Teed A, et al. Intergenerational and striatal CAG repeat instability in Huntington's disease knock-in mice involve different DNA repair genes. *Neurobiol Dis* 2009; **33:** 37–47.
38 Pinto RM, Dragileva E, Kirby A, et al. Mismatch repair genes *Mlh1* and *Mlh3* modify CAG instability in Huntington's disease mice: genome-wide and candidate approaches. *PLoS Genet* 2013; **9:** e1003930.

V· Investigator lists

V·1. TRACK-HD investigator list

Name	Institution
‘t Hart, E.P.	Leiden University Medical Centre, Leiden, Netherlands
Acharya, T.	University of Iowa, Iowa City, IA, USA
Andrews, S.C.	Monash University, Victoria, Australia
Arran, N.	St Mary’s Hospital, Manchester, UK
Axelson, E.	University of Iowa, Iowa City, IA, USA
Bardinet, E.	APHP, Hôpital Salpêtriere, Paris, France
Bechtel, N.	University of Münster, Münster, Germany
Berna, C.	University College London, London, UK
Bohlen, S.	University of Münster, Münster, Germany
Borowsky, B.	CHDI, USA
Callaghan, J.	St Mary’s Hospital, Manchester, UK
Campbell, C.	Indiana University, IN, USA / Monash University, Victoria, Australia
Campbell, M.	Monash University, Victoria, Australia
Cash, D.M.	IXICO, London, UK
Coleman, A.	University of British Columbia, Vancouver, Canada
Craufurd, D.	St Mary’s Hospital, Manchester, UK
Crawford, H.	University College London, London, UK
Dar Santos, R.	University of British Columbia, Vancouver, Canada
Decolongon, J.	University of British Columbia, Vancouver, Canada
Dumas, E.M.	Leiden University Medical Centre, Leiden, Netherlands
Fox, N.C.	University College London, London, UK
Frajman, E.	Monash University, Victoria, Australia
Frost, C.	London School of Hygiene and Tropical Medicine, London, UK
Gibbard, C.	University College London, London, UK
Hicks, S.	University of Oxford, Oxford, UK
Hobbs, N.Z.	University College London, London, UK
Hoffman, A.	Universtiy of Bochum, Bochum, Germany
Jauffret, C.	APHP, Hôpital Salpêtriere, Paris, France
Johnson, H.	University of Iowa, Iowa City, IA, USA
Jones, R.	London School of Hygiene and Tropical Medicine, London, UK
Jurgens, C.	Leiden University Medical Centre, Leiden, Netherlands
Justo, D.	APHP, Hôpital Salpêtriere, Paris, France
Keenan, S.	Imperial College London, London, UK
Kennard, C.	University of Oxford, Oxford, UK
Kraus, P.	Universtiy of Bochum, Bochum, Germany
Labuschagne, I.	Monash University, Victoria, Australia
Lahiri, N.	University College London, London, UK
Landwehrmeyer, B.	Ulm University, Ulm, Germany
Lee, S.	Massachusetts General Hospital, Harvard, MA, USA
Lehericy, S.	APHP, Hôpital Salpêtriere, Paris, France
Malone, I.	University College London, London, UK
Marelli, C.	APHP, Hôpital Salpêtriere, Paris, France
Milchman, C.	Monash University, Victoria, Australia
Monaco, W.	Massachusetts General Hospital, Harvard, MA, USA
Nigaud, K.	APHP, Hôpital Salpêtriere, Paris, France
Ordidge, R.	University College London, London, UK
O'Regan, A.	Monash University, Victoria, Australia
Owen, G.	University College London, London, UK

Patel, A.	University College London, London, UK
Pepple, T.	University College London, London, UK
Pourchot, P.	APHP, Hôpital Salpêtriere, Paris, France
Queller, S.	Indiana University, IN, USA
Read, J.	University College London, London, UK
Reilmann, R.	University of Münster, Münster, Germany
Rosas, H.D.	Massachusetts General Hospital, Harvard, MA, USA
Say, M.J.	University College London, London, UK
Scahill, R.I.	University College London, London, UK
Stopford, C.	St Mary's Hospital, Manchester, UK
Stout, J.	Monash University, Victoria, Australia
Sturrock, A.	University of British Columbia, Vancouver, Canada
Tobin, A.	CHDI, USA
Valabrègue, R.	APHP, Hôpital Salpêtriere, Paris, France
van den Bogaard, S.J.A.	Leiden University Medical Centre, Leiden, Netherlands
van der Grond, J.	Leiden University Medical Centre, Leiden, Netherlands
Wang, C.	University of Iowa, Iowa City, IA, USA
Whitehead, D.	University College London, London, UK
Whitlock, K.	Indiana University, IN, USA
Wild, E.	University College London, London, UK
Witjes-Ane, M.N.	Leiden University Medical Centre, Leiden, Netherlands

V.2. Enroll-HD investigator list

Site	Country	Name
AarhusUnivHosp	Denmark	Anette Torvin Møller
AarhusUnivHosp	Denmark	Louise Hasselstrøm Madsen
AucklandCityHosp	New Zealand	Richard Roxburgh
AucklandCityHosp	New Zealand	Virginia Hogg
AucklandCityHosp	New Zealand	Richard Roxburgh
AucklandCityHosp	New Zealand	Virginia Hogg
AugustaUniv	USA	John Morgan
AugustaUniv	USA	Paula Jackson
AvonWiltMenHeaPartTr	UK	Lesley Gowers
AvonWiltMenHeaPartTr	UK	Carol Hall
AyrshireHealthBoard	UK	Sharon Mulhern
AyrshireHealthBoard	UK	Margo Henry
AyrshireHealthBoard	UK	Tim Johnston
AziendaOspedSanAndre	Italy	Michela Ferraldeschi.
AziendaOspedSanAndre	Italy	Giovanni Ristori
AziendaOspedSanAndre	Italy	Silvia Romano
BaylorCollMed	USA	Ami Patel
BaylorCollMed	USA	Christine Hunter
BaylorCollMed	USA	Joseph Jankovic, MD
BeaumontHosp	Ireland	Ms Fiona O'Donovan
BeaumontHosp	Ireland	Prof Orla Hardiman
BeaumontHosp	Ireland	Dr Sinead Maguire
BeaumontHosp	Ireland	Dr Samira Bouazzaoui
BeaumontHosp	Ireland	Niall Pender
BirmSolNHSFounTrust	UK	Ellice Parkinson
BirmSolNHSFounTrust	UK	Hugh Rickards

BostonMedCtr	USA	Raymond James
BostonMedCtr	USA	Marie Saint-Hilaire
BurgosFoun	Spain	Esther Cubo
BurgosFoun	Spain	Natividad Marisccal
CardiffUniv	UK	Anne Rosser
CardiffUniv	UK	Rebecca Cousins
CardiffUniv	UK	Thomas Massey
CardiffUniv	UK	Duncan McLauchlan
CardiffUniv	UK	Monica Busse
CenterMovDis	Canada	Jonielyn Carlos
CenterMovDis	Canada	Kimberly Thompson
CenterMovDis	Canada	Mark Guttman
CentHospUnivMontreal	Canada	Lyne Jean
CentHospUnivMontreal	Canada	Sylvain Chouinard
CentManHospFounTrust	UK	Zara Skitt
CentManHospFounTrust	UK	Siofra Peeren
CentManHospFounTrust	UK	David Craufurd
CentManHospFounTrust	UK	Dawn Rogers
CentManHospFounTrust	UK	Iris Trender-Gerhard
CentManHospFounTrust	UK	Liz Howard
CETRAM	Chile	Maria Consuelo Moos
CETRAM	Chile	Pedro Chana
ClevelandClinicFoun	USA	Anwar Ahmed
ClinTriCtrMaastricht	Netherlands	Mayke Oosterloo
ClinTriCtrMaastricht	Netherlands	Mirella Davies-Waber
ColumbiaUniv	USA	Ronda Clouse
ColumbiaUniv	USA	Massood Manoochehri
ColumbiaUniv	USA	Sarah Janicki
ColumbiaUniv	USA	Pietro Mazzoni
ColumbiaUniv	USA	Elan Louis
ColumbiaUniv	USA	Karen Marder
ColumbiaUniv	USA	Paula Wasserman
CooperHealth	USA	Amy Colcher
CooperHealth	USA	Andrew March
CopernicusPodLec	Poland	Agnieszka Konkel
CopernicusPodLec	Poland	Witold Soltan
CrucesHosp	Spain	Koldo Berganzo Corrales
CrucesHosp	Spain	Maria Angeles Acera Gil
DukeUniv	USA	Peggy Perry-Trice
DukeUniv	USA	Burton Scott
EmoryUniv	USA	Elaine Sperin
EmoryUniv	USA	Jaime Hatcher-Martin
EmoryUniv	USA	Stewart Factor
FifeHealthBoard	UK	Gareth Thomas
FifeHealthBoard	UK	Nicola Johns
GeorgeHuntingtonInst	Germany	Herwig Lange
GeorgeHuntingtonInst	Germany	Laura Dornhege
GeorgeHuntingtonInst	Germany	Paula Raulet
GeorgeHuntingtonInst	Germany	Ralf Reilmann
GeorgeHuntingtonInst	Germany	Stefan Bohlen
GeorgetownUniv	USA	Karen Anderson
GeorgetownUniv	USA	Natasha Scott

GreatGlasgowHealthBoard	UK	Catherine Deith
GreatGlasgowHealthBoard	UK	Dr. Stuart Ritchie
GuyandStThomFounTrust	UK	Deborah Ruddy
GuyandStThomFounTrust	UK	Dene Robertson
GuyandStThomFounTrust	UK	Alison Lashwood
GuyandStThomFounTrust	UK	Elizabeth White
GuyandStThomFounTrust	UK	Thomasin Andrews
HNDC	USA	Gregory Suter
HNDC	USA	William M Mallonee
HospCreuSantPau	Spain	Andrea Horta
HospCreuSantPau	Spain	Jaime Kulisevsky
HospInfantChrisBadaj	Spain	Carmen Durán Herrera
HospInfantChrisBadaj	Spain	Patrocinio García Moreno
HospMareMerce	Spain	Elvira Roca Goma
HospMareMerce	Spain	Jesús Miguel Ruíz Idiago
HospUnivBellvitge	Spain	Matilde Calopa
HospUnivBellvitge	Spain	Jordi Bas
InstNeuroBuenoAires	Argentina	Emilia Gatto
InstPsychandNeuro	Poland	Grzegorz Witkowski
InstPsychandNeuro	Poland	Iwona Stepniak
JimenDiazFoun	Spain	Pedro J Garcia Ruiz
JimenDiazFoun	Spain	Asunción Martinez
JohnsHopkinsUniv	USA	Frederick C. Nucifora Jr.
JohnsHopkinsUniv	USA	Christopher Ross
JohnsHopkinsUniv	USA	Mollie Jenckes
KboIsarAmpKlinTauf	Germany	Alzbeta Mühlbäck
KboIsarAmpKlinTauf	Germany	Matthias Dose
KboIsarAmpKlinTauf	Germany	Michael Bachmaier
KboIsarAmpKlinTauf	Germany	Ralf Marquard
KrakowskaAkademiaNeuro	Poland	Monica Rudzinska
KrakowskaAkademiaNeuro	Poland	Natalia Grabska
LeedsTeachHospTrust	UK	Alison Kraus
LeedsTeachHospTrust	UK	Stuart Jamieson
LeedsTeachHospTrust	UK	Ivana Markova
LeedsTeachHospTrust	UK	Emma Hobson
LeedsTeachHospTrust	UK	Callum Schofield
LegaItalRiceHunt	Italy	Massimo Marano
LegaItalRiceHunt	Italy	Simone Migliore
LegaItalRiceHunt	Italy	Sabrina Maffi
LegaItalRiceHunt	Italy	Barbara D'Alessio
LegaItalRiceHunt	Italy	Ferdinando Squitieri
Leicestershire	UK	Dawn Freire-Patino
Leicestershire	UK	Caroline Hallam
Leicestershire	UK	Reza Kiani
LeidenUniv	Netherlands	Raymund Roos
LeidenUniv	Netherlands	Marye Hogenboom
LomaLindaUniv	USA	Dharmaseeli Moses
LothianHealthBoard	UK	Philip Greene
LothianHealthBoard	UK	Marie McGill
LothianHealthBoard	UK	Mary Porteous
MilanGenetic	Italy	Anna Castaldo
MilanGenetic	Italy	Caterina Mariotti

MilanGenetic	Italy	Lorenzo Nanetti
MilanNeuro	Italy	Dominga Paridi
MilanNeuro	Italy	Paola Soliveri
MilanNeuro	Italy	Simona Castagliuolo
MinnMedResFoun	USA	Dawn Radtke
MinnMedResFoun	USA	Martha Nance
MonashUniv	Australia	Dr. Andrew Churchyard
MonashUniv	Australia	Katie Fitzgerald
MonashUniv	Australia	Julie Stout
NHSForthValley	UK	Christian Neumann
NHSForthValley	UK	David Thomson
NorStaffCombHeaTrust	UK	George El-Nimr
NorStaffCombHeaTrust	UK	Karen Kennedy
NorthBristolTrust	UK	Dr Catherine Pennington
NorthBristolTrust	UK	Serena Dillon
NorthBristolTrust	UK	Elizabeth Coulthard
NorthBristolTrust	UK	Louise Gethin
NorthMetroHlthServ	Australia	Jacenta Abbott
NorthMetroHlthServ	Australia	Peter Panegyres
NorthumbTyneFreeman	UK	Jill Davison
NorthumbTyneFreeman	UK	Suresh Komati
NorthumbTyneFreeman	UK	Sarah Edwards
OhioStateUniv	USA	Allison Daley
OhioStateUniv	USA	Sandra Kostyk
OhioStateUniv	USA	Katherine Ambrogi
OxfordUnivHospTrust	UK	Professor Andrea H Nemeth
OxfordUnivHospTrust	UK	Sarsha Wilson
PlyHospNHSTrust	UK	Julie Frost
PlyHospNHSTrust	UK	Dr. Rupert Noad
PlyHospNHSTrust	UK	Leanne Timings
PooleHospFounTrust	UK	Annemieke Fox
PooleHospFounTrust	UK	John Burn
PoznanUniv	Poland	Daniel Zielonka
PoznanUniv	Poland	Elżbieta Alicja Puch
RamonCajalUnivHosp	Spain	José Luis López-Sendón Moreno
RamonCajalUnivHosp	Spain	Verónica Mañanes Barral
RockyMtnMovDis	USA	Jessica Jaynes
RockyMtnMovDis	USA	Rajeev Kumar
RoyalDevExetFounTrst	UK	Sarah Irvine
RoyalDevExetFounTrst	UK	Timothy Harrower
RoyBerkNHSFounTrust	UK	Anita Foster
RoyBerkNHSFounTrust	UK	Dr. Richard Armstrong
RushUniv	USA	Courtney Timms
RushUniv	USA	Jennifer Goldman
RutgersUniv	USA	Daniel Schneider
RutgersUniv	USA	Deborah Caputo
SanfordResearch	USA	Tish Skarloken
SanfordResearch	USA	Tanya Harlow
SanfordResearch	USA	Destini Spaeth
SchleswigHolsteinHosp	Germany	Sandra Bloess
SchleswigHolsteinHosp	Germany	Alexander Münchau
SchleswigHolsteinHosp	Germany	Jenny Schmalfeld

SchleswigHolsteinHosp	Germany	Klaus Gehring
SchleswigHolsteinHosp	Germany	Vera Tadic
SheffieldChildFouTru	UK	Anya Kholkina
SheffieldChildFouTru	UK	Oliver Quarrell
SilesianMedUnivKatowice	Poland	Klaudia Plinta
SonEspasesHosp	Spain	Penélope Navas Arques
SonEspasesHosp	Spain	Ines Legarda
SouthamptonUnivHospTrust	UK	Christopher Kipps
SouthamptonUnivHospTrust	UK	Veena Agarwal
StAndrewsHealth	UK	Elvina Chu
StGeorgeHealthTrust	UK	Nayana Lahiri
StGeorgeHealthTrust	UK	Uruj Anjum
StJosefAndElisabethHosp	Germany	Barbara Kaminski
StJosefAndElisabethHosp	Germany	Carsten Saft
StJosefAndElisabethHosp	Germany	Rainer Hoffmann
StJosefAndElisabethHosp	Germany	Sarah von Hein
Tayside	UK	Alison Tonner
Tayside	UK	Lindsay Wilson
Tayside	UK	David Goudie
Tayside	UK	Paula McFadyen
TechUnivMunich	Germany	Adolf Weindl
TechUnivMunich	Germany	Antje Lüsebrink
UnivAberdeen	UK	Daniela Rae
UnivAberdeen	UK	Alisdair Ross
UnivAberdeen	UK	Stella Sihlabela
UnivAberdeen	UK	Zosia Miedzybrodzka
UnivAlbertaGlenrose	Canada	Pam King
UnivAlbertaGlenrose	Canada	Wayne Martin
UnivBari	Italy	Marina de Tommaso
UnivBari	Italy	Vittorio Sciruicchio
UnivBologna	Italy	Cesa Scaglione
UnivBologna	Italy	Pietro Cortelli
UnivBritishCol	Canada	Allison Coleman
UnivBritishCol	Canada	Lynn Raymond
UnivBritishCol	Canada	Blair Leavitt
UnivCalDavis	USA	Alexandra (Sasha) Duffy
UnivCalDavis	USA	Amanda Martin
UnivCalDavis	USA	Ashok Joshua Dayananthan
UnivCalDavis	USA	Vicki Wheelock
UnivCalgary	Canada	Lorelei Tainsh (Derwent)
UnivCalgary	Canada	Sarah Furtado
UnivCalIrvine	USA	Nicolas Phielipp
UnivCalIrvine	USA	Durk Thompson
UnivCalIrvine	USA	Breana Chew
UnivCalSanDiego	USA	Jody Corey-Bloom
UnivCalSanDiego	USA	Sungmee Park
UnivCalSanDiego	USA	Ajay Nathan
UnivCalSanFran	USA	Alexandra Nelson
UnivCambridge	UK	Dr Sarah Mason
UnivCambridge	UK	Dr Caroline Williams-Gray
UnivCambridge	UK	Anna Gerritz (nee Di Pietro)
UnivCambridge	UK	Roger Barker

UnivCattolicaSacrCur	Italy	Flavia Torlizzi
UnivCattolicaSacrCur	Italy	Anna Rita Bentivoglio
UnivCattolicaSacrCur	Italy	Marcella Solito
UnivCharite	Germany	Josef Priller
UnivCharite	Germany	Anika Langenfurth
UnivCharite	Germany	Markus Beuth
UnivChicago	USA	Joan Young
UnivChicago	USA	Tao Xie
UnivCincinnatiPhysCo	USA	Andrew Duker
UnivCincinnatiPhysCo	USA	Katie Krier
UnivCollLondon	UK	Ed Wild
UnivCollLondon	UK	Monica Lewis
UnivCollLondon	UK	Nicola Robertson
UnivCollLondon	UK	Sarah Tabrizi
UnivConnHealthCtr	USA	Bonnie Hennig
UnivConnHealthCtr	USA	Kevin James Manning
UniverMedCtrFreiburg	Germany	Gerit Kammel
UniverMedCtrFreiburg	Germany	Stephan Klebe
UniverMedCtrFreiburg	Germany	Michel Rijntjes
UnivGroningen	Netherlands	H.P.H. Kremer
UnivGroningen	Netherlands	Jesper Klooster
UnivHospAachen	Germany	Beate Schumann
UnivHospAachen	Germany	Johannes Schiefer
UnivHospAachen	Germany	Kathrin Reetz
UnivHospCopenhagen	Denmark	Christina Vangsted Hansen
UnivHospCopenhagen	Denmark	Jørgen Nielsen
UnivHospCopenhagen	Denmark	Lena E. Hjermind
UnivHospCopenhagen	Denmark	Suzanne Granhøj Lindquist
UnivHospCopenhagen	Denmark	Peter Roos
UnivHospErlangen	Germany	Susanne Seifert
UnivHospErlangen	Germany	Christina Kozay
UnivHospErlangen	Germany	Zacharias Kohl
UnivHospGiessenMarburg	Germany	Katrin Bürk
UnivHospOdense	Denmark	Lene Wermuth
UnivHospOdense	Denmark	Marianne Dybro Lundsgaard
UnivHospUlm	Germany	Hela Jerbi
UnivHospUlm	Germany	Jan Lewerenz
UnivHospUlm	Germany	Michael Orth
UnivHospUlm	Germany	Panteha Fathinia
UnivHospUlm	Germany	Patrick Weydt
UnivHospWuerzburg	Germany	Kerstin Nöth
UnivHospWuerzburg	Germany	Christine Leypold
UnivHospWuerzburg	Germany	Kai Boelmans
UnivIllinois	USA	Mitch King
UnivIllinois	USA	Sadie Foster
UnivIowa	USA	Angel L. Dominguez
UnivIowa	USA	Jane S Paulsen
UnivKansasMedCtrResInst	USA	Carolyn Gray
UnivKansasMedCtrResInst	USA	Richard Dubinsky
UnivMaryland	USA	Terra Hill
UnivMaryland	USA	William Keller
UnivMich	USA	Elizabeth Sullivan

UnivNaples	Italy	Luigi di Maio
UnivNaples	Italy	Cinzia Valeria Russo
UnivNaples	Italy	Silvio Peluso
UnivNaples	Italy	Elena Salvatore
UnivNaples	Italy	Giuseppe De Michele
UnivOtago	New Zealand	Laura Paermentier
UnivOtago	New Zealand	Tim Anderson
UnivOtago	New Zealand	Laura Paermentier
UnivOtago	New Zealand	Tim Anderson
UnivPitt	USA	Larry Ivanco
UnivPitt	USA	Valerie Suski
UnivRochester	USA	Amy Chesire
UnivRochester	USA	Frederick Marshall
UnivRochester	USA	Julia Iourinets
UnivSouthFlorida	USA	Danielle Hergert
UnivSouthFlorida	USA	Patricia Johnson
UnivSouthFlorida	USA	Emily Kellogg
UnivSouthFlorida	USA	Juan Sanchez-Ramos
UnivSouthFlorida	USA	Kelly (Kollen) Elliott
UnivTenn	USA	Dr. Mark LeDoux
UnivTenn	USA	Amanda Nolte
UnivTexasHlthCntrHous	USA	Erin Furr Stimming
UnivTexasHlthCntrHous	USA	Leigh Beth Latham
UnivUtah	USA	Meghan Zorn
UnivUtah	USA	Matthew Halverson
UnivUtah	USA	Stefan Pulst
UnivUtah	USA	Paola Wall
UnivVermont	USA	Emily Houston
UnivVermont	USA	James Boyd
UnivVirginia	USA	Katie L. Sullivan
UnivVirginia	USA	Susan Dietrich
UnivWarsaw	Poland	Piotr Janik
UnivWarsaw	Poland	Zygmunt Jamrozik
UnivWash	USA	Ali Samii
UnivWash	USA	Debra Del Castillo
VanderbiltUniv	USA	Daniel O. Claassen
VanderbiltUniv	USA	Lauren West
VanderbiltUniv	USA	Onyebuchi Okeke
VirginiaCommUniv	USA	Claudia Testa
VirginiaCommUniv	USA	Ginger Norris
WakeForestUniv	USA	Christine O'Neill
WakeForestUniv	USA	Francis Walker
WaltonCtrFounTrust	UK	Louise Pate
WaltonCtrFounTrust	UK	Rhys Davies
WashingtonUniv	USA	Joel S. Perlmutter
WashingtonUniv	USA	Stacey Barton
WashingtonUniv	USA	Elaine Most
WestSydneyHlthDist	Australia	Dr. Clement Loy
WestSydneyHlthDist	Australia	Jillian McMillan
WestSydneyHlthDist	Australia	Therese Alting
